# Highlights in Resistance Mechanism Pathways for Combination Therapy

**DOI:** 10.3390/cells8091013

**Published:** 2019-08-30

**Authors:** João M. A. Delou, Alana S. O. Souza, Leonel C. M. Souza, Helena L. Borges

**Affiliations:** Institute of Biomedical Sciences, Federal University of Rio de Janeiro, Rio de Janeiro 21941-902, Brazil

**Keywords:** resistance mechanisms, hallmarks of cancer, targeted therapy, cytotoxic chemotherapy, drugs mechanisms of action, cell death

## Abstract

Combination chemotherapy has been a mainstay in cancer treatment for the last 60 years. Although the mechanisms of action and signaling pathways affected by most treatments with single antineoplastic agents might be relatively well understood, most combinations remain poorly understood. This review presents the most common alterations of signaling pathways in response to cytotoxic and targeted anticancer drug treatments, with a discussion of how the knowledge of signaling pathways might support and orient the development of innovative strategies for anticancer combination therapy. The ultimate goal is to highlight possible strategies of chemotherapy combinations based on the signaling pathways associated with the resistance mechanisms against anticancer drugs to maximize the selective induction of cancer cell death. We consider this review an extensive compilation of updated known information on chemotherapy resistance mechanisms to promote new combination therapies to be to discussed and tested.

## 1. Introduction

Cancer chemotherapy has evolved greatly since the first clinical trial using nitrogen mustard in 1942 [1]. This single-agent treatment, or monotherapy, gave rise to the study, screening, and development of several other small molecules as anticancer candidates. In the 1960s, the use of the combination of Vincristine, Amethopterin, 6-Mercaptopurine, and Prednisone (VAMP) for pediatric leukemia markedly changed the stigmatic status of cancer chemotherapy to “curable”, showing drastic increments in remissions and survival. It also made evident the superiority of drug combination over monotherapy [2]. However, the underlying mechanisms for the efficacy achieved with this combination therapy were unclear at the time and, in part, remain so today. Combination therapy aims to hamper cancer cell homeostasis/metabolism at multiple simultaneous targets to improve its therapeutic efficacy, reduce dosage, reduce side effects, and prevent or delay the development of acquired resistance [3]. However, acquired resistance will still eventually develop along with the treatment in response to the exposure to antineoplastic drugs. 

Tumor cells can develop drug resistance due to intrinsic factors, such as mutations, translocations, epigenetic elements, and extrinsic factors, such as hypoxia, pH, hormones, cytokines from its microenvironment surroundings, and antineoplastic agents [4]. The tumor heterogeneity and mutational load have been directly associated with the emergence of acquired resistance to antitumor drugs [5,6]. Chemotherapeutic agents from diverse drug classifications have been combined to bypass multiple factors of drug resistance. 

There is a growing concern with the chemical structure-based classification generally used by pharmacology texts and the restricted mindset it provides in combinatorial preclinical studies. The classification of drugs as cell cycle-dependent or independent was the first phenotype-based classification of antitumor agents [7,8]. Biological targets [9], biological activities [10], and structural prediction of antiproliferative activity [11] have also been proposed to facilitate insights into new drug combinations. Here we harvested the target information and biological activity to propose a rationale for drug combination.

Recent work with an RNAi-based high-throughput screening of combinations of antitumor drug libraries in cultures of almost every cancer type cell known [12] has reached a surprising conclusion. Pritchard and coauthors have claimed that most of the combination protocols approved since are, in fact, almost as good as single-agent treatments, with some few or almost negligible mechanistic contributions from single-drug components. Very few combinations exhibited real synergism. The authors suggested that each drug might affect different heterogeneous subpopulations. When the drug cocktail affects the same cell, the convergence of downstream signaling pathways related to cell death might limit the efficiency of the combined treatment [12].

The use of medications with different targets but with some overlap in their resistance-related signaling pathways might improve efficiency. Here we propose a strategy for combination studies that one of the drugs used in the antitumor cocktail should be an inhibitor of a known resistance signaling pathway of one or more drugs used in combination. The purpose of the discussion is to improve the discovery of drug combination studies. To improve the understanding of specific resistance mechanisms of widely used anticancer drugs is necessary. The information on resistance mechanisms, signaling pathways, and mechanisms of action for cytotoxic and target therapy is summarized in tables and discussed below.

## 2. Search Strategy

We highlighted the most clinically relevant information from selected articles (Pubmed), clinical trials (ClinicalTrials.gov), and the curated databases Drugbank [13] and SuperCYP/Transformer [14] relating to drug resistance mechanisms.

Drug names were used as keywords, as well as, but not restricted to target, off-target, mechanism of action, resistance, mechanism of resistance, signaling pathway, detoxification, elimination, metabolism, pharmacokinetics, pharmacodynamics, absorption, distribution, binding, clinical trial, combination, synthetic lethality, drug combination, Food and Drug Administration (FDA) approval, screening, combination screening, and drug screening.

Although extensive pharmacokinetic studies are required for the approval of a drug for human use, it is not mandatory to completely elucidate every single tissue or plasmatic detoxification enzyme, which can lead to some unclear or incomplete information [15]. In the absence of off-target ligands recognized, we have listed some off-target effects that might contribute to the development of innovative therapeutic strategies.

## 3. Resistance Mechanisms and Signaling Pathways

Cytotoxic Chemotherapy, at its most simplistic, means the use of chemical compounds to kill cancer cells more effectively than non-tumoral cells [2]. As the knowledge of cancer biology expands, drug development in cancer research could migrate from the discovery of natural compounds to the design of synthetic drug candidates that aim at exclusive or specific cancer targets in what is known as Targeted Therapy [2]. Targeted drug development has become an exciting field for academics and the pharmaceutical industry since imatinib approval in 1995. At the present time, there are twice as many targeted drugs (~120) approved by the FDA than cytotoxic small molecules (~60) (Supplementary Table S1; Table 2).

All FDA approved small molecule cytotoxic antineoplastic drugs available as at May 2019 are listed in Supplementary Table S1, and all available targeted antineoplastic agents approved by the FDA up to May 2019 are presented in Supplementary Table S2. 

One of the most appealing differences between Cytotoxic and Targeted Therapy is in the development of specific acquired resistance mechanisms. If ATP-binding cassette (ABC) transporters, enzymatic detoxification, and DNA homeostasis proteins mostly affect cytotoxic drugs, the targeted antineoplastic agents do not. These conclusions can be reached by a close examination of Supplementary Tables S1 and S2. The summary of the most common specific resistance mechanisms for both cytotoxic and targeted therapies is presented in Table 1. The signaling pathways associated with resistance to antitumor small molecules and targeted therapies are briefly discussed below.

## 4. ABC Transporters

As seen in Table 1, the main cause of resistance to cytotoxic drugs is the overexpression of ABC transporters in the plasma membrane of cancer cells. Both intrinsic and acquired resistance can be specific to a certain drug, or nonspecific, covering a broad spectrum of drugs. When the spectrum of the resistance is so broad that it covers drugs with unrelated structures and targets in such a way that it is not possible to make a clear association between them, it is called multidrug resistance (MDR). ABC transporters were the first and most studied mechanism of resistance associated with clinical MDR [16,17]. MDR can be understood from either a cellular or a clinical point of view.

From a cellular perspective, resistance might be a consequence of the low intracellular concentration of the cytotoxic drug. ABC transporters can bind anticancer drugs either from the surroundings of the plasma membrane or intracellular vesicles, and transport them out of the cell directly to the external milieu or through exocytosis [18].

Clinically, MDR is the main cause of failure in the treatment with cytotoxic drugs [19], which can be explained in part, at least, by the extensive overexpression of several ABC transporters and its long list of substrates among antineoplastic agents (mitomycin, chlorambucil, methotrexate, pemetrexed, 5-fluorouracil, mercaptopurine, estramustine, docetaxel, paclitaxel, vinblastine, vincristine, vinorelbine, daunorubicin, doxorubicin, epirubicin, idarubicin, mitoxantrone, irinotecan, topotecan, dactinomycin, and etoposide—as seen in Supplementary Table S1). 

For several years, detection and inhibition of a minimum panel of clinically relevant ABC transporters have been attempted [17,19]. However, it is clear that intra-tumor heterogeneity also affects the number of possible combinations of transporters regulated in different subpopulations. Some leukemia cohorts, for example, have shown overexpression of multiple ABC transporters among their patients [20,21,22]. The Cancer Genome Atlas (TCGA) data have also been used to show these expression level discrepancies among patients and cancer types [17].

The clinical use of ABC transporter inhibitors is long hampered by the severe toxicities presented. Numerous ABC transporter inhibitors have been clinically tested in the last forty years [17,19,23]. Recently, fourth-generation ABC transporter inhibitors have been developed, mostly based on natural and semi-synthetic compounds [24].

The development of reliable and validated methods for the adequate detection and quantification of ABC transporters is an ongoing challenge [17,19]. Nonetheless, the importance of these transporters in limiting drug delivery, affecting clinical outcomes, and the increased expression of ABC transporters in several cancers, justifies the considerable effort [19]. 

## 5. Enzymatic Detoxification

The second cause of resistance to cytotoxic drugs is the increased activity or expression of specific detoxification enzymes of each drug (Table 1). For example, Bleomycin, which can be used for cervical, head and neck, lymphomas, penile, testicular, and vulvar cancer [25], showed bleomycin hydrolase [26,27,28] as a mechanism of detoxification, and the increased activity of bleomycin hydrolase, N-acetylating enzymes, and bleomycin-binding proteins [27,28,29], as mechanisms of resistance (Supplementary Table S1). 

In addition to specific detoxification enzymes of each drug, glutathione S-transferase (GST) shows a broad detoxification effect. GST participates in the detoxification of several antineoplastic drugs by binding a glutathione molecule to it, therefore promoting some inactivation and increasing the affinity to some ABC transporters, especially from ABCC and ABCG families [30,31,32].

## 6. DNA Homeostasis-Related Signaling Pathways and Cytoskeletal Disruptors

In addition to increased activity of detoxification, mutations, overexpression, and downregulation of key targets, namely topoisomerase I and tubulin isoforms, are the next most common alterations. These are alterations that affect two major families, the topoisomerase inhibitors and cytoskeletal disruptors, which are among the most used cytotoxic drugs. Tubulin directly participates in the formation of the metaphase spindle and separation of sister chromatids, and when this is not adequately completed, leads to cell death by mitotic catastrophe [33,34]. Changes in isotype expression [35], mutations in [35,36,37] and/or overexpression of tubulins [35], and post-translational modifications of tubulins have been observed in many cancers [38].

Since most cytotoxic drugs are genotoxic, it is not surprising to have proteins associated with DNA homeostasis among the most common resistance mechanisms (Table 1). Therefore, several common resistance mechanisms in response to cytotoxic drugs are associated with DNA homeostasis (topoisomerases, tubulin, dCK, NF-κB, MGMT, ALDH1, and TP53). These molecular pathways are crucial key points to cytotoxic treatment resistance and will be discussed briefly.

Topoisomerases are vital regulators of DNA topology during DNA replication, transcription, repair, and recombination. Type I or II topoisomerases produce reversible single- or double-strand breaks, respectively. Their current pharmacological inactivation is related to stalled replication fork and cytotoxic DNA fragmentation by the formation of irreparable covalently bound DNA–protein–drug ternary complexes. Several other classes of topoisomerase inhibitors are under development, but none have presented tolerable toxicity, acceptable specificity, and potency with proper pharmacokinetics [39,40]. 

dCK is a key enzyme, usually the rate-limiting one in the synthesis of deoxynucleosides in the salvage pathway, a crucial alternative compensatory pathway for deoxynucleotide synthesis when the de novo pathway is inhibited or downregulated [41,42]. dCK overexpression is a key component to resistance to nucleoside analogs (cladribine, clofarabine, cytarabine, decitabine, gemcitabine, nelarabine) (Supplementary Table S1). 

Increased DNA methyltransferase (MGMT) protein levels are one of the most important mechanisms of resistance to alkylating agents because it can repair the cytotoxic O6-methylguanine DNA adduct and prevent its harmful effects, which affect therapy efficiency [43]. For example, when tumors showed methylation on the MGMT promoter, glioblastoma patients treated with the alkylating agent temozolomide and radiotherapy showed 21.7 months of median survival, compared to only 15.3 months among those who were assigned to radiotherapy only. In the absence of methylation of the MGMT promoter, there is no statistical difference in survival between the treatment groups, regardless of temozolomide treatment [44]. This suggested that patients with wild type MGMT have nearly no response to the alkylating agent. One may speculate that increasing the methylation status might have some benefit to enhance the cell death response induced by alkylating agent treatment in glioblastomas. After DNA damage, tumor suppressor TP53 is induced to regulate cell cycle arrest, DNA repair, and apoptotic cell death. Deletions, nonsense, and frameshift mutations that lead to its loss-of-function are common across a vast range of cancer cell type tumorigenesis. Gain-of-function oncogenic mutations are also common and represent a different mechanism for which *TP53* can participate in tumorigenesis. When these oncogenic mutations are present in cancer, *TP53* expression is usually associated with a more aggressive form of the disease, with increased tumor genome instability and metastatic potential (recently reviewed in [45]). Altogether, *TP53* mutations are found in nearly 50% of human cancers, and they are one of the classically associated resistance mechanisms against cytotoxic therapy.

## 7. Activation of NF-κB

NF-κB is a small family of five proteins with two effector transcription factor complexes, p65–p50, and p52-RelB [46]. These complexes are respectively associated with the canonical and non-canonical signaling pathways. The canonical pathway is activated under the control of cell receptors to several pro-inflammatory cytokines, tumor necrosis factor (TNF), lipid polysaccharides, growth factors, and antigens, whereas, the non-canonical pathway is triggered by Lymphotoxin beta receptor (LTBR), cluster of differentiation 40 (CD40), B-cell activating factor receptor 3 (BR3), and RANK. Both pathways are limited by the initial participation of cytosolic inactivating complexes. The canonical pathway involves the IKB and IKK inhibitory proteins, and the non-canonical recruit only IKK proteins. The activation of the receptors for these pathways triggers a sequence of specific phosphorylations and ubiquitinations that rapidly and transiently release the active form of their respective transcription factor complexes, which translocate to the nucleus where they effectively activate hundreds of validated transcriptional targets [46]. Because of the huge plethora of target genes and our poor understanding of this complex orchestra, most clinically approved inhibitors have an incomplete description of their mechanisms of action. The NF-κB pathway participates in cellular immunity, inflammation, apoptosis, cell differentiation, proliferation, and response to stress. In cancer, activation of NF-κB rescues the cell from the apoptotic pathway, promoting its survival, preventing cell death, and promoting proliferation [46]. 

## 8. Increased Levels of ALDH1

Several types of cancer stem cell (CSC) populations have elevated aldehyde dehydrogenase activity (ALDH), which converts toxic aldehydes into carboxylic acids [47]. ALDH enzymatic activity supports cancer stem cell self-renewal, protection against oxidative stress, and participation in energetic metabolism as a reliable alternative source of nicotinamide adenine dinucleotide (NADH), a convenient substrate for ATP synthesis. They also confer resistance to selected anticancer agents by metabolic inactivation and have been implicated in every tumorigenic process, from initiation to metastasis [48,49]. Therefore, it is not surprising that an increased level of ALDH is one of the specific resistance mechanisms associated with cytotoxic therapy.

## 9. Signaling Pathways Associated with Targeted Therapies

Differently from cytotoxic drugs, the resistance mechanisms of target therapy are mostly specific key alterations in their signaling pathway targets, such as mitogen-activated protein kinase (MAPK) pathway (also known as RAS-RAF-MEK-ERK pathway), phosphoinositide 3-kinase pathway (PI3K-AKT-mTOR), epidermal growth factor (EGF), EGF receptor (EGFR), phosphatase and tensin homolog deleted on chromosome 10 (PTEN), insulin-like growth factors (IGFs), key regulators of apoptosis B-cell lymphoma 2 (BCL2) family, fibroblast growth factors (FGFs), and signal transducers and activators of transcription (STATs). Except for the overexpression of ABC transporters, which occurs but with less frequency than for cytotoxic drugs, the major mechanism of action of targeted therapy is key alterations in their specific signaling pathway targets (Table 1). Briefly, these signaling pathways can be associated with Survival (PI3K, AKT, mTOR, STAT), Proliferation (MAPK, STAT, growth factors—FGFs, EGFR, IGFs), and Cell Death (BCL2 family and PTEN).

Therefore, the initial excitement surrounding targeted therapy might be diminished by the nature of the therapy itself. From one perspective, single-agent targeted therapy inhibits specific dysregulated pathways. Such treatment would represent a strong but punctual selective pressure over cancer cell populations [50]. The strong specificity allows reduced toxicity and promotes efficient clinical response for those patients who might benefit from the treatment. On the other hand, targeted therapy is also largely limited by this strong specificity in the face of the multiple alterations cancer cells present [6], and which are necessary to circumvent the redundant signaling pathways that prevent tumorigenesis [51]. The efficacy of the therapy is also hampered by the large intra- and inter-tumor heterogeneity [52]. These limitations might be seen by the relatively small benefit some patients present from the intended targeted therapy and by the relative transient remission, accompanied by relapse, which is often and usually together with acquired resistance [53]. All these points have been the theme of some nice discussions in recent works [50,54,55,56].

One key aspect to circumvent the emergence of resistance might be to “spread” the selective pressure made by antineoplastic treatment attacking multiple pathways simultaneously, possibly by the combination of multiple drugs [19,56]. This must be performed according to already established guidelines such as (i) each single agent should be effective in monotherapy; (ii) use agents with different mechanisms of action, preferably on different subcellular structures and/or different phases of the cell cycle; (iii) avoid overlapping toxicities, particularly over vital organs or with life-threatening side effects; (iv) optimize dose and schedule of treatment and intervals to improve both efficacy and minimize toxicities; and, finally, (v) have a clear understanding of the mechanism of interaction among drugs [57,58,59,60]. The last of these is particularly poorly understood. This statement is supported by a relatively recent study [12]. 

## 10. Resistance Mechanisms can be Associated with the Hallmarks of Cancer Cells

When resistance mechanisms and signaling pathways are associated with specific hallmarks of cancer, it became evident that some hallmarks are mostly associated with oncogenes (genome instability, survival, proliferation, angiogenesis); meanwhile, others are almost tumor suppressor exclusives (evasion of inhibitory factors, cell death). Classic oncogene alterations are associated with gain-of-function in which one single genetic alteration is enough to over-activate the oncogenic signaling pathway; meanwhile, two-hit tumor suppressors are often associated with inactivating mutations and downregulation of key signaling elements of the pathway. It seems that the two-hit requirements for an effective intervention on a tumor suppressor pathway have limited the development of tumor suppressor-based therapies since most targeted therapies are aimed at oncogenes (Supplementary Table S2).

Cytotoxic and targeted therapies increased detoxification and specific signaling pathway mechanisms, respectively. Interestingly, both mechanisms can be associated with the hallmarks of cancer cells. Whereas increased detoxification mechanisms can be associated with altered metabolism of cancer cells, the signaling pathways induced by target therapy as resistance mechanisms can be associated with all the hallmarks. In Table 2, there is an association between the specific resistance mechanisms listed in Supplementary Tables S1 and S2 and the most affected signaling pathways in cancer cells, as well as their corresponding hallmarks of cancer. 

Some cellular proteins and processes are major regulators of cell homeostasis, which make them largely associated with several, if not all, hallmarks. Some examples are NF-κB [46], PTEN [61], and autophagy [62,63]. NF-κB was briefly mentioned above.

## 11. Signaling Pathways Related to the Hallmark Evasion of Growth Suppression

PTEN is one of the most commonly affected tumor suppressors proteins during tumorigenesis [64]. Its gene encodes a tumor suppressor phosphatase protein that physiologically prevents the entry to the cell cycle, as well as G2/M transition and mitosis. Regulation of the cell cycle by PTEN has been recently reviewed elsewhere [65]. PTEN arrests cells at G1 by inactivating the PI3K/AKT pathway, thus inhibiting cell cycle progression through the regulation of several signaling molecules, including decreased levels of cyclin D and inactivation by phosphorylation of the retinoblastoma protein (RB). RB regulates the restriction point, the G1-late stage when cells become committed to proliferate [66]. Cell cycle progression relies on the phosphorylated state of RB, which is tightly controlled by cyclin-dependent kinases (CDKs).

Upon mitogenic stimulation, cyclin D-CDK4/CDK6 phosphorylates RB, thereby inactivating it and promoting the release of transcription factors of the E2F family, which activate the transcription of target genes required for cell cycle progression [67]. Dysregulation of this canonical RB function is central in cancer, with components of the CDK4/6-RB pathway often displaying mutations that will result in sustained cell proliferation; for instance, in tumors that retain RB expression, uncontrolled cell cycle activity may be due to the amplification of the cyclin D1 gene (*CCND1*) and *CDK4*, activating *CDK4/6* mutations or silencing of CDK inhibitors (CKI) [67,68].

There are 13 members of the CDK family, from which only four—CDK1, CDK2, CDK4, and CDK6—are direct regulators of the cell cycle [69]. *CDK4* and *CDK6* have been implicated as drivers of oncogenesis in several cancers [70] for more than 20 years.

There are two major families of physiological CKI that regulate the cell cycle through cyclin-CDK activity in animals: INK4 and CIP/KIP families. They are both allosteric inhibitors of CDKs [71].

RB is also regulated by phosphorylation. It has been recently proposed that in a non-cycling cell, when RB is in its “active” form sequestering E2F family members, there are multiple mono-phosphorylated isoforms of RB (mP-RB) simultaneously present [72]. The authors showed functional differences between mP-RBs beyond the regulation of the cell cycle machinery. It is yet unknown which proteins participate in the complexes to each type of mP-RBs, which proteins are regulated by each isoform, in which situations these isoforms are selectively regulated, which isoforms are functionally distinct or redundant, and so on. There is also evidence for hypo- and hyper-phosphorylated isoforms, which have been largely studied by other groups [73,74].

## 12. CDK Inhibitors

Pharmacological specific inhibition of CDKs has been long pursued. The most promising early CDK inhibitor was the pan-inhibitor CDK flavopiridol (or alvocidib) [72,75,76,77]. Flavopiridol was tested in more than 60 clinical trials for 15 years, mostly with disappointing degrees of antitumor activity [76,78]. Flavopiridol did not obtain approval, which was attributed to limited clinical efficacy [79,80,81,82] and severe toxicities [80,82].

Selective CDK4/6 inhibitor compounds were developed, clinically tested, and recently approved for human use, namely, palbociclib (PD0332991, Pfizer, NY, USA), ribociblib (LEE011, Novartis, Basel, Switzerland) and abemaciclib (LY2835219, Eli Lilly, Indianapolis, USA). These specific CDK have raised much attention and excitement since their first approvals. Palbociclib was the first to receive its approval. It was indicated for first-line use among post-menopausal women with advanced hormone positive HER2 negative breast cancer who have not been previously treated with systemic chemotherapy [83]. In April 2019, its approval was also extended for the treatment of men with hormone positive HER2 negative metastatic breast cancer [84].

Recent reviews of the clinical trials with these selective CDK4/6 inhibitors and their impact on clinical outcomes have discussed great advances in median progression-free survival, and objective response ratio among hormone receptor positive breast cancer patients treated with all three approved CDK [85,86].

There are some similarities between the approvals of palbociclib [83], ribociclib [87], and abemaciclib [88] for breast cancer treatment. All three drugs were first approved for advanced hormone positive HER2 negative breast cancer patients in combination with hormone therapy (letrozole with palbociclib or ribociclib, and fulvestrant/abemaciclib), for which they presented synergistic growth inhibitory activity. The approvals were granted based mostly on progression-free survival and objective response ratio, without data on overall survival. The larger cohort among the three trials enrolled 165 women, a small cohort for survival purposes. Moreover, the three approvals are upfront treatments aiming mostly at disease control before systemic chemotherapy is given, which, although more aggressive, is also a more established curative approach.

The full potential of selective CDK is under investigation in many preclinical and clinical studies [85]. As target therapies, CDK treatment produces some specific resistances. Overcoming these predictive resistances might greatly improve clinical outcomes as recently discussed elsewhere [89].

Preclinical and clinical data support the notion that the rational treatment with cell cycle phase-specific antitumor agents might produce synergic cytotoxic effects depending on the choice and order of drugs to be administered. For example, sequential administration of CDK followed by DNA damaging cell cycle specific agents have been tested in a panel of TP53 mutant and wild-type breast and human colorectal cancer cell lines. The authors used roscovitine, a purine-based nonselective CDK, followed by doxorubicin [90]. The CDK inhibition increased apoptosis induced by doxorubicin only on TP53 null or mutant cell lines, highlighting the ability of wild type TP53 to prevent cell cycle synthetic lethality due to DNA damaging agents. The authors also reported superior benefits in terms of overall survival and decreased proliferation in human breast cancer xenografts treated with the sequential regime of roscovitine followed by doxorubicin over the concomitant combination (*p* < 0.0001) or either drug alone (*p* < 0.01) [90]. It is worth testing similar types of experiments with CDK4/6 selective inhibitors.

## 13. Signaling Pathways Related to Cell Death

In addition to sustained proliferation and evasion of growth suppression, resistance to cell death is one of the three pivotal drivers of tumorigenesis [91]. From analyzing Table 2, one can promptly realize that many of the same signaling disruptions are exploited by cancer cells and allow resistance to anticancer therapy. Importantly, programmed cell death (PCD), triggered by several external and internal stress signals, must be overcome if both tumorigenesis and drug resistance are to be successful. The best-characterized form of PCD is apoptosis, which is composed by two pathways—one activated by death receptor signaling and the other by intracellular, mitochondrial signaling—that converge at the level of effector proteases called caspases. These will execute an extensive intracellular proteolytic program, leading to apoptotic cell death. Disruptions in the mitochondrial (or “intrinsic”) apoptotic program are widely involved in cancer progression and therapy resistance [91]. The ultimate trigger to caspase activation in the intrinsic program is the release of mitochondrial factors, such as cytochrome c, through mitochondrial outer membrane permeabilization (MOMP). This process is tightly regulated by a dynamic balance among the members of the BCL2 protein family at the outer mitochondrial membrane (OMM): the pro-apoptotic BCL2 proteins, Bax and Bak, directly promote MOMP by forming pores at the OMM, which are usually inhibited by binding of the anti-apoptotic (e.g., BCL2, BCL2-like protein 1, and MCL1/BCL2L3) BCL2 proteins [92]. Additionally, BH3-only proteins (e.g., Bid, Bad, Bim, Noxa, and Puma) directly inhibit anti-apoptotic BCL2 proteins and/or directly activate Bax/Bak, thus inducing MOMP and triggering caspase activation. When activated, the tumor suppressor TP53, best known for its surveillance activity regarding DNA damage, promotes the expression of several genes regulating the intrinsic pathway, mainly *Bax* and *PUMA*, ultimately also leading to caspase activation [93]. Perhaps unsurprisingly, mutations leading to enhanced anti-apoptotic BCL2 signaling, downregulation of Bax and Bak, and p53 inactivation, promote drug resistance across many cancer types (Supplementary Tables S1 and S2).

## 14. Induction of Autophagy is a Common Cellular Phenomenon associated with Cell Death Resistance

Autophagy is an evolutionarily conserved process of elimination and recycling “damaged” or “old” cytoplasmic cell components, structures, and organelles in lysosomes [94]. Autophagy has been recently associated with several hallmarks of cancer [63]: sustained proliferation (energetic source); promotion of epithelial–mesenchymal transition; sustained survival during cell migration (evasion of anoikic cell death) and metastasis (facilitates tumor cell dormancy and quiescence, survival and proliferation during new tumor formation); pro-survival alternative energetic source of dysregulated aerobic metabolism; evasion of cell death (evasion of apoptosis, survival of residual cancer stem cells after chemotherapy); and, genome instability (sustains DNA damage repair system) [95,96].

Physiologically, basal autophagy is a recycling energetic system for the turnover of proteins and lipids, which might be increasingly activated in response to a stressor, like starvation. Therefore, it might be seen as a pro-survival process for any normal or cancerous cells. Under prolonged periods of starvation, a regulated programmed cell death (PCD) process known as autophagic cell death is observed [97]. As briefly discussed above, there are many possible opportunities for cancer therapy based on pharmacological modulation of autophagy and to promote increased cancer cell death. We can and should take advantage of these phenomena [98,99].

Autophagy plays its role in cancer by regulating several pathways which rule over cell life and death, such as BCL2, Class III and I PI3K (PI3K-I and PI3K-III), AKT, mTORC ½, and TP53 [100]. Despite the complexity of the mTOR signaling network, mTORC1 is a well-defined central autophagy inhibitor, acting as a point of convergence for many pathways: the pro-survival PI3K-I-AKT and MAPK pathways induce mTORC1, whereas AMP-activated protein kinase (AMPK) signaling inhibits it [101]. mTORC1 signaling is also induced by growth factors via the PI3K-I-AKT and MAPK pathways.

Furthermore, TP53 also presents a “Janus role” of its own in autophagy, both inducing (cytoplasmic p53) and inhibiting (nuclear p53, through AMPK) mTORC1 activity. Autophagy induction and enhanced PI3K-AKT-mTOR and MAPK signaling pathways are often related to resistance against a wide range of drugs in several cancer types, which is further evidence of its importance during tumorigenesis (Supplementary Table S2 and Table 2). Furthermore, it is important to have in mind the significant cross-talk between autophagy and apoptosis signaling [102]: As well as inhibiting autophagy, growth factor signaling, for example, it will also induce the JAK/STAT1/3 pathway, which will, in turn, induce anti-apoptotic BCL2 signaling.

**Figure 1:**
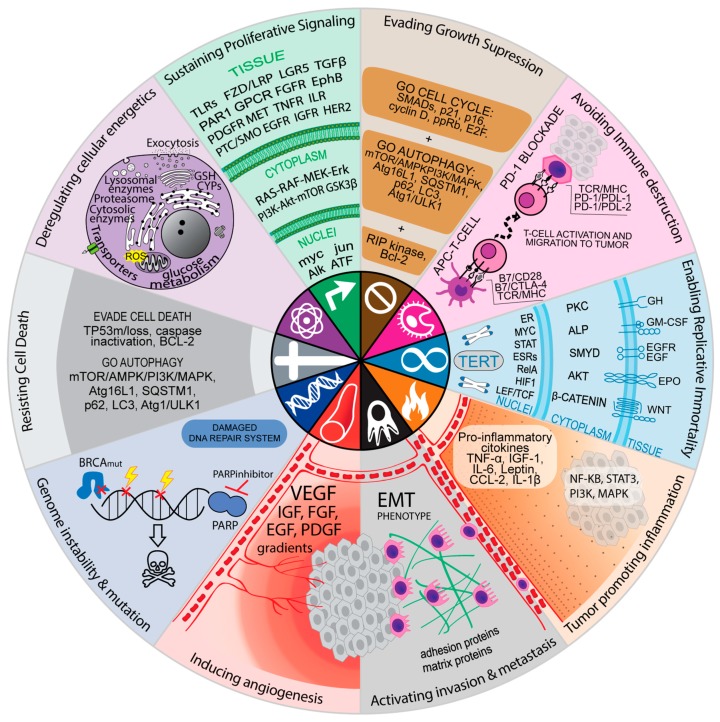
Targets of approved or investigational pharmacological intervention on signaling pathways associated with resistance mechanisms against antineoplastic agents and their associations with the hallmarks of cancer. The hallmarks of cancer [91] are sustaining proliferative signaling, evasion of growth suppression, avoiding immune destruction, enabling replicative immortality, tumor-promoting inflammation, activation invasion and metastasis, inducing angiogenesis, genome instability and mutation, resisting cell death, and deregulating cellular energetics. The most commonly affected signaling pathways in response to cytotoxic or targeted chemotherapeutic agents related to specific resistance mechanisms are depicted. For more details, please consult the supplementary tables. Adapted from Hanahan and Weinberg (2011) [91].

The very nature of the network connection of the major signaling pathways, both affected during the initial tumorigenic process and therapy-induced, is very complex. The complexity is not only due to many connection points, but also because they affect various cellular processes related to all hallmarks of cancers. This sophistication makes the topic interesting and intriguing, as can be seen in Figure 1, where cell signaling is summarized according to cancer markers.

Another level of the additional complexity of the signaling network is evidenced by the fact that proteins can show multiple roles in the cell. For example, RB and β-catenin, both known to be involved in proliferation, have been described by regulating cell death according to the context of tumor cells [103,104].

## 15. Clinical Trials with Combination Regimens Containing Inhibitors of Signaling Pathways Related to Drug Resistance

The concept of increased cell death by blocking the resistance mechanisms that protect cancer cells against systemic chemotherapy is relatively simple and intuitive. Similarly, increased cell death is to be expected when combining anticancer drugs in a cocktail in which one of its drugs might act as an inhibitor of the signaling pathways related to the known resistance mechanisms against one or some other anticancer drugs in combination. The general simplest idea of combining cytotoxic and targeted chemotherapy is usually attributed for target therapy somehow sensitizing cells to increased cytotoxicity (Table 3). Meanwhile, the rationale for combining two targeted drugs might be much more variable and require a case-by-case evaluation (Table 4). Most combination studies between targeted therapies do not aim at the resistance mechanisms affected by the drugs in the combination. We have only found five examples that fit into this strategy. We searched among already registered clinical trials for examples of drug combinations in which a specific targeted drug might inhibit the signaling pathway-related to the resistance to the second targeted combined drug (Table 4). With this rationale, we found five ongoing phase I, six ongoing phase II, three phase I completed, and two phase II completed (Table 4).

Several clinical trials that investigate mechanism-based targeted drug combinations are ongoing. They present a rationale for dual inhibition of one or more pathways that could increase cell death (Table 4). Some combinations represent a dual blockade in pathways related to different hallmarks of cancer (Figure 1), such as the combination of bevacizumab and erlotinib for advanced liver cancer, which targets both tumor neovascularization and proliferative signaling (EGFR and vascular endothelial growth factor dual blockade, Table 4).

Another example of mechanism-based targeted drug combinations is ongoing for pancreatic tumors. Since the enhanced PI3K/mTOR activity confers CDK4/6 inhibitor resistance therapy, the inhibition of those pathways should be tested (Table 4). This is an interesting ongoing clinical trial that is currently evaluating the combination of ribociclib, a CDK4/6 inhibitor, and everolimus, an mTOR inhibitor in metastatic pancreatic adenocarcinoma patients refractory to 5-fluorouracil (5-FU) and gemcitabine-based chemotherapy [105]. Their cancers have either intrinsic or acquired resistances to first and second lines of treatment. The antiproliferative combination might synergize to increase cell death, which would be unlikely to happen with either targeted therapies alone. mTOR is one of the potent resistance mechanisms against CDK inhibitors (Supplementary Table S2). Its inhibition with everolimus is a strategy for inhibition of the cross-talk between the pro-survival PI3K-AKT-mTOR pathway and the proliferative RAS–RAF–MEK–ERK signaling pathway. This rationale has already presented some nice results in breast [106] and prostate cancers [107].

The dual-hit inhibition strategy has some proven benefits over single pathway inhibitors. Some dual hit combinations have been approved for different pathways and cancer types, such as dual inhibition of EGFR, HER2, and MAPK. These combinations trigger specific but distinct mechanisms of resistance. In EGFR and HER-2, for example, improved efficacy is seen without added or more severe toxicities [108]. 

## 16. Intrinsic Toxicity and Compensatory Mechanism of Inhibitors of Key Signaling Pathways of the Resistance Mechanisms 

Many inhibitors of key signaling pathways of the resistance mechanisms can show high toxicity and compensatory mechanisms, as well as several potential targets of the same pathways. For example, somatic alterations in the MAPK pathway that are highly prevalent in human cancer and are also related to therapy resistance, show a wide range of targeted inhibitions (see Supplementary Table S2 and Table 1). Currently, there are no rat sarcoma protein (RAS) inhibitors available, but monotherapy of downstream selective MAPK inhibitors shows great promise, with some examples of already approved regimens. 

Selective monotherapy with rapidly accelerated fibrosarcoma (RAF) inhibitors has greatly improved clinical progress in melanoma patients [168]. However, intra-pathway resistance mechanisms still arise, often related to RAF-independent extracellular signal–regulated kinases (ERK) activation. In this context, v-Raf murine sarcoma viral oncogene homolog B (BRAF) inhibitor, dabrafenib therapy has triggered rapidly-growing skin tumors, which can be partially mitigated by combination with trametinib, a mitogen-activated protein kinase kinase (MEK) inhibitor [169]. This downstream inhibition of the pathway reduces possibilities for ERK reactivation. As of May 2019, the FDA has approved a total of three BRAF/MEK combination regimens, with the dabrafenib and trametinib combination being indicated for the treatment of BRAF-V600-positive melanoma, non-small cell lung cancer and anaplastic thyroid cancer (Table 4). 

There are other inhibition strategies targeting MAPK that aim to offer improved therapy. Early clinical studies of RAF/MEK inhibition combined with immunotherapy, for example, have faced interruption or patient discontinuation due to severe life-threatening toxicities [170], with evidence suggesting that the combination potentiates adverse effects of both strategies when used alone. Larger studies are expected to better define this strategy. This highlights the importance of avoiding accumulated overlapping toxicity in the rationale of the combination to be tested in trials, as seen with other targeted pathways. Importantly, while there are no FDA-approved selective ERK inhibitors, preclinical studies have already shown that ERK mutations and expression imbalances might arise as resistance mechanisms [171]. Recent clinical data suggest that ERK inhibitors therapy-related toxicities are not severe, which opens the possibility for combinations with upstream RAF/MEK inhibition and greater clinical success for the future of MAPK inhibition therapy [172].

The PI3K-AKT-mTOR pathway is one of the most frequently dysregulated pathways, not only in tumor development but as the mechanism of resistance after treatment (Table 1). Approximately 50 compounds targeting some of its key proteins have been in clinical development over the years [173]. Although these compounds have reached different stages of clinical trials, a promising response has not been observed as with other approved targeted therapies [174]. For a number of reasons, including high toxicity, only a few PI3K-AKT-mTOR pathway inhibitors have been approved by the FDA and indicated for cancer treatment: four PI3K inhibitors—idelalisib, copanlisib and, more recently, duvelisib and alpelisib (September 2018 and May 2019, respectively); one AKT inhibitor—miltefosine; and, two mTOR inhibitors—temsirolimus and everolimus. 

Inhibition of this signaling pathway has proven to be a double-edged sword between potency and toxicity. Anti-PI3K and anti-mTOR monotherapies, although well-tolerated, have shown only modest efficacy in several clinical trials [173]. On the other hand, combination therapies targeting multiple PI3K-AKT-mTOR components are usually more effective but lead to a build-up of dose-limiting toxicities [173]. Most severe toxicities related to PI3K inhibitors might be explained by severe immune modulations in several organs. Many reviews have recently addressed this complex problem [175].

Generally, preclinical studies have shown that isoform-specific targeting of PI3K has better therapeutic efficacy and toxicity profiles than pan-inhibitors [174]. The same is true for direct AKT inhibition. Most AKT inhibitors in clinical development are pan-inhibitors, with many clinical trials suspended due to severe hyperglycemia [176]. The efficacy of the AKT inhibitors TCN, TCN-P, and edelfosine is limited due to their toxicity [177]. Miltefosine was the only AKT inhibitor approved by the FDA in 2014, indicated for visceral, cutaneous, and mucosal leishmaniasis. In cancer, miltefosine has limited use mainly due to its gastrointestinal and hemolytic toxicities. Therefore, it has been used as a topical formulation to treat cutaneous lesions caused by lymphoma and cutaneous breast cancer metastasis [178]. Given the central role of AKT signaling dysregulation in cancer, the development of safer AKT inhibitors is urgent.

Regarding mTOR inhibition, hematologic toxicities of various types were observed in the clinical trials that resulted in FDA approval of temsirolimus: 94% had hemoglobinemia, 53% had lymphocytopenia, 19% had neutropenia, and 40% had thrombocytopenia [179]. Both temsirolimus and everolimus display immunosuppressive activity, derived from B and T cell proliferation inhibition from rapamycin, the molecule of which they are analogs [180]. As such, their toxicity profiles also include infections, hypersensitivity reactions, angioedema, nephrotoxicity with proteinuria, kidney arterial and venous thrombosis, delays in wound healing, and increased risk of second tumors and increased risk of opportunistic infections [180]. These rare to severe side effects are dose-limiting, but rarely lead to discontinuation of mTOR inhibitor therapies because either adjusting the dose or support medications can overcome them.

## 17. Synthetic Lethality

The recognition of an effective chemotherapy regimen, either monotherapy or in combination, has been achieved in clinical trials, which are largely based on clinical and empirical experience, with a focus on therapeutic efficacy and tolerable toxicity. This is an expensive and time-consuming pipeline, for which most drugs fail [181]. Mechanism-based drug combinations have been mostly investigated at the preclinical stages. The search for better combinations has developed many screenings, such as those that aim at synthetic lethality and drug repurposing.

Synthetic lethality screenings have been thoroughly performed for the last 10 years in the search for gene and drug synergism. These screens have identified some combinations that might result in true drug interactions, from which one single combination has reached a place in the clinic, namely, the use of poly(ADP-ribose) polymerase (PARP) inhibitors in BRCA1 DNA repair associated (*BRCA*) gene mutated patients [182]. Inhibition of PARP activity induces synthetic lethality in mutated *BRCA1/2* cancers by selectively targeting tumor cells that fail to repair DNA double-strand breaks [183]. 

Another promising example of the capabilities of synthetic lethality screening has been published for late ovarian cancer; the authors proposed a list of 84 new drug combinations to be tested in preclinical and clinical trials [184]. 

We strongly believe that the expansion in knowledge of signaling pathways and networks will present innovative opportunities for improved cancer therapies.

## 18. Drug Repurposing

Only 5% of anticancer drugs entering phase I clinical trials are ultimately granted FDA approval [185]. Drug repurposing aims to identify new applications for approved or investigational drugs that differ from their original clinical indication. This strategy allows for faster development at reduced costs since preclinical and clinical data might already be available for the repurposed drug [186]. Several analgesics and anesthetics, and antipsychotic, antibiotic and antiprotozoal drugs, among many other classes, have already been repurposed or are being tested on an oncology setting, based on modulation of numerous cell signaling pathways commonly disrupted in cancer [185]. Nitazoxanide (NTZ) is an antiprotozoal drug, which has been recently shown to inhibit autophagy in glioblastoma cells [187]. The combination with chloroquine (CQ), a well-known antimalarial autophagy inhibitor, had a synergistic effect, with CQ sensitizing the glioma cells to NTZ. Since NTZ has been shown to cross the blood–brain barrier (BBB) in mice, this highly lipophilic compound is a potential drug for reuse to treat gliomas [187]. 

Lipophilic antipsychotic drugs, known to cross the BBB effectively to bind to central dopamine D2 receptors and promote their therapeutic action, are able to modulate many cancer-associated intracellular signaling pathways, such as PI3K-AKT-mTOR, STAT3, and WNT [188], and may act as anti-cancer drugs. Chlorpromazine (CPZ), a dopamine D2 receptor antagonist, is able to cross the cell membrane and bind to FKBP-12, inhibiting the mTOR pathway, one of the most frequent points of dysregulation in cancer (Supplementary Table S3). This inhibition led to increased autophagic cell death in U-87 MG (glioblastoma) cells. Furthermore, CPZ inhibited tumor growth in human xenograft colon cancer and induced apoptosis in CRC cells in a p53-dependent manner mediated by c-Jun N-terminal Kinase (JNK) activation [189]. 

Other dopamine receptor-independent pharmacological activities of antipsychotics may be used as a basis for drug repurposing. Serotonin 5-hydroxytryptamine 7 (5-HT7) receptors are expressed by astrocytes and are commonly found overexpressed in glioblastoma, and are associated with apoptosis resistance, pro-survival signaling, and malignant transformation [188]. Thus, the use of antipsychotic drugs that also bind and inhibit 5-HT7, the antipsychotic risperidone (RIS), could be an attractive strategy for glioblastoma treatment. Other potential anticancer properties of RIS have already been demonstrated in both in vitro and in murine models. In resistant breast and colorectal cancer models, RIS is able to inhibit ABCG2 overexpression in a dose-dependent fashion, thus being able to reduce the impact of resistance mechanisms dependent on drug efflux that may arise from long-term chemotherapy [188]. 

Given the poor availability of efficient treatments in many cancer types (Supplementary Table S3), repurposing of such drugs given in combination with traditional chemotherapy and/or radiotherapy could improve cancer treatment and lead to lower doses and side effects, ultimately improving prognosis and quality of patients’ lives.

## 19. Conclusion and Final Considerations

Cytotoxic drugs are genotoxic, so the most common resistance mechanisms are associated with DNA homeostasis and increase detoxification as ABC transport. The resistance mechanisms of the targeted therapy are specific key changes in their targets of the signaling pathways, such as MAPK, PI3K, AKT, mTOR, and others. It is expected that the combination of chemotherapy and drugs intended to inhibit the mechanism of resistance induced by chemotherapy would increase cell death. This approach has the limitation that most inhibitors of ABC transport and MAPK receptors, PI3K, AKT, and mTOR, showed high toxicity and were not recommended for phase III clinical trial. The development of more selective inhibitors and repurposing of existing drugs are two different strategies that could be used to address this toxicity. 

For the first strategy, a few examples have already been FDA approved. Initial clinical trials with pan-CDK inhibitors were very toxic and, as a consequence, did not reach phase III. Recently, three selective CDK4/6 inhibitors have been approved for metastatic breast cancer and are now being tested in various combinations for different tumors (Table 3 and Table 4). Many pre-clinical and clinical studies are necessary to understand when combinations of CDK inhibitors and other therapies may be promising since both antagonist and synergic effects can be achieved [190]. Even for the new generation of specific CDK4/6 inhibitors, the combination with first- and second-line cytotoxic therapy in glioblastomas showed antagonist and synergic effects depending on the combination. Abemaciclib increased cell death in temozolomide-treated groups, whereas it decreased carboplatin-induced cell death in glioblastoma cell lines (Hadju et al. unpublished data). In addition to new combinations of drugs given simultaneously, alternative regimes of sequential therapies should be explored depending on the possible biological effects that can be induced. Since CDK4/6 inhibitors induce transient cell cycle arrest, they might be suitable to be used in sequential cycles. Once the cell cycle blockage is released, cytotoxic drugs might maximize DNA damage-induced cell death of synchronized S-phase cells. Therefore, profound understandings of cell biology may help in designing new strategies. 

Another possibility for dealing with the high toxicity of inhibiting key components of signaling pathways of resistance mechanisms is to explore less potent inhibitors in the form of safer drugs already in use for other purposes. For this, the careful off-target studies of drugs already in use for another proposal can be tested. For example, imatinib was originally designed to inhibit BCR-ABL tyrosine kinase, and it was identified that it also inhibits platelet-derived growth factor receptor (*PDGFR*) and *KIT* proto-oncogene tyrosine kinases. Since *KIT* and *PDGFRA* mutations are observed in 85% of gastrointestinal stromal tumors (GISTs), it was also tested and approved for GIST treatment [191]. The list of repurposed drugs can be found on the reproDB website [192]. It is urgent, however, that drug combinations are tested based on the mechanism of action, off-target, and resistance mechanisms, including drugs already designed for another purpose. This review collected the known off-target and resistance mechanism data of cytotoxic drugs (Supplementary Table S1) and anticancer-targeted therapies (Supplementary Table S2) that were FDA-approved up to May 2019, organized by drug classification. This effort hopefully will help build future experiments exploring new combinations of drugs, including known off-targets that interfere directly with resistance mechanisms of other drugs.

The effort of looking for new combinations that target the resistance mechanisms should not be limited to anticancer drugs but should also include repurposing drugs for other diseases. For example, lipophilic antipsychotic drugs and others that can inhibit the major mechanism of resistance of the target therapy, such as PI3K-AKT-mTOR and STAT3, [188] can be tested with a wide range of target therapies and cancers (Supplementary Table S2). Studies are particularly needed for tumors whose patients present a 5-year mean survival that is below 20% and have limited or even no FDA-approved combinations (Supplementary Table S3).

It is worth noting that the literature search for the resistance mechanisms discussed in this review was limited to the direct effectors of signaling pathways. However, let us not forget that resistance mechanisms might also be triggered by indirect modulation of signaling pathways, such as transcription factors and regulatory elements (enhancers and silencers, regulatory RNAs—lncRNA, miRNA). Moreover, most indirect pathways represent network-signaling interactions in which they might be tumor- or cell-type specific, with putative different triggers and modulations by specific co-repressors and/or co-activators. These also represent relevant targets for drug development, but more research will be needed, including high-throughput screening. 

Bioinformatics and many preclinical studies are needed to understand the mechanisms of drug interactions for combination therapy. Since most signaling pathways are overlapping, with several points of interaction with other pathways, and show compensatory mechanisms, it is certainly a difficult task to predict this complex network, especially when particular mutations of tumor cells are also taken into account. One may wonder whether it is not more efficient to screen patients to the best protocols available instead of using a "fit the patient to the drug” approach, an unintended design of clinical trials for drug development. Would not it be beneficial, ethical, and possibly more economical for patients and drug development to avoid unnecessary exposure to inefficient protocols? We understand that both traditional trials and personalized therapy might (or should) be supported by individual, fast, and efficient targeting and/or drug screening, before any first-line treatments to identify intrinsic resistances and sensitivities. This might be achieved by designing faster high-throughput screenings with patient-derived tissue samples, such as first-generation organoid cultures [193]. Affordable protocols for organoid cultures of the major tissues affected by human cancers are urgently needed.

## Figures and Tables

**Table 1:** Most common specific resistance mechanisms and signaling pathways associated with cytotoxic and targeted therapies.

Top	Cytotoxic Drugs (*N* = 59)	Drugs Affected	%	Targeted Drugs (*N* = 117)	Drugs Affected	%
1	ABC transporters	21	36	MAPK family	34	29
2	Enzymatic detoxification	9	17	PI3K-AKT-mTOR	33	28
3	Mutation in and/or downregulation of topoisomerases I/II	7	12	EGF and EGFR	21	18
4	Mutation in and/or overexpression of tubulins	6	10	PTEN	14	12
5	Decreased dCK	6	8	ABC transporters	14	12
6	Increased activity of GST	5	8	IGFs	14	12
7	Activation of NF-κB	4	7	JAK/STAT	14	12
8	Increased MGMT	4	7	BCL-2 family	13	12
9	Increased levels of ALDH1	3	5	FGFs	12	11
10	Silencing or mutations in TP53	3	5	ERBB2 (HER2)	12	11

N = FDA approved antineoplastic drugs available as at May 2019. Abbreviations: ABC—ATP-binding cassette, dCK—deoxycytidine kinase, GST—glutathione s-transferase, NF-κB—factor nuclear kappa B, MGMT—O-6-methylguanine-DNA methyltransferase, ALDH1—aldehyde dehydrogenase 1 family, TP53—tumor protein p53, MAPK—mitogen activated protein kinases, PI3K—phosphoinositide 3-kinase, AKT—protein kinase B, mTOR—mammalian target of rapamycin, EGF—epithelial growth factor, EGFR—epithelial growth factor receptor, PTEN—phosphatase and tensin homolog, IGF—insulin-like growth factor 1, JAK—Janus kinase, STAT—signal transducer and activator of transcription, BCL2—B-cell lymphoma 2, FGF—fibroblast growth factor, ERBB2—erb-b2 receptor tyrosine kinase 2, HER2—human epidermal growth factor receptor 2.

**Table 2:** Hallmarks of cancer and signaling pathways associated with specific resistance mechanisms.

Top	Cytotoxic (*N* = 59)	Drugs Affected	%	Targeted therapies (*N* = 117)	Drugs Affected	%
**1**	**Metabolism** (Detoxification transporters and enzymes, protection from ROS, increased glucose metabolism)	21	36	**Sustaining Proliferative Signaling** (GF, Hedgehog, MAPK, PI3K, WNT, autophagy induction)	65	56
**2**	**Sustaining Proliferative Signaling** (GF, Hedgehog, MAPK, PI3K, WNT, autophagy induction)	19	32	**Cell Death Evasion** (BCL2 family, TP53, MDM2, PTEN, NF-KB, autophagy induction)	41	35
**3**	**Cell Death Evasion** (BCL2 family, TP53, MDM2, PTEN, NF-KB, autophagy induction)	10	17	**Angiogenesis** (EGF, IGF, FGF, VEGF, PDGF)	31	26
**4**	**Genome instability** (TP53, MDM2, NHEJ, RAD51, CHEK1/2, BRCA1/2, HDAC)	5	8	**Metabolism** (Detoxification transporters and enzymes, protection from ROS, increased glucose metabolism)	21	18
**5**	**Evading growth suppressors** (TP53, RB, cyclins, CDKs, p16, p18, p21)	4	7	**Genome instability** (TP53, MDM2, NHEJ, RAD51, CHEK1/2, BRCA1/2, HDAC, p21)	18	15
**6**	**Inflammation** (NF-kB)	4	7	**Immune checkpoint** (CD19, CD20, CTLA-4, NT5E, PCLP, PD-1, PD-L1)	16	14
**7**	**Angiogenesis** (EGF, IGF, FGF, VEGF, PDGF)	2	3	**Evading growth suppressors** (TP53, RB, cyclins, CDKs, p16, p18, p21)	13	11
**8**	**Immune checkpoint** (CD19, CD20, CTLA-4, NT5E, PCLP, PD-1, PD-L1)	0	0	**EMT, invasion, and metastasis** (EMT phenotype, integrin)	13	11
**9**	**EMT, invasion, and metastasis** (EMT phenotype, integrin)	0	0	**Inflammation** (NF-kB)	12	10
**10**	**Replicative immortality** (WNT, Hedgehog, TERT)	0	0	**Replicative immortality** (WNT, Hedgehog, TERT)	8	7

N = FDA approved antineoplastic drugs available as at May 2019. Abbreviations: NF-κB—factor nuclear kappa B, TP53—tumor protein p53, MAPK—mitogen activated protein kinases, PI3K—phosphoinositide 3-kinase, EGF—epithelial growth factor, PTEN—phosphatase and tensin homolog, IGF—insulin-like growth factor 1, JAK—Janus kinase, STAT—signal transducer and activator of transcription, BCL2—B-cell lymphoma 2, FGF—fibroblast growth factor, ERBB2—erb-b2 receptor tyrosine kinase 2, HER2—human epidermal growth factor receptor 2, ROS—reactive oxygen species, GF—growth factor, NHEJ—non-homologous end joining, RAD51—RAD51 recombinase, CHEK1/2—checkpoint kinases 1 and 2, BRCA1/2—breast cancer type 1 susceptibility protein, and 2, HDAC—histone deacetylase, RB—retinoblastoma protein, CDKs—cyclin-dependent kinase, PDGF—platelet-derived growth factor, CTLA-4—cytotoxic T lymphocyte antigen 4, NT5E—ecto-5′-nucleotidase, PCLP—podocalyxin-like protein 1, PD-1—programmed cell death protein 1, PD-L1—programmed death-ligand 1, EMT—epithelial–mesenchymal transition, TERT—telomerase reverse transcriptase.

**Table 3:** Clinical trials with combinations in which targeted therapies inhibit resistance mechanisms of cytotoxic drugs.

Signalling Pathway Affected	Targeted Drug/Inhibitor Drug	Cytotoxic Drugs	Clinical Trial Phase	Indications	Obs	References
**BCR-ABL**	Bosutinib	Pemetrexed	Ongoing phase I	Bladder, cervical, NSCLC, ovarian	Recruiting	[109]
	Dasatinib	Carboplatin + Paclitaxel	Phase I completed	Ovarian	Recommended for phase II	[110]
**BCL-2**	Venetoclax	Azacitidine	Ongoing phase II	AML	Elderly patients	[111]
		Cyclophosphamide, etoposide, doxorubicin, methotrexate, 6-mercaptopurine, cytarabine	ALL	Ongoing phase II	Older patients with relapsed or refractory ALL	[112]
**CDK4/6**	Abemaciclib	Pemetrexed, or gemcitabine	Ongoing phase I	NSCLC	For stage IV patients	[113]
		Temozolomide	Ongoing phase II	Glioblastoma		[114]
	Palbociclib	Carboplatin	Ongoing phase II	Metastatic head and neck squamous cell carcinoma		[115]
		Nab-paclitaxel	Phase I completed	mPDAC	No results posted; last update in May 30, 2019.	[116]
		Temozolomide + irinotecan	Ongoing phase I	Solid tumors, neuroblastoma, medulloblastoma	For children, adolescents and young adults	[117]
	Ribociclib	Docetaxel + Prednisone	Ongoing phase Ib/II	mCRPC		[118]
		Gemcitabine	Ongoing phase I	Malignant brain tumors		[119]
		Paclitaxel + Carboplatin	Ongoing phase I	Ovarian cancer, fallopian tube cancer, peritoneal carcinoma		[120]
**DNA DAMAGE REPAIR**	Niraparib	Temozolomide	Ongoing phase Ib/II	SCLC		[121]
	Olaparib	Cisplatin	Ongoing phase I	Advanced NSCLC		[122]
	Rucaparib	Irinotecan	Ongoing phase Ib	Solid tumors	For patients with DNA repair defects in solid tumors	[123]
	Veliparib	FOLFIRI	Ongoing phase II	Pancreatic (metastatic)	Second line therapy	[124]
			Phase I completed	Gastric	Recommended for further investigation	[124]
		Irinotecan	Ongoing phase I	Breast, lung, ovarian, pancreatic, Hodgkin’s lymphoma	For cancer that is metastatic or cannot be removed	[125]
		Temozolomide	Ongoing phase I	ALL		[126]
			Phase II completed	CRC	Recommended for further investigation	[127]
		Topotecan	Ongoing phase I	Acute leukemias		[128]
**EGFR**	Cetuximab	FOLFIRI	Ongoing phase II	CRC	For patients with FcγRIIIa polymorphism and wild-type KRAS, NRAS and BRAF	[129]
		FOLFOXIRI	Ongoing phase II	Locally advanced rectal carcinoma	For EGFR wild type patients	[130]
	Necitumumab	Gemcitabine + Cisplatin	Ongoing phase II	Stage IB, II or IIIA squamous NSCLC	Neoadjuvant therapy	[131]
	Panitumumab	FOLFOX/FOLFIRI	Phase II completed	Liver (metastatic)	For patients with wild-type KRAS; no results posted; last update May 14, 2019.	[132]
		mFOLFOX6 + bevacizumab/panitumumab	Ongoing phase III	Advanced/recurrent CRC	First-line therapy for patients with KRAS/NRAS wild-type tumors	[133]
	Pertuzumab	Paclitaxel + Trastuzumab	Ongoing phase I	HER2-positive breast cancer		[134]
**PI3K-AKT-MTOR**	Copanlisib	Gemcitabine	Phase I completed	Cholangiocarcinoma	No results yet.	[135]
	Duvelisib	Fludarabine + cyclophosphamide + rituximab (FCR)	Ongoing phase Ib/II	CLL		[136]
	Everolimus	Carboplatin + Paclitaxel	Phase II completed	Melanoma	Everolimus failed to improve efficacy	[137]
		Temozolomide	Ongoing phase II	Low-grade glioma		[138]
	Idelalisib	Bendamustine + Rituximab	Phase III completed	Relapsed or refractory CLL	Improved PFS but with serious adverse events and infections	[139]
	Temsirolimus	Carboplatin + Paclitaxel	Phase II completed	Recurrent or metastatic head and neck	Recommended further investigation for PI3K/mTOR mutations	[140]
**VEGF**	Bevacizumab	Capecitabine	Phase II completed	Advanced or Metastatic Liver Cancer	"All patients presented serious adverse events; only 9.1% presented objective response"	[141]
		Carboplatin + Paclitaxel (CPB)	Phase II completed	Melanoma	Recommended for phase III	[142]
		Carboplatin + Paclitaxel + Everolimus (CPBE)	Phase II completed	Melanoma	Failed to improve PFS compared to CPB	
		FOLFIRI (+ Onvansertib)	Ongoing phase Ib/II	mCRC	Second line therapy for patients with KRAS mutation	[143]
		Temozolomide	Phase II completed	Glioma (grade II/III)	Failed to improve 1-year OS	[144]
	Lenvatinib	Paclitaxel	Ongoing phase I	Endometrial, ovarian, fallopian tube, or primary peritoneal cancer		[145]
	Ramucirumab	FOLFIRI	Ongoing phase II	Gastric	For previous failed therapy	[146]
	Regorafenib	Irinotecan	Ongoing phase II	Metastatic gastro-esophageal adenocarcinomas	Second line therapy	[147]
	Sorafenib	Irinotecan	Ongoing phase II	Pediatric solid tumors	Patients with mutations in Raf, PDGFR, VEGFR, Flt-3, KIT, JAK, STAT, RAS, MEK, or ERK	[148]

Abbreviations: OS—overall survival, FOLFIRI—folinic acid, 5-fluorouracil and irinotecan, FOLFIRINOX—folinic acid, 5-fluorouracil, irinotecan and oxaliplatin, CRC—colorectal cancer, mCRC—metastatic colorectal cancer, NSCLC—non-small cell lung cancer, SCLC—small cell lung cancer, ALL—acute lymphoblastic leukemia.

**Table 4:** Clinical trials with combinations between targeted therapies in which the first inhibit resistance mechanisms of the second.

Signalling Pathways	Targeted Therapy	Clinical Trial Phase	Type of Tumor	Rationale	References
**ALK AND CDK4/6**	Ceritinib + Ribociclib	Phase I completed	NSCLC	ALK+ NSCLC tumors	[149]
		Ongoing phase I	Neuroblastoma	*In vitro* synergy (lower phospho-RB1 levels only in ALK+ NB cells)	[150,151]
**MET AND AR**	Crizotinib + Enzalutamide	Ongoing phase I	mCRPC	AR inhibition upregulates MET (off-target effect for crizotinib)	[152,153]
**PDGFR AND C-SRC**	Crizotinib + Dasatinib	Ongoing phase I	Solid malignancies	Downstream effects of MET require c-Src, whose inhibition upregulates MET	[154,155]
		Phase I completed	High-grade glioma	PDGFR upregulated in gliomas (off-target for dasatinib)	[156]
**PI3K AND EGFR**	Copanlisib + Cetuximab	Ongoing phase Ib/II	HNSCC	Aberrant PI3K signaling confers resistance to cetuximab and both pathways are upregulated in HNSCC	[157,158]
**VEGFR AND MTOR**	Pazopanib + Everolimus	Ongoing phase I	Solid tumors	mTOR pathway activation confers resistance to anti-VEGF therapy	[159]
**BCR**	Idelalisib + Entospletinib	Phase II completed	Lymphoid malignancies	Synergistic effect with simultaneous inhibition of multiple kinases in the BCR pathway (PI3K and Syk), but limited by severe life-threatening adverse effects.	[160]
**CDK4/6 AND PI3K/MTOR**	Abemaciclib + LY3023414	Ongoing phase II	PDAC	Enhanced PI3K/mTOR activity confers resistance to CDKi therapy	[161]
	Ribociclib + Everolimus	Ongoing phase II	mPDAC		[105]
**CDK4/6 AND EGFR**	Palbociclib + Cetuximab	Ongoing phase II	HNSCC	EGFR overexpression is an oncogenic driver and there is either a frequent loss of CDKN2A or amplification of CCND1	[162]
**CDK4/6 AND MEK**	Palbociclib + Trametinib	Phase Ib completed	Advanced solid malignancies	CDK activity confers resistance to MEKi	[163,164]
**BRAF AND MEK**	Dabrafenib + Trametinib	Ongoing phase II	Melanoma and brain metastases	MAPK pathway reactivation confers resistance to BRAFi. Melanoma brain metastasis seem to lack ABCB1 expression. Combination can cross blood-brain barrier.	[165]
	Vemurafenib + Cobimetinib	Ongoing phase II			[166]
**VEGFR AND EGFR**	Bevacizumab + Erlotinib	Phase II completed	Advanced or Metastatic Liver Cancer	Dual blockade of tumor neovascularization and proliferative signaling.	[167]

Abbreviations: HNSCC - Head and neck squamous cell carcinoma, mCRPC - metastatic castration-resistant prostate cancer, mPDAC - metastatic pancreatic duct adenocarcinoma, NSCLC - non-small cell lung cancer, PDAC - Pancreatic duct adenocarcinoma.

## Supplementary Materials

The following are available online at https://www.mdpi.com/2073-4409/8/9/1013/s1, Supplementary Table S1. Summary of FDA-approved anticancer cytotoxic drugs at May 2019. Supplementary Table S2. Summary of FDA-approved anticancer targeted therapies at May 2019. Supplementary Table S3. Approved drug combination options are limited, even when recommended in first-line therapy for the most prevalent and fatal cancer types.
