# Supplementary material for: Highlights in Resistance Mechanism Pathways for Combination Therapy
Source: Cells. 2019 Aug 30;8(9):1013. doi: 10.3390/cells8091013 (PMC6770082; doi:10.3390/cells8091013)
Supplement: Supplementary file 1 [file cells-08-01013-s001.pdf]

Supplementary table 1. Summary of FDA-approved anticancer cytotoxic drugs at May 2019.

Classification			Targets	Name	Indication	Detox pathway	Known off-targets	Resistance mechanisms	References
Synthetic drugs	Alkylating Agents	Alkylsulfonate	DNA	Busulfan	CML	Substrate of CYP3A4, GSTA1, GSTA2, GSTM1, GSTP1, MGST2.	Unclear	Overexpression and lower phosphorylation of CDC2, enhanced stress response, inactivating mutations in the mismatch repair system (in vitro)	[1–3]
		Ethyleneimine	DNA	Altretamine	Ovarian	Unclear	Unclear	Unclear	[3]
			DNA	Mitomycin	Anal, bladder, breast, cervical, gastric, lip, mesothelioma, NSCLC, oral cavity, pancreas, peritoneum, and pharynx	Substrate of POR	Unclear	Overexpression of ABC transporters	[3,4]
			DNA	Thiotepa	Breast, ovarian, bladder	Substrate of CYP3A4	Inhibitor of BCHE, CYP2B6	Enhanced activity of GST	[3,5]
		Methylhydrazine	DNA, MAOA, MAOB	Procarbazine	HL, oligodendrogliomas, PCNSL	Substrate of CYP1B1, XDH	Unclear	Inactivating mutations in the mismatch repair system (in vitro)	[6,7]
		Nitrogen mustard derivative	DNA	Bendamustine	CLL, NHL	Substrate of CYP1A2	Unclear	Overexpression of CD69 (in vitro)	[8]

SUPPLEMENTARY TABLE 1. SUMMARY OF FDA-APPROVED ANTICANCER CYTOTOXIC DRUGS AT MAY 2019 (CONTINUATION)

Classification			Targets	Name	Indication	Detox pathway	Known off-targets	Resistance mechanisms	References
**Synthetic drugs**	Alkylating Agents	Nitrogen mustard derivative	DNA	Chlorambucil	CLL, lymphomas (HL, NHL, giant folicular and lymphosarcoma)	Substrate of GSTP1	Unclear	Overexpression of GST isoenzymes and ABCC1	[3,9]
			DNA	Cyclophosphamide	ALL, brain, breast, leukemias, malignant lymphomas, multiple myeloma, ovarian, retinoblastoma	Unclear	NR1I2	Increased levels of glutathione and increased activity of GST and ALDH1	[3,10,11]
			DNA	Ifosfamide	Lymphomas, sarcomas, testicular	Substrate of CYP2A6, CYP2B6, CYP2C8, CYP2C9, CYP2C18, CYP2C19, CYP3A4, CYP3A5, PTGS1	NR1I2; inducer of CYP2C8, inhibitor and inducer of CYP3A4	TP53 silencing, increased GST and ALDH1 activities, increased DNA repair, overexpression of thioredoxin reductase (in vitro)	[3,12]
			DNA	Mechlorethamine	Bronchogenic carcinoma, CML/CLL, lymphomas (lymphosarcoma , mycosis fungoides, polycythemia vera, palliation on HL III and IV). Also for the palliative treatment of metastatic carcinoma resulting in effusion.	Unclear	Inhibitor of GSR	Overexpression of GST isoenzymes	[3,13,14]

SUPPLEMENTARY TABLE 1. SUMMARY OF FDA-APPROVED ANTICANCER CYTOTOXIC DRUGS AT MAY 2019 (CONTINUATION)

Classification			Targets	Name	Indication	Detox pathway	Known off-targets	Resistance mechanisms	References
Synthetic drugs	Alkylating Agents	Nitrogen mustard derivative	DNA	Melphalan	Multiple myeloma, ovarian	Unclear	Inhibitor of SLC22A3	Bcl-X expression	[3,15,16]
		Nitrosurea	DNA, RNA	Carmustine	Anaplastic astrocytoma, glioblastoma multiforme, multiple myeloma	Substrate of CYP1A2	Inhibitor of GSR	Overexpression of MGMT	[3,17–20]
			DNA	Lomustine	Glioma, medulloblastoma, lymphoma	Unclear	STMN4, inhibitor of CYP2D6, CYP3A4	Overexpression of MGMT	[3,21]
			DNA	Streptozocin	Pancreas and carcinoid syndrome	Unclear	SLC2A2 ligand, antagonist of Beta-n-acetylglucosaminidase, MGEA5, inducer of ABCB1, CYP1A1, CYP1A2, CYP2E1.	Overexpression of MGMT	[22]
		Triazenes	DNA, POLA2	Dacarbazine	Melanoma, lymphomas	Substrate of CYP1A1, CYP1A2, CYP	Inhibitor of PGD	Overexpression of MGMT, Bcl-2 expression, AKT activation, NF-kB activation, VEGFR-1 expression	[3,23]
			DNA	Temozolomide (Oral dacarbazine)	Anaplastic astrocytoma, glioblastoma multiforme	Unclear	Inducer of CYP3A4	Overexpression of MGMT, Bcl-2 expression, AKT activation, NF-kB activation, VEGFR-1 expression	[23,24]

SUPPLEMENTARY TABLE 1. SUMMARY OF FDA-APPROVED ANTICANCER CYTOTOXIC DRUGS AT MAY 2019 (CONTINUATION)

Classification			Targets	Name	Indication	Detox pathway	Known off-targets	Resistance mechanisms	References
**Synthetic drugs**	Alkylating Agents / DNA adducts	Platinum-based agents	DNA	Carboplatin	Lung, ovarian and squamous cell head and neck	Substrate of ABCC2, ABCG2, ATP7A, ATP7B, GSTM1, GSTT1, GSTP1, MT1A, MT2A, NQO1, SLC31A1, SLC31A2	SOD1, inducer of MPO, XDH,	Upregulation of P-type ATPases, overexpression of MRP2, enhanced activity of NER and overexpression of BRCA2, mutation and downregulation of RAD52 (repair gene)	[3,25,26]
			DNA	Cisplatin	Bladder, cervical, esophageal, gastric, germ cell tumours (including testicular), mesothelioma, NSCLC, osteosarcoma, ovarian, SCLC, and squamous cell head and neck	Substrate of ABCB1, ABCC6, ABCG2, ATP7A, ATP7B, GSTM1, GSTP1, GSTT1, MT1A, MT2A, NQO1, SLC22A2, SLC31A1, SLC31A2 and SOD1.	A2M, ATOX1, MPG and TF. Inhibitor of BCHE, CYP2B6, CYP2C9, nat, PTGS2 and SLC22A2. Inducer of ABCC2, ABCC3, ABCC5, CYP4A11, MPO and XDH.	Upregulation of P-type ATPases, overexpression of MRP2, enhanced activity of NER and overexpression of BRCA1	[3,25,27]
			DNA	Oxaliplatin	CRC, esophageal and gastric	Substrate of ATP7A, ATP7B, ABCC2, ABCG2, CYP1A1, CYP1B1, CYP2E1, GSTM1, GSTT1, GSTP1, MPO, MT1A, MT2A, NQO1, SLC22A2, SLC22A3, SLC31A1 and SOD1.	Unclear	Alterations in the mitochondrial apoptotic pathway, enhanced autophagy, increased GSH levels, *SRBC* hypermethylation (in vitro)	[3,28–30]

SUPPLEMENTARY TABLE 1. SUMMARY OF FDA-APPROVED ANTICANCER CYTOTOXIC DRUGS AT MAY 2019 (CONTINUATION)

Classification			Targets	Name	Indication	Detox pathway	Known off-targets	Resistance mechanisms	References
**Synthetic drugs**	Antimetabolite	Antifolates	DHFR	Methotrexate	Acute leukemias, bladder, brain, breast, gestational trophoblastic disease, head and neck, lung, NHL, osteosarcoma	Substrate of ABCB1, ABCC1, ABCC2, ABCC3, ABCC4, ABCC11, ABCG2, AOX1, CYP3A4, DHFR, FPGS, FOLR1, GGH, MTHFR, SLC16A1, SLC19A1, SLC22A6, SLC22A7, SLC22A8, SLC22A11, SLC46A1, SLCO1A2, SLCO1B1, SLCO1B3, SLCO1C1, SLCO3A1, SLCO4C1, TYMS	Inhibitor of ABCC1, ABCC2, ABCC3, ABCC4, ABCC10, PGD, SLC22A8, SLC46A1	Overexpression of ABC transporters, downregulation of RFC, overexpression of DHFR, loss of function of FPGS, overexpression of TS	[3,31,32]
			DHFR, GART, TYMS	Pemetrexed	Malignant mesothelioma and NSCLC	Substrate of SLC22A8	ATIC, inducer of DCK, SLC29A1	Overexpression of ABC transporters, downregulation of RFC, overexpression of DHFR, loss of function of FPGS, overexpression of TS	[33]
			DHFR, TYMS	Pralatrexate	T-cell Lymphoma	Substrate of FPGS, SLC19A1	Unclear	decreased RFC-1 expression and increased MDR1 expression	[34]
		Nucleoside and precursor analogs	DNA, RNA, DNMT1	5-Azacitidine	Leukemias	Substrate of cytidine deaminase (CDA)	Unclear	Mutations in TET2, overexpression of BCL2	[3,35]

SUPPLEMENTARY TABLE 1. SUMMARY OF FDA-APPROVED ANTICANCER CYTOTOXIC DRUGS AT MAY 2019 (CONTINUATION)

Classification			Targets	Name	Indication	Detox pathway	Known off-targets	Resistance mechanisms	References
**Synthetic drugs**	Antimetabolite	Nucleoside and precursor analogs	DNA, RNA, TYMS	5-Fluorouracil	Anal, breast, CRC, gastric, head and neck, esophageal and pancreas	ABCC3, ABCC4, ABCC5, ABCG2, CYP1A2, CYP2A6, CYP2C8, DPYD, MTHFR, PPAT, SLC22A7, SLC29A1, TYMP, TYMS, UMPS, UPP1, UPP2	Inhibitor of CYP2C9	Overexpression of ABC transporters, overexpression of TS, enhanced activity of DPD	[3,36]
			DNA, MGMT, PNP, POLA1, POLE2, POLE3, POLE4, POLE, RRM1, RRM2, RRM2B.	Cladribine	Hairy cell leukemia, chronic lymphocytic leukemia	Substrate of DCK, ABCG2, SLC28A3.	Unclear	Decreased expression of hENT1, reduced activity of dCK, overexpression of 5'-nucleotidase (in vitro)	[3,37]
			DNA, POLA1, RRM1	Clofarabine	Acute lymphoblastic leukemia and acute myeloid leukemia	Substrate of DCK, ABCG2	Unclear	Reduced activity of dCK	[3,38]
			DNA, POLB	Cytarabine	Acute myeloid leukemia, acute lymphoblastic leukemia, chronic myeloid leukemia, lymphomas, progressive multifocal leucoencephalopathy and meningeal leukemia	Substrate of CDA, CYP3A4, DCTD, NT5E	Unclear	Downregulation of dCK, downregulation of hENT1 transporter	[3,39]
			DNA, DNMT1	Decitabine	Myelodysplastic syndrome, sickle cell anaemia (orphan), acute myeloid leukemia and chronic myeloid leukemia.	Substrate of DCK	Unclear	Mutations in TET2, higher activity of CDA combined with decreased activity of dCK	[40,41]

SUPPLEMENTARY TABLE 1. SUMMARY OF FDA-APPROVED ANTICANCER CYTOTOXIC DRUGS AT MAY 2019 (CONTINUATION)

Classification	Targets	Name	Indication	Detox pathway	Known off-targets	Resistance mechanisms	References
**Synthetic drugs** Antimetabolite Nucleoside and precursor analogs	TYMS	Floxuridine (5-FU analog)	Metastatic GI adenocarcinoma and gastric	TYMP	Inhibitor of CYP2C9	Amplification of TS and/or deletion of OPRTase	[42–44]
	DNA, POLA1, RRM1	Fludarabine	Acute myeloid leukemia, chronic lymphocytic leukemia, NHL and Waldenstrom macroglobulinaemia.	Substrate of SLC28A3, SLC29A1	Agonist of DCK	Mutations in TP53, overexpression of MYC	[3,45,46]
	DNA, RRM1, TYMS, CMPK1	Gemcitabine	Bladder, breast, nasopharyngeal, non-small cell lung, ovarian and pancreas, lymphomas and inflammatory bowel disease.	Substrate of CDA, DCK, ABCB1, ABCC10, SLC28A1, SLC28A3, SLC29A1, SLC29A2	Unclear	Low expression of hENT1, higher dCDA expression, enhanced activity of dCK	[3,47]
	HPRT1, PPAT, IMPDH1, IMPDH2	Mercaptopurine	Acute lymphoblastic leukemia, acute promyelocytic leukemia, lymphoblastic lymphoma and inflammatory bowel disease	Substrate of AOX1, TPMT, XDH, SLC22A8, ABCC4, ABCC5, SLC28A2, SLC28A3, SLC29A1, SLC29A2	Unclear	Overexpression of ABC transporters, activating mutations in NT5C2	[3,48]

SUPPLEMENTARY TABLE 1. SUMMARY OF FDA-APPROVED ANTICANCER CYTOTOXIC DRUGS AT MAY 2019 (CONTINUATION)

Classification			Targets	Name	Indication	Detox pathway	Known off-targets	Resistance mechanisms	References
Synthetic drugs	Antimetabolite	Nucleoside and precursor analogs	DNA, POLA1	Nelarabine	Acute lymphoblastic leukemia and chronic lymphocytic leukemia.	Substrate of adenosine deaminase (ADA), DCK, deoxyguanosine kinase (DGUOK)	Unclear	Decreased expression of dGK and dCK (in vitro)	[49]
			ADA	Pentostatin	Hairy cell leukemia, peripheral T-cell lymphoma (orphan), cutaneous T cell lymphoma (orphan) and chronic lymphocytic leukemia (orphan).	Unclear	Unclear	Unclear	[3]
			DNA	Thioguanine	ALL, AML	Substrate of HPRT1	Inhibitor of ABCC4	Activating mutations in NT5C2	[48]
	Proteasome inhibitor		PSMB1, PSMB5	Bortezomib	Multiple myeloma, lymphomas	Substrate of CYP1A2, CYP2C9, CYP2C19, CYP2D6, CYP3A4	Inhibitor of CYP1A2, CYP2C9, CYP2C19, CYP3A4	Enhanced stress response and overexpression of NF-κB (clinical), inhibition of apoptosis (Bax and Noxa), autophagy activation (in vitro)	[3,50]
	Ribonucleotide Reductase Inhibitor		RRM1	Hydroxyurea / Hydroxycarbamide	AML, CML, head and neck, melanoma, ovarian.	Unclear	Inhibitor of CYP2D6	Unclear	[3,51]

SUPPLEMENTARY TABLE 1. SUMMARY OF FDA-APPROVED ANTICANCER CYTOTOXIC DRUGS AT MAY 2019 (CONTINUATION)

Classification			Targets	Name	Indication	Detox pathway	Known off-targets	Resistance mechanisms	References
Natural products and semi-synthetic derivatives	Cytoskeletal Disruptors	Epothilone	Microtubules and tubulin / TUBB3	Ixabepilone	Breast	Substrate of CYP3A4	CYP3A4	Defined β-tubulin mutations	[3,52]
		Macrolide analogue	Microtubules and tubulin / TUBB1	Eribulin	Breast, Liposarcoma	Unclear	BCL2	Unclear	[53,54]
		Nitrogen mustard / Steroid	Microtubules / MAP1A, MAP2	Estramustine	Prostate	Substrate of CYP3A4	ESR1, ESR2, Inhibitor of ABCB1	Overexpression of ABC transporters	[3,55]
		Taxanes	Microtubules and tubulin / TUBB1, MAP2, MAP4, MAPT	Docetaxel	Brain, breast, head and neck, gastric, lung, prostate	substrate of CYP1B1, CYP3A4, CYP3A5, CYP3A7, ABCB1, ABCC1, ABCC2, ABCC10, ABCG2, SLCO1B3, SLC22A7;	BCL2; NR1I2; inhibitor of CYP3A4 and CYP1B1; inducer of CYP1B1	Overexpression of P-glycoprotein/ABCB1, differential expression of β-tubulin isotypes, mutations in tumour suppressor proteins and altered expression of pro- and anti-apoptotic proteins	[3,56]
			Microtubules and tubulin / TUBB1, MAP2, MAP4, MAPT	Paclitaxel	Breast, lung, pancreas, ovarian, sarcoma	substrate of ABCC2, ABCG2, SLCO1B3, SLC22A7;	BCL2, NR1I2, substrate and inducer of CYP3A4; substrate and inhibitor of ABCB1, ABCB11, ABCC10; inhibitor of ABCC1, CYP19A1 and CYP1B1	Associated with specific SNP in BCL2, overexpression of ABC transporters, β-tubulin and tubulin mutations, CYP-mediated detoxification	[3,57]
		Vinca Alkaloids	Microtubules and tubulin / TUBA1, TUBB, TUBD1, TUBE1, TUBG1	Vinblastine	Bladder, breast, HL, Kaposi's sarcoma, NHL, neuroblastoma, testicular	Substrate of ABCB1, ABCB11, ABCC1, ABCC2, CYP3A4, CYP2D6	JUN, inhibitor and inducer of ABCB1, ABCB11, ABCC1, ABCC2, ABCC6, CYP3A4, SLCO1B1	Overexpression of ABC transporters, mutations in TOPO II, TOPO II downregulation and reductase-mediated enzymatic detoxification, overexpression of βII- and βIVb-tubulin	[3,58]

SUPPLEMENTARY TABLE 1. SUMMARY OF FDA-APPROVED ANTICANCER CYTOTOXIC DRUGS AT MAY 2019 (CONTINUATION)

Classification			Targets	Name	Indication	Detox pathway	Known off-targets	Resistance mechanisms	References
Natural products and semi-synthetic derivatives	Cytoskeletal Disruptors	Vinca Alkaloids	Microtubules and tubulin / TUBB, TUBA4A	Vincristine	Acute lymphocytic leukemia (ALL), HL, NHL, neuroblastoma, rhabdomyosarcoma, Wilms' tumor,	Substrate of ABCB1, ABCB11, ABCC1, ABCC2, ABCC10, ABCG2, CYP3A4, CYP3A5, CYP3A7, RALBP1	Inhibitor of ABCB1, ABCB11, ABCC1, ABCC2, ABCC10, CYP3A4; inducer of ABCB1, ABCC3, SLC22A3, SLCO1B1, SLCO1B3	Overexpression of βIII-tubulin, downregulation of MAP2c, overexpression of ABC transporters, downregulation of γ-actin (in vitro)	[3,59]
			Microtubules and tubulin / TUBB	Vinorelbine	breast, NSCLC, others	Substrate of ABCB1, CYP3A4, CYP2D6.	Unclear	Overexpression of P-glycoprotein/ABCB1, defined β-tubulin mutations, overexpression of βIII-tubulin, overexpression of RLIP76	[60]
	Cytotoxic Antibiotics	Anthracyclines	DNA, TOP2A, TOP2B	Daunorubicin	Acute leukemias	substrate of CYP1A1, CYP3A4, ABCB1, ABCC1, XDH (xantine dehydrogenase), POR (NADPH redutase), ABCB11, ABCC6, ABCC10, ABCG2;	inhibitor of ABCB1, ABCC1, ABCC2, CYP1B1, CYP3A4; inducer of ABCB1 and CYP3A5. May also inhibit polymerase activity.	Overexpression of ABC transporters, mutations in TOPO II, TOPO II downregulation and reductase-mediated enzymatic detoxification	[61]
			DNA, TOP2A, NOLC1	Doxorubicin	ALL, bladder, breast, kaposi sarcoma, lymphomas, multiple myeloma, neuroblastoma, ovarian, Wilms tumour	Substrate of ABCB11, ABCC1, ABCC2, ABCC6, ABCG2, AKR1A1, AKR1C3, CBR1, CBR3, CYP2D6, CYP3A4, NOS1, NOS2, NOS3, NQO1, POR, Q9NUT2, RALBP1, SLC22A16, XDH.	inhibitor of ABCB1, ABCC1, ABCC3, ABCC6, ABCC10, CYP1B1, CYP2B6, CYP3A4, NDUFS2, NDUFS3, NDUFS7; inducer of ABCB1.	Overexpression of ABC transporters, mutations in TOPO II, TOPO II downregulation and reductase-mediated enzymatic detoxification	[62]

SUPPLEMENTARY TABLE 1. SUMMARY OF FDA-APPROVED ANTICANCER CYTOTOXIC DRUGS AT MAY 2019 (CONTINUATION)

Classification			Targets	Name	Indication	Detox pathway	Known off-targets	Resistance mechanisms	References
Natural products and semi-synthetic derivatives	Cytotoxic Antibiotics	Anthracyclines	DNA, TOP2A, CHD1	Epirubicin	Breast, gastric and bladder	Substrate of UGT2B7, ABCC1	Inhibitor of PLA2G4A.	Overexpression of ABC transporters, mutations in TOPO II, TOPO II downregulation and reductase-mediated enzymatic detoxification	[3,62]
			DNA, TOP2A	Idarubicin	Acute leukemias	substrate of CYP2C9, CYP2D6	Inhibitor of ABCC1.	Overexpression of ABC transporters, mutations in TOPO II, TOPO II downregulation and reductase-mediated enzymatic detoxification	[3,61]
			DNA, TOP2A	Valrubicin	Bladder	Unclear	Unclear	Unclear	[63]
			DNA, TOP2A	Mitoxantrone	AML, NHL, prostate, breast	substrate of ABCB1, ABCC1, ABCG2, CYP2E1	inhibitor of ABCB1, ABCC1, CYP1B1, CYP3A4, inducer of ABCB1.	Overexpression of ABC transporters	[64]
	Arsenic derivative		DNA / Unclear	Arsenic trioxide	Leukemia (APL)	Not detoxifyed, accumulates in liver, kidney, heart, lung, hair and nails	AKT1, CCND1, CDKN1A, HDAC1, IKBKB, JUN, MAPK1, MAPK3, PML, TXNRD1	Mutations in PML gene	[65–69]
	Camptothecins / Topoisomerase I inhibitors		DNA, TOP1MT	Irinotecan	CRC, pancreas (liposomal), ovary, esophageal, Ewing's sarcoma, glioblastoma, NSCLC, SCLC, gastric, rhabdomyosarcomas, cervical	substrate of ABCB1, ABCC1, ABCC2, ABCC10, ABCB11, ABCG2, CYP3A4, CYP3A5, CYP3A7, RABP1.	inhibitor of ABCB1, ABCC1, ABCC2, ABCC3, ABCC10, SLC22A3, SLCO1B1 and SLCO1B3	Low carboxylesterase expression (decreased intracelular activation), increased metabolic detox to aminopentanecarboxylic acid (APC), overexpression of ABC transporters, TOPO I downregulation, TOPO I mutations, hyperactivity of NF-κB and DNA repair pathways	[3,70,71]

SUPPLEMENTARY TABLE 1. SUMMARY OF FDA-APPROVED ANTICANCER CYTOTOXIC DRUGS AT MAY 2019 (CONTINUATION)

Classification			Targets	Name	Indication	Detox pathway	Known off-targets	Resistance mechanisms	References
Natural products and semi-synthetic	Cytotoxic Antibiotics	Camptothecins / Topoisomerase I inhibitors	DNA, TOP1, TOP1MT	Topotecan	Ovarian, SCLC, cervical	Substrate of ABCB1, ABCG2, SLC47A1, SLC47A2.	poly(ADP-ribose) polymerase-1 (Daniel et al., 2009); inhibitor of CYP3A4, SLC47A1; inducer of CYP3A4.	Overexpression of ABCB1 and ABCG2, overexpression of aldehyde dehydrogenase-1A1 (ALDH1A1), TOPO I downregulation	[72–74]
		Chromomycin	DNA, TOP2A, TOP2B	Dactinomycin	Sarcoma, Gestational trophoblastic neoplasia, Testicular, Kidney	ABCB1, ABCC1, ABCC6, ABCG2, SLC22A5	Inhibitor of ABCB1	Overexpression of ABC transporters	[3,75]
		DDT/DDD analogue	CYP11B1	Mitotane	Adrenocortical carcinoma	Unclear	AR, ESR1, FDX1, PGR. Inducer of CYP3A4.	Unclear	[76,77]
		Epipodophyllotoxins / Topoisomerase II inhibitos	TOP2A, TOP2B	Etoposide	Testicular, ovarian, lung, acute myeloid leukemia, lymphomas and sarcomas	Substrate of ABCB1, ABCC1, ABCC2, ABCC3, ABCC6, ABCC10, ABCG2, CYP1A2, CYP2E1, CYP3A4, CYP3A5, GSTP1, GSTT1, PTGS1, PTGS2, UGT1A1	Inhibitor of ABCB1, ABCC1, ABCC2, ABCC3, ABCC6, ABCC10, CYP2C8, inducer of CYP3A4, CYP3A5	Overexpression of ABC transporters, mutations in TP53, overexpression of 5'-tyrosyl DNA phosphodiesterase 2 (TDP2) (in vitro)	[3,78,79]
		Glycopeptide	DNA, LIG1, LIG3	Bleomycin	Head and neck, Lymphoma, Penile, Cervical, Vulvar, Testicular	Bleomycin hydrolase (evitar abreviaturas desnecessárias)	Unclear	Increased detox (Activity of bleomycin hydrolase, bleomycin N-acetylating enzymes and bleomycin-binding proteins)	[3,80]
		Isoquinoline	DNA	Trabectedin	Soft tissue sarcomas (Liposarcoma and leiomyosarcoma)	CYP2C9, CYP2C19, CYP2D6, CYP2E1, CYP3A4, PTGS1	Unclear	Unclear	[81]

SUPPLEMENTARY TABLE 1. SUMMARY OF FDA-APPROVED ANTICANCER CYTOTOXIC DRUGS AT MAY 2019 (CONTINUATION)

Data curated from Drugbank (www.drugbank.ca) and SuperCYP/Transformer databases (bioinformatics.charite.de/transformer/).

Abbreviations: ATIC - 5-Aminoimidazole-4-Carboxamide Ribonucleotide, SOD1 - Superoxide dismutase [Cu-Zn], A2M - Alpha-2-macroglobulin, ABC - ATP-binding cassette, ABCC1 - Multidrug resistance-associated protein 1, ADA - Adenosine deaminase, ATOX1 - Copper transport protein ATOX1, ATP7A - Copper-transporting ATPase 1, BCHE - Cholinesterase, CDA - Cytidine deaminase, CES1 - liver carboxylesterase 1, CHD1 - Chromodomain-helicase-DNA-binding protein 1, CYP - Cytochrome P450, DCK - Deoxycytidine kinase, DCK - Deoxycytidine kinase, DCTD - deoxycytidylate deaminase, DHFR - Dihydrofolate reductase, DNMT1 - DNA methyltransferase, DPYD - Dihydropyrimidine dehydrogenase [NADP(+)], ENGase - Beta-n-acetylglucosaminidase, FPGS - Folylpolyglutamate synthase, mitochondrial, GART - glycinamide ribonucleotide formyltransferase, GGH - Gamma-glutamyl hydrolase, GSH - Glutathione, GSR - Glutathione reductase, mitochondrial, GSTM1 - Glutathione S-transferase Mu 1, GSTP1 - Glutathione S-transferase P, GSTP1 - Glutathione S-transferase P, GSTT1 - Glutathione transferase activity, HPRT1 - Hypoxanthine-guanine phosphoribosyltransferase 1, MAOA - Amine oxidase [flavin-containing] A, MAOB - Amine oxidase [flavin-containing] B, MGMT - O6 - methylguanine DNA methyltransferase, MPG - DNA-3-methyladenine glycosylase, MPO - Myeloperoxidase, MT1A - Metallothionein-1A, nat - Arylamine N-acetyltransferase, NER - nucleotide excision repair, NQO1 - NAD(P)H dehydrogenase [quinone] 1, NR1I2 - Nuclear receptor subfamily 1 group I member 2, NT5C2 - 5′-nucleotidase catalytic enzyme, PGD - 6-phosphogluconate dehydrogenase, decarboxylating, OPRTase - Orotate phosphoribosyltransferase, PLA2G4A - Cytosolic phospholipase A2, PNP - Purine nucleoside phosphorylase, POLA2 - DNA polymerase alpha subunit B, POR - NADPH--cytochrome P450 reductase, PTGS1 - Prostaglandin G/H synthase 1, RFC - reduced folate carrier, SLC22A3 - Solute carrier family 22 member 3, ST - Serotransferrin, STMN4 - Stathmin-4, THS - Thymidylate synthase, TOP1MT - DNA topoisomerase I, mitochondrial, TOP2A - DNA topoisomerase 2-alpha, TOP2B - DNA topoisomerase 2-beta, TPMT - Thiopurine S-methyltransferase, TXNRD1 - Thioredoxin reductase 1, cytoplasmic, TYMP - thymidine phosphorylase, TYMS - thymidylate synthase, UGT - UDP-glucuronosyltransferase, UGT1A1 - UDP-glucuronosyltransferase 1-1, XDH - Xanthine dehydrogenase/oxidase, CRC - Colorectal cancer, ALL - Acute Lymphoblastic leukemia, CML - Chronic Lymphoblastic leukemia, SCLC - Small cell lung cancer, NSCLC - Non small cell lung cancer.

SUPPLEMENTARY TABLE 2. SUMMARY OF FDA-APPROVED ANTICANCER TARGETED THERAPIES AT MAY 2019

**Classification**		Targets	Name	Indications	Detox pathway	Known off-targets	Resistance mechanisms	References
ANTIMETABOLITE	Antifolate analogue	DHFR, TYMS	Pralatrexate	T-cell lymphoma	FPGS	Substrate of SLC19A1, Proton-coupled folate transporter (PCFT)	Reduced expression or drug affinity for RFC, increased levels of folic acid	[82,83]
	Enzime	Asparagine (protein synthesis inhibition)	Erwinaze (L-asparaginase)	ALL	Unclear	Glutamine; inhibitor of SERPINA7	Unclear	[84]
CYTOSKELETAL DISRUPTORS	Taxane	TUBA4A, TUBB1	Cabazitaxel	Prostate	Substrate of ABCB1, CYP3A4, CYP3A5, CYP2C8	Inhibitor of ABCB1, ABCG2, SLCO1B1, SLCO1B3	Overexpression of ABCB1, overexpression of TUBB3, downregulation of BRCA1 expression, upregulation of vimentin and downregulation of E-cadherin, reduced expression of antiapoptotic regulators (BCL2 and MCL1), downregulation of IAPs	[85,86]
ENDOCRINE THERAPY	Androgen receptor	AR	Apalutamide	Prostate	CYP2C8, CYP3A4	Inducer of CYP3A4, CYP2C19, CYP2C9, ABCG2; inhibitor of SLC22A2, SLC22A8, SLC47A2; antagonist of GABA-A receptor (low micromolar affinity (IC50=3.0uM); inhibitor of ABCB1, SLCO1B1	*AR* amplification, *AR* point mutations, altered intratumoral androgen biosynthesis, glucocorticoid receptor activation, immune-mediated resistance, autophagy induction and neuroendocrine differentiation.	[87,88]
		AR	Enzalutamide	Prostate	CYP2C8, CYP2C9, CYP3A4, CYP3A5	Inhibitor of ABCB1, CYP2C8, CYP2B6; inducer of CYP3A4, CYP2C9, CYP2C19, CYP2D6.		[87,88]

SUPPLEMENTARY TABLE 2. SUMMARY OF FDA-APPROVED ANTICANCER TARGETED THERAPIES AT MAY 2019 (CONTINUATION)

Classification		**Targets**	**Name**	**Indications**	**Detox pathway**	**Known off-targets**	**Resistance mechanisms**	**References**
ENDOCRINE THERAPY	Antiestrogens	ESR1	Fulvestrant	Breast	Substrate of CYP3A4, UGT1A1	Unclear	Overexpression of EGFR, HER2, and NRG2; enhanced HER3 activation; enhanced ERK, WNT/β-catenin and Notch pathways activation	[89,90]
		ESR1, ESR2, ESRRG	Tamoxifen	Breast	ABCG2, ABCC2, CYP2D6, CYP2B6 (high tamoxifen concentrations), CYP2C9, CYP3A4, CYP3A5, CYP3A7, CYP2C19, CYP1A1, CYP2A6, CYP2E1, UGT1A10, SULT1A1, FMO1, FMO3	*AR, NR1I2, SHBG; inhibitor of CYP2D6, CYP2C9, CYP2B6, CYP1B1, CYP2C8, CES1, CYP19A1, ABCB11, (EBP, PKC, KCNH2); inhibitor and inducer of CYP3A4, ABCB1; inducer of SERPINA7*	Upregulation of NF-κB; overexpression of XBP1, IRF1 and NPM1; enhanced RAS-MAPK signaling; loss of estrogen receptor (ESR1) and progesterone receptor (PGR) expression, mutations in *ESR1* and spliced variant expression; exogenous estrogenic exposures	[91,92]
		ESR1	Toremifene	Breast	ABCB1, CYP3A4, CYP1A1	SHBG; inhibitor of ABCB1		[91–93]
	Aromatase	CYP17A1	Abiraterone	Prostate	ALB, ORM1, CYP3A4, SULT2A1	Inhibitor of CYP2D6, CYP2C8, CYP1A2, CYP2C19, CYP2C9, ABCC1	Upregulation of CYP17A1; CYP17A1-independent AR activation; *AR* amplification, *AR* splice variants that confer ligand-independent AR transactivation, mutations in *AR*, NR3C1 (glucocorticoid receptor) overexpression, autophagy induction	[94,95]
		CYP19A1	Anastrozole	Breast	N-dealkylation, hydroxylation, and glucuronidation to inactive metabolites.	Inhibitor of CYP3A4, CYP1A2 (in vitro), CYP2C9 (not clinically relevant)	Loss of ESR1 expression; mutations, amplification or translocation of *ESR1*; aberrant expression or mutation of ER co-regulators; mutation of *TP53*, *MDM2* amplification, increased levels of BCL-2 and survivin, increased levels of telomerase; loss of *RB1, CDKN2A* and *CDKN2C*; amplification of *CCND1*; enhanced EMT and CSC phenotype: Notch, Hedgehog, WNT and TWIST1 overactivation; enhanced activation of PI3K-AKT-mTOR and MAPK pathway; overexpression, mutation or amplification of *ERBB2/*HER2, *IGF1R* and *EGFR*; increased levels of fibronectin and collagen	[96,97]

SUPPLEMENTARY TABLE 2. SUMMARY OF FDA-APPROVED ANTICANCER TARGETED THERAPIES AT MAY 2019 (CONTINUATION)

Classification		Targets	Name	Indications	Detox pathway	Known off-targets	Resistance mechanisms	References
ENDOCRINE THERAPY	Aromatase	CYP19A1	Exemestane	Breast	CYP3A4	Unclear	Loss of ESR1 expression; mutations, amplification or translocation of *ESR1*; aberrant expression or mutation of ER co-regulators; mutation of *TP53*, *MDM2* amplification, increased levels of BCL-2 and survivin, increased levels of telomerase; loss of *RB1, CDKN2A* and *CDKN2C*; amplification of *CCND1*; enhanced EMT and CSC phenotype: Notch, Hedgehog, WNT and TWIST1 overactivation; enhanced activation of PI3K-AKT-mTOR and MAPK pathway; overexpression, mutation or amplification of *ERBB2*/HER2, *IGF1R* and *EGFR*; increased levels of fibronectin and collagen	[96,97]
		CYP19A1	Letrozole	Breast	CYP3A4, CYP2A6	Inhibitor of CYP2A6		[96,97]
HEDGEHOG PATHWAY INHIBITOR	Hedgehog Pathway	SMO	Glasdegib	AML	CYP3A4, CYP2C8, UGT1A9, ALB, ABCB1, ABCG2; inhibitor of SLC47A1, SLC47A2	Inhibitor of ABCB1, ABCG2, SLC47A1, SLC47A2	Mutations in *SMO;* upregulation of GLI2 and other downstream effectors of Hh/GLI signaling, *GLI3* silencing, enhanced activation of RAF-MEK-MAPK, PI3K-AKT and IGF signaling	[98]
		SMO	Sonidegib	BCC	CYP3A4	Unclear	Mutation in *SMO* and *TP53*, *SUFU* deletion and *MYCN* amplification; upregulation of GLI2 and other downstream effectors of Hh/GLI signaling; enhanced activation of RAF-MEK-MAPK, PI3K-AKT and IGF signaling	[98,99]
		SMO	Vismodegib	Basal cell carcinoma	ABCB1, CYP2C9, CYP3A4	ABCG2, CYP2C9, CYP2C8, CYP2C19	Mutation in *SMO*, upregulation of GLI2, activation of oncogenic signaling pathways (RAF-MEK-ERK cascade, PI3K-AKT signaling, IGF signaling), upregulation of atypical protein kinase C ι/λ (aPKCι/λ), histone deacetylases (HDAC1/HDAC2) and DYRK1B	[98,99]

SUPPLEMENTARY TABLE 2. SUMMARY OF FDA-APPROVED ANTICANCER TARGETED THERAPIES AT MAY 2019 (CONTINUATION)

Classification		Targets	Name	Indications	Detox pathway	Known off-targets	Resistance mechanisms	References
HISTONE DEACETYLASE INHIBITORS	Histone Deacetylases	HDAC1 to 11 (Protein group)	Belinostat	PTCL	ABCB1, UGT1A1	Inhibitor of CYP2C8, UGT1A1, CYP2C9; inducer of CYP1A2	Altered DNA repair activity; abnormalities in the regulation of cell cycle and mitotic chromosomes; JAK and STAT signaling; enhanced RAR signaling (e.g., overexpression of RARα); autophagy; reduction or loss of HDAC1 expression.	[100,101]
			Panobinostat	Multiple myeloma	ABCB1, CYP3A4	Inhibitor of CYP2D6	Mutations in *TP53*; altered DNA repair activity; abnormalities in the regulation of cell cycle and mitotic chromosomes; ROS and redox pathways (high levels of thioredoxin); enhanced BCL-2 anti-apoptotic signaling (e.g., overexpression of BCL-2); constitutive NF-kB activation; JAK and STAT signaling; autophagy; endoplasmic reticulum stress-related signaling	[100,102]
		HDAC1, HDAC2, HDAC4, HDAC6,	Romidepsin	CTCL or/and PTCL	ABCB1, SLCO1B3, CYP3A4, CYP3A5, CYP1A1, CYP2B6, CYP2C19	ABCC1	Sustained expression of CHEK1, altered DNA repair activity; abnormalities in the regulation of cell cycle and mitotic chromosomes; ROS and redox pathways (high levels of thioredoxin); enhanced BCL-2 anti-apoptotic signaling (e.g., overexpression of BCL-2); increased MDR1 expression; enhanced JAK and STAT signaling; autophagy	[100]
		HDAC1, HDAC2, HDAC3, HDAC6, HDAC8,	Vorinostat	T-cell lymphoma	Glucuronidation and hydrolysis followed by β-oxidation	acuC1	Sustained expression of CHEK1, altered DNA repair activity; abnormalities in the regulation of cell cycle and mitotic chromosomes; ROS and redox pathways (high levels of thioredoxin, superoxide dismutase 2, and glutathione reductase); enhanced BCL-2 anti-apoptotic signaling (e.g., overexpression of BCL-2); constitutive NF-kB activation; aberrant JAK and STAT signaling; Retinoic acid signaling; autophagy; endoplasmic reticulum stress-related signaling	[100]

SUPPLEMENTARY TABLE 2. SUMMARY OF FDA-APPROVED ANTICANCER TARGETED THERAPIES AT MAY 2019 (CONTINUATION)

Classification		Targets	Name	Indications	Detox pathway	Known off-targets	Resistance mechanisms	References
KINASE INHIBITORS	ALK inhibitors	ALK	Alectinib	NSCLC	Unclear	Inhibitor of ABCB1, ABCG2	Unclear	[103]
		ALK	Brigatinib	NSCLC	CYP2C8, CYP3A4, ABCB5, ABCG2	Inhibitor and inducer of CYP3A; inducer CYP2B6; inhibitor of ROS1, TYK1, FER, FPS, TRKA, TRKB, TRKC, FAK, FAK2, ACK	Unclear	[104]
		ALK	Ceritinib	NSCLC	CYP3A4, ABCB1	Inhibitor of CYP2C9, CYP3A4	Mutations in ALK tyrosine kinase domain; EMT phenotype; mechanism of “off target” resistance in the bypass of signaling tracks	[105,106]
		ALK	Crizotinib	NSCLC	CYP3A4, CYP3A5, ABCB1.	Inhibitor of ABCB1, CYP2B6, CYP3A4, CYP3A5, CYP3A, MET, RON	Mutations in ALK tyrosine kinase domain and *ALK* amplification, activation of EGFR-, IGF-1R-, or KIT-mediated signaling pathways	[107]
		ALK	Lorlatinib	NSCLC	CYP3A, CYP3A5, CYP2C8, CYP2C19, UGT1A3	Inhibitor and inducer of CYP3A; inducer CYP2B6; ROS1, TYK1, FER, FPS, TRKA, TRKB, TRKC, FAK, FAK2, ACK	Unclear	[108]

SUPPLEMENTARY TABLE 2. SUMMARY OF FDA-APPROVED ANTICANCER TARGETED THERAPIES AT MAY 2019 (CONTINUATION)

Classification		Targets	Name	Indications	Detox pathway	Known off-targets	Resistance mechanisms	References
KINASE INHIBITORS	Bcr-Abl inhibitors	BCR, ABL1	Bosutinib	CML	CYP3A4, ABCB1	LYN; inhibitor of CYP3A4, ABCB1, CYP2C8, HCK, SRC, CDK2, MAP2K1, MAP2K2, MAP3K2, CAMK2G	*BCR-ABL1* fusion gene mutations in the kinase domain, increased BCR-ABL1 expression; upregulation of CYP3A4	[109]
		ABL1, ABL2	Dasatinib	CML, ALL	CYP1A1, CYP1A2, CYP1B1, CYP3A5, FMO3, CYP3A4, ABCB1, ABCG2;	Antagonist EPHA2, KIT, PDGFRB; inhibitor of STAT5B; YES1; multitarget BTK, FYN, ABL2; NR4A3, CSK, EPHA5, EPHB4, FGR, FRK, HSPA8, LYN, ZAK, MAPK14, PPAT; inhibitor of CYP3A4, ABCB1, ABCG2	*BCR-ABL1* fusion gene mutations, increased BCR-ABL1 expression; transporter-mediated TKI efflux (ABCB1, ABCG2); upregulation of CYP3A4	[109]
		BCR/ABL fusion protein isoform X9	Imatinib	CML, ALL, GIST, CEL	CYP3A4, CYP3A5, CYP3A7, CYP2C9, CYP2D6, ABCB1, ABCG2, ALB, ORM1, SLC22A1, ABCA3, ABCB11, CYP1A2, CYP2C19, PTGS1, CYP2C8;	Antagonist and multitarget of KIT; antagonist of NTRK1, CSF1R, PDGFRA, DDR1, PDGFRB; inhibitor of CYP3A4, CYP3A5, CYP3A7, CYP2C9, CYP2D6, ABCB1, ABCG2, SLC22A2, ABL1, RET	*BCR-ABL1* fusion gene mutations, increased BCR-ABL1 expression; transporter-mediated TKI efflux (ABCB1, ABCG2, OCT-1); upregulation of CYP3A4; STAT3 activation, activation of the PI3K/AKT/mTOR pathway, increased RAF/MEK/ERK pathway activity, nucleocytoplasmic transport (XPO1 and RAN)	[109]
		ABL1, ABL2	Nilotinib	CML	CYP3A4, ABCB1, ABCG2	Antagonist of KIT; inhibitor of PDGFRA, PDGFRB, CYP3A4, ABCB1, ABCG2, CYP2C9, CYP2D6, UGT1A1, SLCO1B1; inhibitor and inducer of CYP2C8; inducer of CYP2B6	*BCR-ABL1* fusion gene mutations, increased BCR-ABL1 expression; transporter-mediated TKI efflux (ABCB1, ABCG2); upregulation of CYP3A4; BCR-ABL1 independent resistance: STAT3 activation, activation of the PI3K/AKT/mTOR pathway, increased RAF/MEK/ERK pathway activity and EZH2 overexpression	[109]
		BCR, ABL1	Ponatinib	CML, ALL	CYP3A4, CYP2D6, CYP3A5, CYP2C8, ABCB1, ABCG2	Inhibitor of KIT, RET, TEK FLT-3, FGFR1, FGFR2, FGFR3, FGFR4, LCK, SRC, LYN, KDR, PDGFRA, VEGFR, EPH, CYP2C8, ABCB1, ABCG2	BCR-ABL1 dependent resistance: BCR-ABL1 fusion gene mutations, increased BCR-ABL1 expression, transporter-mediated TKI efflux (OCT-1); upregulation of CYP3A4; BCR-ABL1 independent resistance: STAT3 activation, activation of the PI3K/AKT/mTOR pathway	[109]

SUPPLEMENTARY TABLE 2. SUMMARY OF FDA-APPROVED ANTICANCER TARGETED THERAPIES AT MAY 2019 (CONTINUATION)

Classification		Targets	Name	Indications	Detox pathway	Known off-targets	Resistance mechanisms	References
KINASE INHIBITORS	CDKs	CDK4, CDK6	Abemaciclib	Breast	ABCB1, ABCG2, CYP3A4, SLC47A1	Inhibitor of ABCB1, ABCG2, OTC, SLC47A1, SLC47A2; binder ALB, alpha1-acid glycoprotein	*CDKN2A, CDK6*, *CCNE1/2*, *CDK2* amplification; activation of the FGFR pathway, activation of the PI3K/AKT/mTOR pathway, loss of ER or PR expression, autophagy activation	[110]
		CDK4, CDK6	Palbociclib	Breast	CYP3A4, ABCB1, ABG2, Sulfotransferase 2A1	Inhibitor of CYP3A4, ABCB1, ABCG2, SLC22A1; binder ALB;	Loss of *RB1* and *CCND1*; *CDKN2A*, *CCNE1/2, CDK2*, *E2F2*, *CDK4* amplification; CDK7, MDM2 overexpression; HDAC activation and loss of *FZR1, CDKN1A* (p21) and *CDKN1B* (p27); activation of the FGFR pathway, activation of the PI3K/AKT/mTOR pathway, loss of *ESR1* or *PGR* expression; autophagy activation	[111]
		CDK4, CDK6	Ribociclib	Breast	CYP3A4	Inhibitor of CYP3A4	*CDKN2A* amplification; sustained expression of PDK1*; CDK2* and *CDK4* amplification; activation of the FGFR pathway, activation of the PI3K/AKT/mTOR pathway, loss of *ESR1* or *PGR* expression; autophagy activation	[112]
	FGFR	FGFR1, FGFR2, FGFR3, FGFR4	Erdafitinib	Urothelial carcinoma	ABCB1, SCLC22A1, CYP2C9, CYP3A4,	Substrate of RET, CSF1R, PDGFRA, PDGFRB, KIT, KDR; inhibitor of ABCB1, SCLC22A1	Compensatory signaling or mutations in the FGFR receptors; activation of STAT3; overexpression of RACK1	[113,114]
	PI3K	PIK3CA	Alpelisib	Breast cancer	ABCB1, ABCG2, CYP3A4	Inducer of CYP2C9, inhibitor of CYP2C8, CYP2C19	FGFR1 overexpression and activation of ERK signaling	[115]
		PIK3CA, PIK3CD	Copanlisib	FL	CYP3A4, CYP3A5, CYP3A7, CYP3A43, ALB, ABCB1, ABCG2	Inhibitor of SLC47A2	Unclear	[116]

SUPPLEMENTARY TABLE 2. SUMMARY OF FDA-APPROVED ANTICANCER TARGETED THERAPIES AT MAY 2019 (CONTINUATION)

Classification		Targets	Name	Indications	Detox pathway	Known off-targets	Resistance mechanisms	References
KINASE INHIBITORS	PI3K	PIK3CG, PIK3CD	Duvelisib	CLL, SLL	CYP3A4, ABCB1, ABCG2	Unclear	mTOR-dependent reactivation of AKT activity, enhanced PI3Kα activation and epigenetic reprogramming	[117,118]
		PIK3CD	Idelalisib	CLL, FL, SLL	AOX1, UDP-glucuronosyltransferase 1A4, ABCG2, CYP3A4, CYP3A5, CYP3A43, CYP3A7, ABCB1	Inhibitor of UDP-glucuronosyltransferase 1A1, CYP2C8, CYP2C19, SLCO1B1, SLCO1B3, CYP3A5, CYP3A43, CYP3A7, ABCB1; inducer of CYP2B6; inhibitor and inducer of CYP3A4	*PI3KCA* gain-of-function mutations; compensatory activation of SFK and WNT pathways; *PTEN* loss; upregulation of PI3K-gamma, activation of the PI3K and MAPK pathway	[119]
	RAF (BRAF)	BRAF V600E, RAF1	Dabrafenib	Melanoma	CYP3A4, CYP2C8, ABCG2, ABCB1	Antagonist and inhibitor of SIK1, NEK11, LIMK1; inducer of CYP3A4, CYP2B6, CYP2C9; inhibitor CYP2C8, ABCG2, SLCO1B1, SLCO1B3, SLC22A6, SLC22A8	Increased MAPK pathway reactivation (*BRAF* amplification, mutations in *MEK1/2*); hyperactivation of AKT; *PTEN* loss	[120]
		BRAF (WT, V600E and CRAF)	Encorafenib	Melanoma	CYP3A4, CYP2C19, CYP2D6	Inhibitor of CCND1; binder JNK1, JNK2, JNK3, LIMK1, LIMK2, MEK4, STK36	RAF dimerization, increased MAPK pathway reactivation; ERK reactivation upstream of MEK or enhanced survival signaling (*BRAF* V600 amplification, alternative splicing of *BRAF* V600E mRNA, activating *NRAS* and *KRAS* mutations)	[121,122]
		BRAF V600E	Vemurafenib	Melanoma	CYP3A4, ALB, ORM1, ABCC1, ABCG2	Inhibitor of CYP1A2, CYP2D6, CYP2C9, CYP2C8, ABCC1, ABCG2; inducer of CYP3A4, CYP2B6;	ERK reactivation upstream of MEK or enhanced survival signaling (*BRAF* V600 amplification, alternative splicing of *BRAF* V600E mRNA, activating *NRAS* and *MEK1* mutations, overexpression of NR2F1); enhanced PI3K pathway (mutations in *PI3KCA* or *AKT1*) and increased MITF levels; *CCND1* amplification; *PTEN* loss; induction of autophagy	[121,123]

SUPPLEMENTARY TABLE 2. SUMMARY OF FDA-APPROVED ANTICANCER TARGETED THERAPIES AT MAY 2019 (CONTINUATION)

Classification		Targets	Name	Indications	Detox pathway	Known off-targets	Resistance mechanisms	References
MONOCLONAL ANTIBODY	Antiangiogenic	VEGF	Bevacizumab	CRC, Breast, GBM	mAB multiple clearance pathways (e.g. TMDD, ADA, proteolysis)	FCGR3B, C1R, C1QA, C1QB, C1QC, FCGR3A, FCGR1A, FCGR2A, FCGR2B, FCGR2C	Upregulation of DLL4-Notch signaling, STAT3 and c-MET; enhanced EMT phenotype; increased levels of alternate VEGF ligands (e.g., PIGF, VEGF-D)	[124–126]
		VEGFR2	Ramucirumab	Gastric or gastro-esophageal junction	Not Available	Unclear	Lack of activity with other VEGFR e.g. VEGFR1 and 3; increased VEGF-D levels	[127,128]
	CCR4	CXCR4	Mogamulizumab	Mycosis fungoides (MF) or Sézary syndrome (SS)	mAB multiple clearance pathways (e.g. TMDD, ADA, proteolysis)	Unclear	Unclear	[129]
	CD38	CD38	Daratumumab	Multiple myeloma	mAB multiple clearance pathways (e.g. TMDD, ADA, proteolysis)	Unclear	Reduced cell surface expression levels of the target antigen (CD38) and high levels of complement inhibitors (CD55 and CD59); upregulation of anti-apoptotic molecules (e.g., survivin)	[130]
	EGFR/HER	EGFR	Cetuximab	CRC	mAB multiple clearance pathways (e.g. TMDD, ADA, proteolysis)	FCGR3B, C1R, C1QA, C1QB, C1QC, FCGR3A, C1S, FCGR1A, FCGR2A, FCGR2B, FCGR2C	Reduced *EGFR* copy number, altered expression of EGFR ligands (e.g., amphiregulin and epiregulin), nuclear translocation of EGFR; aberrant downstream EGFR signaling: activating *KRAS* mutations, mutations in *BRAF,* deregulated PIK3CA/PTEN signaling (*PIK3CA* mutations, *PTEN* inactivation), *ERBB2* and c-*MET* amplifications; AXL overexpression	[131–133]
		EGFR	Necitumumab	NSCLC	mAB multiple clearance pathways (e.g. TMDD, ADA, proteolysis)	Unclear	Unclear	[131,132,134]
		EGFR	Panitumumab	CRC	mAB multiple clearance pathways (e.g. TMDD, ADA, proteolysis)	Unclear	Reduced *EGFR* copy number, altered expression of EGFR ligands (e.g., amphiregulin and epiregulin); aberrant downstream EGFR signaling: activating *KRAS* mutations, mutations in *BRAF*, deregulated PIK3CA/PTEN signaling (*PIK3CA* mutations, *PTEN* inactivation)	[131,132,135]

SUPPLEMENTARY TABLE 2. SUMMARY OF FDA-APPROVED ANTICANCER TARGETED THERAPIES AT MAY 2019 (CONTINUATION)

Classification		Targets	Name	Indications	Detox pathway	Known off-targets	Resistance mechanisms	References
MONOCLONAL ANTIBODY	EGFR/HER	HER-2	Pertuzumab	Breast	mAB multiple clearance pathways (e.g. TMDD, ADA, proteolysis)	Unclear	Formation of HER3/EGFR heterodimers and subsequent phosphorylation of AKT and ERK1/2, reduced miR-150 expression (negative PI3K-AKT pathway regulator)	[136]
		HER-2	Trastuzumab	Breast	mAB multiple clearance pathways (e.g. TMDD, ADA, proteolysis)	Inhibitor of CYP19A1; EGFR, C1R, C1QA, C1QB, C1QC, C1S, FCGR1A, FCGR2A, FCGR2B, FCGR2C, FCGR3B, FCGR3A	Hindered access to HER2 (expression of truncated variants, epitope masking by mucin 4 or CD44/hyaluronan polymer complex), upregulation of HER2 downstream signaling (reduction or loss of PTEN, mutations in *PIK3R1, PIK3CA* and *AKT1*, overexpression of MET, all leading to constitutive PI3K/AKT pathway activation), alternate receptor pathway activation (increased IGF-IR signaling, EGFR homodimers and EGFR/HER3 heterodimers), low p27 levels and increased Cdk2 activity	[137]
	Immunotherapy	CD20	Ibritumomab tiuxetan	NHL	mAB multiple clearance pathways (e.g. TMDD, ADA, proteolysis)	FCGR3B, C1R, C1QA, C1QB, C1QC, FCGR3A, C1S, FCGR1A, FCGR2A, FCGR2B, FCGR2C	"Bulky" tumor mass, CD20 downregulation or internalization, NF-κB hyperactivation	[138]
		CD20	Rituximab	NHL,CLL	mAB multiple clearance pathways (e.g. TMDD, ADA, proteolysis)	Unclear	Reduced CD20 expression, reduction of complement-dependent cytotoxicity (CDC) through reduced complement levels and complement activation blockage, polymorphisms in Fc receptors, inactivating mutations in TP53, alterations in apoptotic pathways (NFκB pathway hyperactivation, BCL-2 protein overexpression, downregulation of Bax and Bak)	[139–141]
		CD20	Obinutuzumab	CLL	mAB multiple clearance pathways (e.g. TMDD, ADA, proteolysis)	Unclear	Podocalyxin (PCLP1 - CD34-related sialomucin) expression and lower levels of circulating NK cells	[142,143]
		CD20	Ofatumumab	CLL	mAB multiple clearance pathways (e.g. TMDD, ADA, proteolysis)	Unclear	Reduced MS4A1 expression, reduction of complement-dependent cytotoxicity (CDC) through reduced complement levels and complement activation blockage, inactivating mutations in *TP53*	[139,140]

SUPPLEMENTARY TABLE 2. SUMMARY OF FDA-APPROVED ANTICANCER TARGETED THERAPIES AT MAY 2019 (CONTINUATION)

Classification		Targets	Name	Indications	Detox pathway	Known off-targets	Resistance mechanisms	References
MONOCLONAL ANTIBODY	Immunotherapy	CTLA-4	Ipilimumab	CRC, RCC, melanoma	mAB multiple clearance pathways (e.g. TMDD, ADA, proteolysis)	Unclear	Mutations in the interferon gamma pathway, elevated circulating PD-L1 and CD95 levels, frequent copy number loss, active β-catenin-mediated suppression of CCL4 secretion and poorer anti-tumor immunity (reduced T-cell and DC intratumoral infiltration), higher levels of immune suppressive cell infiltration (Tregs, MDSC, type II macrophages)	[144,145]
		GD2	Dinutuximab	Neuroblastoma	mAB multiple clearance pathways (e.g. TMDD, ADA, proteolysis)	Unclear	Poorer anti-tumor immunity (reduced T-cell and DC intratumoral infiltration), low expression of NK cell-activating ligands (e.g., MHC I), enhanced TGFβ1 secretion, development of anti-dinutuximab antibodies, low GD2 expression	[146]
		CD274 (PD-L1)	Atezolizumab	Urothelial carcinoma	mAB multiple clearance pathways (e.g. TMDD, ADA, proteolysis)	Unclear	Poorer anti-tumor immunity (reduced T-cell and DC intratumoral infiltration), reduced CD274 expression, enrichment of genes associated with EMT phenotype, wound healing, and angiogenesis (IPRES - Innate anti-PD-1 Resistance Signatures),	[147]
		CD274 (PD-L1)	Avelumab	MCC	mAB multiple clearance pathways (e.g. TMDD, ADA, proteolysis)	Unclear	Poorer anti-tumor immunity (reduced T-cell and DC intratumoral infiltration), reduced PD-L1 levels, enrichment of genes associated with EMT phenotype, wound healing, and angiogenesis (IPRES - Innate anti-PD-1 Resistance Signatures)	[148,149]
		CD274 (PD-L1) / CD80	Durvalumab	Urothelial carcinoma	mAB multiple clearance pathways (e.g. TMDD, ADA, proteolysis)	Unclear	Active β-catenin-mediated suppression of CCL4 secretion and poorer anti-tumor immunity (reduced T-cell and DC intratumoral infiltration), reduced PD-L1 levels, enrichment of genes associated with EMT phenotype, wound healing, and angiogenesis (IPRES - Innate anti-PD-1 Resistance Signatures)	[149]
		PDCD1 (PD-1)	Cemiplimab-rwlc	CSCC	mAB multiple clearance pathways (e.g. TMDD, ADA, proteolysis)	Unclear	Common mechanisms to anti-PD-1 therapy: Mutations in the interferon gamma pathway, IPRES phenotype, poor or absent anti-tumor immunity	[150]

SUPPLEMENTARY TABLE 2. SUMMARY OF FDA-APPROVED ANTICANCER TARGETED THERAPIES AT MAY 2019 (CONTINUATION)

Classification		Targets	Name	Indications	Detox pathway	Known off-targets	Resistance mechanisms	References
MONOCLONAL ANTIBODY	Immunotherapy	PDCD1 (PD-1)	Nivolumab	CRC, RCC, melanoma, Hodgkin lymphoma, NSCLC, SCC, urothelial carcinoma, hepatocelullar carcinoma	mAB multiple clearance pathways (e.g. TMDD, ADA, proteolysis)	Unclear	Mutations in the interferon gamma pathway (*JAK1/2* and *IFNGR1/2*), enrichment of genes associated to EMT phenotype, wound healing, and angiogenesis (IPRES - Innate anti-PD-1 Resistance Signatures); poor or absent anti-tumor immunity (absence of antigen-specific T-cells within tumor tissue as well as loss of T cell functionality, MHC downregulation and low neoantigen load), upregulation of immune suppressive cell populations (Tregs, MDSC, type II macrophages)	[144,150]
		PDCD1 (PD-1)	Pembrolizumab	Head and neck, melanoma, gastric or gastroesophageal junction, MIH, MDR, NSCLC, cervical, Hodgkin lymphoma, mediastinal B-cell lymphoma, urothelial carcinoma. nonsquamous NSCLC	mAB multiple clearance pathways (e.g. TMDD, ADA, proteolysis)	Unclear	Biallelic *PTEN* loss, enrichment of genes associated with EMT phenotype, wound healing, and angiogenesis (IPRES - Innate anti-PD-1 Resistance Signatures), mutations in the interferon gamma pathway (*JAK1/2* and *IFNGR1/2*), poor or absent anti-tumor immunity (absence of antigen-specific T-cells within tumor tissue as well as loss of T cell functionality, MHC downregulation and low neoantigen load), constitutive *CD274* expression, high expression of *NT5E*, upregulation of immune suppressive cell populations (Tregs, MDSC, type II macrophages)	[144,150]
		NK cells activator (SLAMF7)	Elotuzumab	Multiple myeloma	mAB multiple clearance pathways (e.g. TMDD, ADA, proteolysis)	Unclear	Soluble forms of SLAMF7, development of anti-elotuzumab neutralizing antibodies	[151]
MONOCLONAL ANTIBODY (MAB) / FUSION PROTEINS	Cytotoxin fusion proteins	**CD19**	Axicabtagene ciloleucel	DLBCL	mAB multiple clearance pathways (e.g. TMDD, ADA, proteolysis)	Unclear	*CD19* loss (mutations, alternative splicing or disrupted CD19 membrane trafficking)	[152]
		**CD19**	Blinatumomab (bi-specific T-cell engagers [BITE]-class CD3/CD19)	ALL	mAB multiple clearance pathways (e.g. TMDD, ADA, proteolysis)	Unclear	*CD19* loss (mutations, alternative splicing or disrupted CD19 membrane trafficking), upregulation of programmed death-ligand 1 (PD-L1) (case report)	[153,154]

SUPPLEMENTARY TABLE 2. SUMMARY OF FDA-APPROVED ANTICANCER TARGETED THERAPIES AT MAY 2019 (CONTINUATION)

Classification		Targets	Name	Indications	Detox pathway	Known off-targets	Resistance mechanisms	References
MONOCLONAL ANTIBODY (MAB) / FUSION PROTEINS	Cytotoxin fusion proteins	L-asparagine	Calaspargase pegol	ALL	mAB multiple clearance pathways (e.g. TMDD, ADA, proteolysis)	Unclear	Upregulation of asparagine synthetase, development of anti-asparaginase antibodies	[155]
		IL-2	Denileukin diftitox	T-cell lymphoma	mAB multiple clearance pathways (e.g. TMDD, ADA, proteolysis)	Unclear	Unclear	[156]
		CD33	Gemtuzumab ozogamicin	AML	mAB multiple clearance pathways (e.g. TMDD, ADA, proteolysis)	FCGR3B, C1R, C1QA, C1QB, C1QC, FCGR3A, C1S, FCGR1A, FCGR2A, FCGR2B, FCGR2C	Overexpression of ABCB1, deficient activation of Bax and Bak, overexpression of antiapoptotic BCL2 and BCL2L1, high CD33 blood load, enhanced PI3K/AKT signaling,	[157,158]
		CD22	Inotuzumab ozogamicin	ALL	mAB multiple clearance pathways (e.g. TMDD, ADA, proteolysis), ABCB1	Unclear	*TET2* and *DNMT3A* mutations, reduced CD22 expression and overexpression of ABCB1	[159,160]
		CD22	Moxetumomab pasudotox-tdfk	HCL	mAB multiple clearance pathways (e.g. TMDD, ADA, proteolysis)	EEF2	Reduced CD22 expression, downregulation of diphthamide biosynthesis protein 1 (DPH1) and 4 (DPH4)	[161–163]
		CD123	Tagraxofusp-erzs	rare leukemia (BPDCN)	mAB multiple clearance pathways (e.g. TMDD, ADA, proteolysis)	ARL2	Reduced IL3RA expression; downregulation of diphthamide biosynthesis protein 1 (DPH1)	[164]
		CD19	Tisagenlecleucel	ALL	mAB multiple clearance pathways (e.g. TMDD, ADA, proteolysis)	Unclear	Mutations in *CD19*, expression of CD19 alternative splicing variants	[153]

SUPPLEMENTARY TABLE 2. SUMMARY OF FDA-APPROVED ANTICANCER TARGETED THERAPIES AT MAY 2019 (CONTINUATION)

Classification		Targets	Name	Indications	Detox pathway	Known off-targets	Resistance mechanisms	References
MONOCLONAL ANTIBODY (MAB) / FUSION PROTEINS	Cytotoxin fusion proteins	EGFR, HER-2	Ado-Trastuzumab Emtansine	Breast	ABCB1, CYP3A4, CYP3A5. mAB multiple clearance pathways (e.g. TMDD, ADA, proteolysis)	Inhibitor CYP3A4.	Decrease in HER2 protein levels, HER2 blockage (e.g., presence of neuregulin), overexpression of ABC transporters (ABCB1, ABCC1); mutations in *PIK3CA, PTEN* deletions, low expression and activity of lysosomal enzymes that degrade the conjugate; altered receptor, apoptotic or other signaling pathways; defects in ADC intracellular trafficking; silencing of cyclin B	[158,165]
		TNFRSF8 (CD30)	Brentuximab vedotin	Hodgkin Lymphoma	CYP3A4, ABCB5. mAB multiple clearance pathways (e.g. TMDD, ADA, proteolysis)	Inhibitor of CYP3A4	CD30 downregulation; resistance to microtubule-disrupting agent monomethyl auristatin E (MMAE); overexpression of ABCB1	[166]
		VEGFA, VEGFB, PGF	Ziv-aflibercept	CRC	mAB multiple clearance pathways (e.g. TMDD, ADA, proteolysis)	Unclear	STAT3 pathway activation and increased levels of proinflammatory mediators (e.g., IL6); upregulation of proangiogenic factors (e.g., IL8); upregulation of alternate VEGFR ligands (e.g., VEGFC, VEGFD, bFGF) and activation of alternative angiogenic pathways	[167]
SYNTHETIC INHIBITORS	mTOR inhibitors	MTOR	Everolimus	Breast, RCC, PNET, TSC, SEGA	CYP3A4	Inhibitor of SLCO1B1, SLCO1B3, SLCO1A2	Mutations in mTOR pathway: enhanced activity of mTORC2, enhanced activation of PI3K/AKT and RAS/RAF/MEK/MAPK pathways; genetic heterogeneity among cancer cells leading to differential mTORC1 activation	[168]
		MTOR	Temsirolimus	RCC	CYP3A4, CYP3A7, CYP3A5, ABCB1	Inhibitor of CYP3A5, ABCB1, CYP2D6	Overactivation of PI3K/AKT, activated STAT3 and ERK pathways, quantitative alterations of integrin α5 and β3 expression, enhanced activity of mTORC2	[169–171]

SUPPLEMENTARY TABLE 2. SUMMARY OF FDA-APPROVED ANTICANCER TARGETED THERAPIES AT MAY 2019 (CONTINUATION)

Classification		Targets	Name	Indications	Detox pathway	Known off-targets	Resistance mechanisms	References
SYNTHETIC INHIBITORS	Noradrenaline transporter	Noradrenaline transporter	Iobenguane	Neuroblastoma, pheochromocytoma	Unclear	Unclear	Unclear	[172]
	PARP inhibitors	PARP-1 and PARP-2	Niraparib	Epithelial ovarian, fallopian tube, or primary peritoneal cancer	CES1A1a, GUSB	Unclear	Loss of *DCLRE1C* (Artemis)	[173]
		PARP-1, PARP-2, PARP-3	Olaparib	Breast, ovarian	CYP3A4, CYP3A5, ABCB1	Inhibitor of CYP3A4, ABCG2; inducer of CYP2B6	Partial restoration of homologous recombination molecular pathway; mutation in *BRCA;* PARP1 and IDO1 overexpression; enhanced activation of ABCB1	[174–176]
		PARP-1, PARP-2, PARP-3	Rucaparib	Ovarian	CYP2D6, ABCB1, ABCG2, CYP1A2, CYP3A4	Inhibitor of CYP2D6, ABCB1, ABCG2, CYP3A5, CYP3A7, CYP3A43, CYP2C19, CYP2C9, CYP2C8, UGT1A1, SLC47A1, SLC47A2, SLC22A1, ABCC4, SLCO1B1, SLCO1B3, SLC22A6, SLC22A8; inhibitor and inducer of CYP1A2; inhibitor and downregulator of CYP3A4	Secondary mutations in *BRCA1/2* restoring RAD51C and RAD51D, NHEJ defects, upregulation of ABC transporters, reconfiguration of the cellular DNA damage, hypomorphic activity of mutant BRCA1 alleles	[177,178]
	BCL-2 inhibitors	BCL-2	Venetoclax	CLL, SLL	CYP3A4, ABCB1	Inhibitor of CYP3A4, ABCB1, ABCG2, SLCO1B1	Mutation in the BH3-binding domain of *BCL2*; increased expression of MCL1 or BCL2L*;* increased autophagy; mutations in *FLT3* or *PTPN11*	[179–181]

SUPPLEMENTARY TABLE 2. SUMMARY OF FDA-APPROVED ANTICANCER TARGETED THERAPIES AT MAY 2019 (CONTINUATION)

Classification		Targets	Name	Indications	Detox pathway	Known off-targets	Resistance mechanisms	References
SYNTHETIC INHIBITORS	Proteasome inhibitors	PSMB1, PSMB5	Bortezomib	Multiple myeloma	CYP3A4, CYP2C19, CYP1A2, CYP2C9, CYP2D6	Inhibitor of CYP3A4, CYP2C19, CYP1A2, CYP2C9	*PSMB5* mutations, overexpression of ABCB1, upregulation of proteasome subunits, upregulation of IL-6, IGF-1, IGF1-R, HSPs, WNT/B-catenin, PI3K/AKT, c-Met, Rad, induced autophagy, expression of MCL1	[182]
		PSMB1, PSMB2, PSMB5, PSMB8, PSMB9, PSMB10	Carfilzomib	Multiple myeloma	ABCB1	Unclear	Upregulation of ABCB1, KLF4-SQSTM1/p62-mediated enhance of autophagy, BCL-2 upregulation, expression of MCL1	[183–185]
		PSMB1, PSMB2, PSMB5	Ixazomib	Multiple myeloma	CYP3A4, CYP1A2, CYP2B6, CYP2C8, CYP2D6, CYP2C19, CYP2C9	Unclear	Mutations in *KRAS*, overexpression of FOXM1	[186,187]
	Retinoic acid receptor inhibitors	RARA, RARB, RARG, RXRA, RXRB, RXRG	Alitretinoin	AIDS-related Kaposi's sarcoma	ABCB1	IGFBP3, PSG5, CYP26C1; agonist of CRABP2, CRABP1	Reduction or loss of expression of retinoic acid receptors (e.g., RARβ2 and RARβ1), mutations in RAR subunits (e.g., RARα)	[188]
		RXRA, RXRB, RXRG	Bexarotene	CTCL	CYP2C9, CYP3A4	Inducer of CYP3A4; inhibitor of CYP2C8	Reduced expression of RXR-α receptor subunit	[189]
		RARA	Isotretinoin	Neuroblastoma	Not Available	Binder ALB	SOX2 expression	[190]

SUPPLEMENTARY TABLE 2. SUMMARY OF FDA-APPROVED ANTICANCER TARGETED THERAPIES AT MAY 2019 (CONTINUATION)

Classification		Targets	Name	Indications	Detox pathway	Known off-targets	Resistance mechanisms	References
SYNTHETIC INHIBITORS	Retinoic acid receptor inhibitors	RARA, RARB, RARG, RXRA, RXRB, RXRG	Tretinoin	APL	CYP3A7, CYP2B6, CYP2C8, CYP2C9, CYP3A4, CYP3A5, CYP2A6, CYP2C18, CYP1A1, CYP4A11, CRABP1, CRABP2; ALB	NR0B1, LCN1, OBP2A, PDK4, CYP26A1, CYP26B1, CYP26C1, HPGDS, ALDH1A1, GPRC5A, ALDH1A2, agonist RARRES1, RBP4, CYP26A1, CYP26B1, CYP26C1	Suppression of RARβ2 and RARβ1 expression, induction of cytoplasmic retinoic acid binding proteins (CRABP) (increased drug sequestration)	[191,192]
	Somatostatin analogues	SSTR2, SSTR5	Lanreotide	Neuroendocrine tumors and acromegaly	Not Available	Inhibitor of CYP3A4	Absence or reduced density of SSTR with high affinity for SA or the heterogeneous expression of SSTR within tumors; mutations in SSTR leading to the absence of functional receptors; and desensitization of SSTR for the uncoupling to the signaling cascade	[193]
		SSTR1, SSTR2, SSTR3, SSTR4, SSTR5	Lutetium dotatate	Endocrine/Neuro endocrine tumors	Does not undergo hepatic metabolism	Unclear		[193]
TYROSINE KINASE INHIBITOR	Bruton tyrosine kinase (BTK)	BTK	Acalabrutinib	MCL	CYP3A	Inhibitor of CYP3A4, CYP3A5	Mutations in *BTK*	[194]
		BTK	Ibrutinib	MCL, CLL, WM	CYP3A4, CYP3A5, CYP2D6, ALB, alpha1-acid glycoprotein	ITK, EGFR, HER-2, HER-4, JAK3, BLK, FGR, FYN, HCK, LCK, LYN, SRC, and YES1	*BTK* and/or *PLCG2* mutations	[195–197]

SUPPLEMENTARY TABLE 2. SUMMARY OF FDA-APPROVED ANTICANCER TARGETED THERAPIES AT MAY 2019 (CONTINUATION)

Classification		Targets	Name	Indications	Detox pathway	Known off-targets	Resistance mechanisms	References
TYROSINE KINASE INHIBITOR	EGFR/HER inhibitors	EGFR, HER-2, HER-3, HER-3	Afatinib	NSCLC	ABCB1, ABCG2	Inhibitor of ABCB1, ABCG2.	Secondary EGFR mutations (T790M), PI3K/AKT, MAPK pathway activation, MET amplification, AXL, HER2, NF-κB, BRCA1, BIM, IGF1R activation, FGFR activation	[198–200]
		EGFR, HER2, HER4	Dacomitinib	NSCLC	ABCB1, ABCG2, CYP3A4, CYP2C9, CYP2D6.	Inhibitor of ABCB1, ABCG2, CYP2D6, UGT1A1, SLC22A1; binder ALB	Secondary EGFR mutations (T790M and C797S); loss of *PI3K/AKT, MAPK, MET, AXL, ERBB2, NFKB1, BRCA1, IGF1R, FGFR, PTEN*	[198,201]
		EGFR	Erlotinib	NSCLC, Pancreas	CYP3A4, CYP2C8, ABCB1, CYP3A5, CYP1A2, CYP1A1, CYP2D6, CYP1B1, ALB, ORM1	Agonist of NR1/2; inhibitor of ABCG2, CYP3A4, CYP2C8, UGT1A1, SLCO2B1.	Reduced *EGFR* copy number, mutations in *EGFR*, aberrant downstream EGFR signaling (*KRAS* mutations, loss of *PTEN*), amplification in *MET*; augmented TGF-β-mediated EMT and increased secretion of IL-6	[131,132,202]
		EGFR	Gefitinib	NSCLC	ABCB1, ABCG2, CYP3A4, CYP2D6, CYP1A1, ALB, ORM1	Inhibitor of CYP3A4, CYP2D6, CYP1A1, ABCB1, ABCG2, CYP2C9, CYP2C19.	Reduced *EGFR* copy number, mutations in *EGFR*, increased EGFR ligand expression (e.g., amphiregulin and epiregulin), overexpression of ABC transporters, amplification in *MET*, aberrant downstream EGFR pathways (*KRAS* mutations, loss of PTEN), overexpression of ABC transporters,	[131,132]
		EGFR, HER-2	Lapatinib	Breast	CYP2C8, CYP3A4, CYP3A5, CYP2C19.	inhibitor of ABCB1, CYP2C8, CYP3A4, TAP1.	Overexpression of ABC transporters, enhanced AXL kinase activity, enhanced PI3K-AKT pathway activation (*PIK3CA* mutations and/or *PTEN* loss), enhanced autophagy, hypoxia-mediated hyperactivation of ERK pathway	[203,204]
		EGFR, HER2, HER4	Neratinib	Breast	CYP3A4, FMO3	Inhibitor of ABCB1	Mutations in *ERBB2*, reactivation of HER3 and enhanced AKT signaling	[204,205]
		EGFR (T790M)	Osimertinib	NSCLC	ABCB1, ABCG2, CYP3A4	Inhibitor of CYP3A4; inducer of CYP1A2, CYP3A4	Mutations in *EGFR*; *EGFR, HER2* and *MET* amplification, amplification in *MET*, aberrant downstream pathways (*KRAS* and *NRAS* mutations, loss of PTEN), MAPK1 and AKT3 overexpression	[131,132,206]

SUPPLEMENTARY TABLE 2. SUMMARY OF FDA-APPROVED ANTICANCER TARGETED THERAPIES AT MAY 2019 (CONTINUATION)

Classification		Targets	Name	Indications	Detox pathway	Known off-targets	Resistance mechanisms	References
TYROSINE KINASE INHIBITOR	IDH inhibitors	IDH2 (R140Q, R172S, R172K)	Enasidenib	AML	CYP1A2, CYP2C8, CYP2C9, CYP2C19, CYP2D6, UGT1A1, ABCG2, CYP2B6, CYP3A4, UGT1A3, UGT1A4, UGT1A9, UGT2B7, UGT2B15	Inhibitor of CYP1A2, CYP2C8, CYP2C9, CYP2C19, CYP2D6, UGT1A1, ABCG2, ABCB1, SLC22A6, SLCO1B1, SLC22A2, SLCO1B3, SLC22A8; inhibitor and inducer of CYP2B6, CYP3A4;	Expression of the Q316E or I319M mutation together with the R140Q mutation (in trans), heterogeneity of mIDH2 subclones, MAPK pathway activation	[207–209]
		IDH1	Ivosidenib	AML	CYP3A4, ABCB1	Inducer of CYP3A4, CYP2B6, CYP2C9, CYP2C8; inhibitor of SLC22A8, ABCB1	IDH dimer-interface mutations in cis, *IDH1* R132C and S280F mutation	[207,210]
	JAK1/2	JAK1, JAK2	Ruxolitinib	Myelofibrosis	CYP3A4	Unclear	Absence or minor reduction in spleen size (primary resistance), spleen regrowth and recurrence of symptoms (secondary resistance), mutations in RUX-binding region, absence of JAK2; mutations in *MPL*, *TET2*, and *SRSF2*, activating R683T, JAK2-V617F/L983F	[211–214]
	MEK inhibitors	MAP3K1, MAP2K2	Binimetinib	Melanoma	UGT1A1, CYP1A2, CYP2C19	IL6, TNF, IL1B	*MYCN* amplification and overexpression, incomplete loss of ERK phosphorylation or minimal effects on MEK phosphorylation	[215,216]
		MAP2K1	Cobimetinib	Melanoma	CYP3A4, ABCB1, SLCO1B1, SLCO1B3	Weak inhibitor SLCO1B1, ABCG2; inhibitor of SLCO1B3	Upregulation of MCL-1 by BRAFV600E	[217]
		MAP2K1, MAP2K2	Trametinib	Melanoma	Via deacetylation or with mono-oxygenation or in combination with glucuronidation biotransformatio n pathways in vitro.	Inhibitor of CYP2C8; inducer of CYP3A4	Changes in the ATXN1L-CIC-ETS transcription factor axis (downstream of MEK), lack of DUSP6, upregulation of MAPK signaling, *PTEN* loss, enhanced HGF/MET signaling, amplifications in *CCND1;* amplified receptor tyrosine kinase (RTK) signaling through PI3K and mTOR, induced cytotoxic autophagy	[218–222]

SUPPLEMENTARY TABLE 2. SUMMARY OF FDA-APPROVED ANTICANCER TARGETED THERAPIES AT MAY 2019 (CONTINUATION)

Classification		Targets	Name	Indications	Detox pathway	Known off-targets	Resistance mechanisms	References
TYROSINE KINASE INHIBITOR	Multi-targeted receptor tyrosine kinase	FLT3, AXL, ALK	Gilteritinib	AML	CYP3A4, ABCB1	Serotonin receptors (protein group); binder ALB; inhibitor of SLC47A1, ABCG2, SLC22A1.	Mutations in *FLT3* (substitutions at D835 in the tyrosine kinase domain (TKD) activation loop and the so-called "gatekeeper" residue F69)	[223–225]
		PKCalpha, VEGFR2, KIT, PDGFR, FLT3	Midostaurin	AML, SM-AHN, MCL, ASM	CYP3A, CYP3A4, CYP2C8, CYP2C9, CYP2C19	Inhibitor and inducer of CYP3A, CYP3A4, CYP2C8, CYP2C9, CYP2C19, CYP1A2; inducer of CYP2B6, CYP3A5, CYP3A7, CYP3A43; inhibitor of CYP2D6, CYP2E1	Mutations in *KRAS* and *TP53*, *FLT3* point mutations	[226–228]
		VEGFR1, VEGFR2, VEGFR3, PDGFRA, PDGFRB, KIT	Pazopanib	RCC, Soft tissue sarcoma	CYP1A2, ABCB1, ABCG2, CYP3A4, CYP2D6, CYP2C8	Inhibitor of FGFR3, ITK, FGF1, SH2B3, SLCO1B1, UGT1A1, CYP3A4, CYP2D6, CYP2C8	Downregulation of DUSP6, overactivation of IGF1 and insulin receptors (IGF1R/InsR), sustained AKT activation, leading to constitutive ERK activation, overexpression of LAMP1/2	[229–231]
		VEGFR1, VEGFR2, VEGFR3, KIT, RET, PDGFRA, PDGFRB, FGFR1, FGFR2, TIE2, DDR2, TrKA, EphA2, RAF-1, BRAF, BRAFV600E, SAPK2, PTK5, ABL1	Regorafenib	CRC, GIST	CYP3A4, UGT1A9, ABCB1	Inhibitor of CYP3A4, UGT1A9, ABCB1, CYP2C8, CYP2C9, CYP2B6, UGT1A1, ABCG2, TEK, NTRK1, MPK11, FRK	Notch-1, HES1 and HEY upregulation, circulating miR-652–3 p and miR-3614–3, enhanced CXCL12/CXCR4 signaling, HER-2 overexpression	[232–235]

SUPPLEMENTARY TABLE 2. SUMMARY OF FDA-APPROVED ANTICANCER TARGETED THERAPIES AT MAY 2019 (CONTINUATION)

Classification		Targets	Name	Indications	Detox pathway	Known off-targets	Resistance mechanisms	References
TYROSINE KINASE INHIBITOR	Multi-targeted receptor tyrosine kinase	RAF1, BRAF, mutant BRAF, PDGFB, VEGFR1, VEGFR2, VEGFR3, FLT3, KIT	Sorafenib	RCC, HCC	CYP3A5, CYP3A7, CYP1A2, RALBP1, CYP3A4, CYP2C8, UGT1A9, ABCG2, ABCB1	Inhibitor of FGFR1, RET, CYP2C9, CYP2B6, CYP2C19, CYP2D6, UGT1A1, ABCC4, ABCC2, SLCO1B1, CYP3A4, CYP2C8, UGT1A9, ABCG2, ABCB1	Upregulation of alternative angiogenic pathways (FGF, PDFG, IL-8), ineffective target inhibition due to reduced drug levels and/or enhanced receptor signaling, upregulation of HIF due to sustained VEGF blockade, upregulation of mTORC2 in response to sustained mTORC1 inhibition mediated by increased PI3K and AKT activity, mutation in F691.	[227,236]
		CSF1R, VEGFR1, VEGFR2, VEGFR3, FLT3, KDR, KIT, PDGFRA, PDGFRB, RET	Sunitinib	RCC, GIST	CYP3A5, CYP3A7, CYP3A4	Inhibitor of ABCC4, ABCB1, ABCC2, ABCG2, CYP3A4	Upregulation of alternative angiogenic pathways (FGF, PDFG, IL-8), ineffective target inhibition due to reduced drug levels and/or enhanced receptor signaling, upregulation of HIF due to sustained VEGF blockade, upregulation of mTORC2 in response to sustained mTORC1 inhibition mediated by increased PI3K and AKT activity, increased lysosomal sequestration leading to increased intracellular lysosomal storage	[231,236,237]
		VEGFR, EGFR, RET	Vandetanib	Thyroid	FMO1, FMO3, CYP3A4	Inhibitor of PTK6, TEK, ABCC1, ABCG2, SLC22A2, binder of ORM1, ALB	S904F mutation in RET kinase domain and p.L881V mutation in the RET kinase domain of KIF5B-RET	[238,239]
	**Tropomyosin receptor kinase (TRK)**	NTRK1, NTRK2, NTRK3	Larotrectinib	Solid tumors with an NTRK gene fusion	CYP3A4	Unclear	NTRK kinase domain mutations: solvent-front NTRK3 G623R or NTRK1 G595R, gatekeeper mutations NTRK1 F589L, xDFG position NTRK1 G667S or NTRK3 G696A	[240]

SUPPLEMENTARY TABLE 2. SUMMARY OF FDA-APPROVED ANTICANCER TARGETED THERAPIES AT MAY 2019 (CONTINUATION)

Classification		Targets	Name	Indications	Detox pathway	Known off-targets	Resistance mechanisms	References
TYROSINE KINASE INHIBITOR	VEGF/VEGFR inhibitors	VEGFR1, VEGFR2, VEGFR3, FGFR1, FGFR2, FGFR3, FGFR4, PDGFRA, KIT, RET	Lenvatinib	Thyroid	CYP3A4, AOX1, ABCB1, ABCG2; inhibitor of CYP2C8, CYP2C9, CYP1A2, CYP2B6, CYP2C19, CYP2D6, SLC22A6, SLC22A8, SLC22A2, SLCO1B1, ABCB11	Inhibitor of CYP2C8, CYP2C9, CYP1A2, CYP2B6, CYP2C19, CYP2D6, SLC22A6, SLC22A8, SLC22A2, SLCO1B1, ABCB11; inhibitor and inducer CYP3A4	HGF pathway, activation of alternative pathways: c-MET, MEK, PI3K-AKT cascade and mTOR, higher level of SDF1α, p.L881V mutation in the RET kinase domain of KIF5B-RET	[239,241–243]
		VEGFR1, VEGFR2, VEGFR3	Axitinib	Pancreas, Thyroid	CYP3A4, CYP3A5, CYP1A2, CYP2C19, UGT1A1, ABCB1, SLCO1B1	Inhibitor of SLCO1B1	Increased glucose metabolism mediated by activated Akt, mutations in BCR-ABL1, upregulation of alternative angiogenic pathways (FGF, PDFG, IL-8), ineffective target inhibition due to reduced drug levels and/or enhanced receptor signaling, upregulation of HIF due to sustained VEGF blockade, upregulation of mTORC2 in response to sustained mTORC1 inhibition mediated by increased PI3K and AKT activity.	[130,236,244,245]
		KDR, MET, RET (VEGFR1, VEGFR2 and VEGFR3, KIT, TRKB, FLT-3, AXL, RET, MET and TIE-2	Cabozantinib	RCC, Thyroid	CYP3A4, CYP2C9	Inhibitor of CYP2C8	Overactivation of integrin, vascular heterogeneity (VEGFR1-positive vessels), overactivation of FGFR1, secretion of PAPPA and IGFBP2, missense RET mutations, *ROS1* mutation	[246]

Data curated from Drugbank (www.drugbank.ca) and SuperCYP/Transformer databases (bioinformatics.charite.de/transformer/).
Abbreviations: ABCB1 - ATP Binding Cassette Subfamily B Member 1, ABCC1 - ATP Binding Cassette Subfamily C Member 1, ABCC2 - ATP Binding Cassette Subfamily C Member 2, ABCG2 - ATP-binding cassette super-family G member 2, ACK - Activated CDC42 kinase , ADN - Deoxyribonucleic acid, AKT - Protein kinase B, ALB - Serum albumin, ALK - Anaplastic lymphoma kinase, ALL - Acute lymphoblastic leukemia, AML - Acute myeloid leukemia, AOX1 - Aldehyde Oxidase 1, AR - Androgen receptor, BCC - Basal Cell Carcinoma, BCL2 - B-cell lymphoma protein 2, BCR - Breakpoint cluster region protein, BCR/ABL - BCR/ABL fusion protein isoform X9, BRCA1 - Breast cancer 1, BTK - Bruton's tyrosine kinase, CAMK2G - Calcium/Calmodulin Dependent Protein Kinase II Gamma, CCND1 - Cyclin D1, CCNE1/2 - Cyclin-E1/E2, CDK2 - Cyclin-dependent kinase 2, CDK6 - Cyclin-dependent kinase 6, CDKN1A - Cyclin Dependent Kinase Inhibitor 1A, CDKN1B - Cyclin Dependent Kinase Inhibitor 1B, CDKN2A - Cyclin-dependent kinase Inhibitor 2A, CDKN2C - Cyclin Dependent Kinase Inhibitor 2C, CEL - Chronic Eosinophilic Leukemia, CES1 - Carboxylesterase 1, CHEK1 - Checkpoint kinase 1, CLL - Chronic lymphocytic leukemia, CML - Chronic myeloid leukemia, CSC - Cancer Stem Cells, CSK - C-terminal Src kinase, CTCL - Cutaneous T cell lymphoma , CYP - Cytochrome P450, DHFR - Dihydrofolate reductase, EBP - Emopamil binding protein, EGFR - Epidermal growth factor receptor, EMT - Epithelial to mesenchymal transition, EPHA - Ephrin type-A receptor , ER - Estrogen Receptor, ERBB2/HER2 - Human Epidermal growth factor Receptor 2, ERK - Extracellular signal–regulated kinase, ESR1 - Estrogen Receptor 1, ESR2 - Estrogen Receptor 2, ESRRG - Estrogen Related Receptor Gamma, EZH2 - Enhancer of zeste homolog 2, FAK - Focal adhesion kinase, FER - Tyrosine-protein kinase Fer, FGFR - Fibroblast growth factor receptor, FGR - Tyrosine-protein kinase Fgr, FL - Relapsed follicular lymphoma, FMO1 - Flavin Containing Dimethylaniline Monoxygenase 1, FMO3 - Flavin Containing Dimethylaniline Monoxygenase 3, FPGS - Folylpolyglutamate synthase, FPS - Farnesyl diphosphate synthase, FRK - Tyrosine-protein kinase FRK (continues on next page)

SUPPLEMENTARY TABLE 2. SUMMARY OF FDA-APPROVED ANTICANCER TARGETED THERAPIES AT MAY 2019 (CONTINUATION)

Abbreviations: FYN - Tyrosine-protein kinase Fyn, FZR1 - Fizzy And Cell Division Cycle 20 Related 1, GABA-A - Type-A γ-aminobutyric, GIST - Gastrointestinal stromal tumors, GLI - Zinc finger protein, HCK - Hematopoietic cell kinase, HDAC - Histone deacetylases, HER3 - Human epidermal growth factor receptor 3, HSPA8 - Heat shock cognate 71 kDa protein, IAPs - Inhibitor of apoptosis proteins, IGF - Insulin-like growth factors, IRF1 - Interferon regulatory factor 1, JAK - Janus kinase, KCNH2 - Potassium Voltage-Gated Channel Subfamily H Member 2, KDR - Vascular endothelial growth factor receptor 2, KIT - Mast/stem cell growth factor receptor Kit, LYN - Tyrosine-protein kinase Lyn, MAPK - Mitogen Activated Protein Kinases, MCL1 - Myeloid cell leukemia 1, MDM2 - Murine Doble Minute 2, MDR1 - Multidrug resistance protein 1, MET - Hepatocyte growth factor receptor, mTOR - Mammalian target of rapamycin, NPM1 - Nucleolar phosphoprotein B23 or numatrin, NR1I2 - Nuclear Receptor Subfamily 1 Group I Member 2, NR3C1 - Nuclear Receptor Subfamily 3 Group C Member 1, NR4A3 - Nuclear Receptor Subfamily 4 Group A Member 3, NRG2 - Neuregulin 2, NSCLC - Non-small-cell lung carcinoma, ORM1 - Orosomucoid 1, PCFT - Proton-coupled folate transporter, PDGFRB - Platelet Derived Growth Factor Receptor Beta, PGR - Progesterone receptor, PI3K - Phosphoinositide 3-kinase, PKC - Protein kinase C, PPAT - Amidophosphoribosyltransferase, PTCL - Peripheral T cell Lymphoma, PTEN - Phosphatase and tensin homologue, RACK1 - Receptor for activated C kinase 1, RAN - RAs-related Nuclear protein, RAR - Retinoic acid receptor, RB1 - Retinoblastoma 1, RET - RET proto-oncogene, RFC - Reduced Folate Carrier, RON - Recepteur d'Origine Nantais, ROS - Reactive oxygen species, SERPINA7 - Serpin Family A Member 7, SHGB - Sex hormone-binding globulin, SLC22A2 - Solute Carrier Family 22 Member 2, SLC22A8 - Solute Carrier Family 22 Member 8, SLC47A1 - Solute Carrier Family 47 Member 1, SLC47A2 - Solute Carrier Family 47 Member 2, SLCO1B1 - Solute Carrier Organic Anion Transporter Family Member 1B1, SLCO1B3 - Solute Carrier Organic Anion Transporter Family Member 1B3, SLL - Small Lymphocytic Lymphoma, SMO - Smoothened, SRC - Proto-oncogene tyrosine-protein kinase Src, STAT - Signal transducer and activator of transcription, SUFU - Suppressor of fused homolog, SULT1A1 - Sulfotransferase Family 1A Member 1, SULT2A1 - Sulfotransferase Family 2A Member 1, TRK - Tropomyosin receptor kinase, TUBA4A - Tubulin Alpha 4a, TUBB1 - Tubulin Beta 1 Class VI, TUBB3 - Tubulin Beta 3 Class III, TWIST1 - Twist Family BHLH Transcription Factor 1, TYK1 - Leukocyte Tyrosine Kinase Receptor, TYMS - Thymidylate synthetase, UGT1A1 - UDP glucuronosyltransferase 1 family, polypeptide A1, UGT1A10 - UDP Glucuronosyltransferase Family 1 Member A10, UGT1A9 - UDP glucuronosyltransferase 1 family, polypeptide A9, XBP1 - X-box binding protein 1, XPO1 - Exportin 1, YES1 - Tyrosine-protein kinase Yes, ZAK - Mitogen-activated protein kinase kinase kinase MLT.

SUPPLEMENTARY TABLE 3. APPROVED DRUG COMBINATION OPTIONS ARE LIMITED, EVEN WHEN RECOMMENDED IN FIRST-LINE THERAPY FOR THE MOST PREVALENT AND FATAL CANCER TYPES

CANCER TYPE	Top 10 2016 Cancers by Death Counts*	Prevalence (2016) **	5-year Relative Survival (%) by Cancer Type, All Races, Both Sexes *	Number of FDA Approved Drugs ***	Number of FDA Approved Combinations ***	NCCN Guidelines to use drug combinations in first-line	FDA Approved combinations ***
**SMALL CELL LUNG CANCER**	148,869	303,842	19.1	9	0	Yes, but CDDP/VP-16+RT in stage 0-2 is the only "likely to cure" option.	None
**NON-SMALL CELL LUNG CANCER**				29	2	Yes, if unresectable or metastatic	CARBOPLATIN-TAXOL, GEMCITABINE-CISPLATIN
**BREAST**	41,487	1,000,721	89	36	6	Yes, neoadjuvant	AC, AC-T, CAF, CMF, FEC, TAC
**PROSTATE**	30,370	897,634	97.6	14	0	No	None
**COLONRECTAL**	52,286	466,817	63.8	15	8	Yes, if neoadjuvant or unresectable disease	CAPOX, FOLFIRI, FOLFIRI-BEVACIZUMAB, FOLFIRI-CETUXIMAB, FOLFOX, FU-LV, XELIRI, XELOX
**PANCREAS**	42,757	50,242	9.2	8	1	Yes, useful for control but unlikely to cure	Gemcitabine-Cisplatin
**OVARIAN CANCER**	14,223	65,881	46.6	14	8	Yes	BEP, CARBOPLATIN-TAXOL, GEMCITABINE-CISPLATIN, GEMCITABINE-CISPLATIN, JEB, PEB, VAC, VeIP
**LEUKEMIAS (ALL)**	23,287	144,092	55.6	20	1	Yes	Hyper-CVAD
**LEUKEMIAS (AML)**				17	1	Yes	ADE
**LEUKEMIAS (CLL)**				15	2	Yes	CHLORAMBUCIL-PREDNISONE, CVP
**LEUKEMIAS (CML)**				11	0	No, but possible if diagnosed at advanced phase	None
**LEUKEMIAS (HCL)**				3	0	Yes, but still unlikely to cure	None
**LIVER**	26,569	48,185	17.7	7	0	No	None
**NON-HODGKIN LYMPHOMA**	20,268	235,041	68.5	38	11	Yes, in some cases.	BR, CHOP, COPP, CVP, EPOCH, Hyper-CVAD, ICE, R-CHOP, R-CVP, R-EPOCH, R-ICE
**CORPUS AND UTERUS, NOS**	10,733	209,057	80.7	0	0	Yes, if unresectable or disseminated or metastatic, but unlikely to cure or long-term control.	None
**MELANOMA**	-----	303,842	89.6	12	0	No	None
**BLADDER**	-----	231,839	75.3	10	2	Yes, for stage II or higher.	GEMCITABINE-CISPLATIN

SUPPLEMENTARY TABLE 3. APPROVED DRUG COMBINATION OPTIONS ARE LIMITED, EVEN WHEN RECOMMENDED IN FIRST-LINE THERAPY FOR THE MOST PREVALENT AND FATAL CANCER TYPES (CONTINUATION)

CANCER TYPE	Top 10 2016 Cancers by Death Counts*	Prevalence (2016) **	5-year Relative Survival (%) by Cancer Type, All Races, Both Sexes *	Number of FDA Approved Drugs ***	Number of FDA Approved Combinations ***	Guideline to use combination CT in first-line (NCCN) ****	FDA Approved combinations ***
**THYROID**	-----	223,424	96.9	7	0	No	None
**KIDNEY**	-----	216,951	71.8	14	0	No	None
**MULTIPLE MYELOMA**	-----	73,207	46.7	17	1	Yes	PAD
**STOMACH**	-----	50,825	30.1	8	3	Yes, if unresectable or metastatic, but unlikely to cure.	FU-LV, TPF, XELIRI
**BRAIN**	-----	48,849	32.8	6	1	Yes	PCV
**CERVICAL**	-----	46,846	67.6	4	1	Yes	GEMCITABINE-CISPLATIN
**HODGKIN LYMPHOMA**	-----	37,108	83.6	15	13	Yes	ABVD, ABVE, ABVE-PC, BEACOPP, COPDAC, COPP, COPP-ABV, ICE, MOPP, OEPA, OPPA, STANFORD V, VAMP
**ESOPHAGUS**	-----	27,811	18.9	5	2	Yes	FU-LV, XELIRI

Abbreviations: ALL – acute lymphocytic leukemia, AML - acute myeloid leukemia, CLL – chronic lymphocytic leukemia, CML – chronic myeloid leukemia, HCL – hairy cell leukemia, RT – radiotherapy, CT – chemotherapy, CDDP – cisplatin, VP16 – etoposide, AC – doxorubicin and cyclophosphamide, AC-T – doxorubicin, cyclophosphamide and paclitaxel.

* **U.S. CANCER STATISTICS WORKING GROUP. U.S. CANCER STATISTICS DATA VISUALIZATIONS TOOL, BASED ON NOVEMBER 2018 SUBMISSION DATA (1999-2016): U.S. DEPARTMENT OF HEALTH AND HUMAN SERVICES, CENTERS FOR DISEASE CONTROL AND PREVENTION AND NATIONAL CANCER INSTITUTE; WWW.CDC.GOV/CANCER/DATAVIZ, JUNE 2019**.

** Estimated prevalence counts, by cancer type, race, and sex, 5-year limited duration, united states, invasive cancers only, on January 1, 2016.

******* Data available at https://www.cancer.gov/about-cancer/treatment/drugs

**** Data available at https://www.nccn.org/professionals/physician_gls/default.aspx

SUPPLEMENTARY REFERENCES

1. Fink, D.; Aebi, S.; Howell, S.B. The role of DNA mismatch repair in drug resistance. *Clin. Cancer Res.* 1998.

2. Valdez, B.C.; Murray, D.; Ramdas, L.; de Lima, M.; Jones, R.; Kornblau, S.; Betancourt, D.; Li, Y.; Champlin, R.E.; Andersson, B.S. Altered gene expression in busulfan-resistant human myeloid leukemia. *Leuk. Res.* **2008**.

3. Sun, J.; Wei, Q.; Zhou, Y.; Wang, J.; Liu, Q.; Xu, H. A systematic analysis of FDA-approved anticancer drugs. *BMC Syst. Biol.* **2017**, *11*, 87.

4. Choi, C.H. ABC transporters as multidrug resistance mechanisms and the development of chemosensitizers for their reversal. *Cancer Cell Int.* 2005.

5. Srivastava, S.K.; Singhal, S.S.; Hu, X.; Awasthi, Y.C.; Zimniak, P.; Singh, S. V. Differential catalytic efficiency of allelic variants of human glutathione S-transferase Pi in catalyzing the glutathione conjugation of thiotepa. *Arch. Biochem. Biophys.* **1999**.

6. Li, G.M. Mechanisms and functions of DNA mismatch repair. *Cell Res.* 2008.

7. Tweedie, D.J.; Fernandez, D.; Spearman, M.E.; Feldhoff, R.C.; Prough, R.A. Metabolism of azoxy derivatives of procarbazine by aldehyde dehydrogenase and xanthine oxidase. *Drug Metab. Dispos. 19*, 793–803.

8. Montraveta, A.; Lee-Vergés, E.; Roldán, J.; Jiménez, L.; Cabezas, S.; Clot, G.; Pinyol, M.; Xargay-Torrent, S.; Rosich, L.; Arimany-Nardí, C.; et al. CD69 expression potentially predicts response to bendamustine and its modulation by ibrutinib or idelalisib enhances cytotoxic effect in chronic lymphocytic leukemia. *Oncotarget* **2016**.

9. Sau, A.; Pellizzari Tregno, F.; Valentino, F.; Federici, G.; Caccuri, A.M. Glutathione transferases and development of new principles to overcome drug resistance. *Arch. Biochem. Biophys.* 2010.

10. Backos, D.S.; Franklin, C.C.; Reigan, P. The role of glutathione in brain tumor drug resistance. *Biochem. Pharmacol.* 2012.

11. Ekhart, C.; Doodeman, V.D.; Rodenhuis, S.; Smits, P.H.M.; Beijnen, J.H.; Huitema, A.D.R. Influence of polymorphisms of drug metabolizing enzymes (CYP2B6, CYP2C9, CYP2C19, CYP3A4, CYP3A5, GSTA1, GSTP1, ALDH1A1 and ALDH3A1) on the pharmacokinetics of cyclophosphamide and 4-hydroxycyclophosphamide. *Pharmacogenet. Genomics* **2008**.

12. Wang, X.; Zhang, J.; Xu, T. Thioredoxin reductase inactivation as a pivotal mechanism of ifosfamide in cancer therapy. *Eur. J. Pharmacol.* **2008**.

13. Melguizo, C.; Prados, J.; Rama, A.R.; Ortiz, R.; Álvarez, P.J.; Fernández, J.E.; Aranega, A. Multidrug resistance and rhabdomyosarcoma (review). *Oncol. Rep.* **2011**.

14. Cheng, J.; Ye, F.; Dan, G.; Zhao, Y.; Zhao, J.; Zou, Z. Formation and degradation of nitrogen mustard-induced MGMT-DNA crosslinking in 16HBE cells. *Toxicology* **2017**.

15. Zhang, J.; Ye, Z.; Lou, Y. Metabolism of melphalan by rat liver microsomal glutathione S-transferase. *Chem. Biol. Interact.* **2005**.

16. Chauhan, D.; Velankar, M.; Brahmandam, M.; Hideshima, T.; Podar, K.; Richardson, P.; Schlossman, R.; Ghobrial, I.; Raje, N.; Munshi, N.; et al. A novel Bcl-2/Bcl-XL/Bcl-w inhibitor

ABT-737 as therapy in multiple myeloma. *Oncogene* **2007**.

17. Esteller, M.; Garcia-Foncillas, J.; Andion, E.; Goodman, S.N.; Hidalgo, O.F.; Vanaclocha, V.; Baylin, S.B.; Herman, J.G. Inactivation of the DNA-Repair Gene MGMT and the Clinical Response of Gliomas to Alkylating Agents . *N. Engl. J. Med.* **2002**.

18. Diedrich, A.; Bock, H.C.; König, F.; Schulz, T.G.; Ludwig, H.C.; Herken, R.; Quondamatteo, F. Expression of glutathione S-transferase T1 (GSTT1) in human brain tumours. *Histol. Histopathol.* **2006**.

19. Hung, C.F. Effects of carmustine and lomustine on arylamine N-acetyltransferase activity and 2-aminofluorene-DNA adducts in rat glial tumor cells. *Neurochem. Res.* **2000**.

20. Ribrag, V.; Massaad, L.; Janot, F.; Bissery, M.C.; Parise, O.; Gouyette, A.; Chabot, G.G. Principal drug-metabolizing enzyme systems in L1210 leukemia sensitive or resistant to BCNU in vivo. *Leuk. Res.* **1994**.

21. Larkin, J.M.G.; Hughes, S.A.; Beirne, D.A.; Patel, P.M.; Gibbens, I.M.; Bate, S.C.; Thomas, K.; Eisen, T.G.; Gore, M.E. A Phase I/II study of lomustine and temozolomide in patients with cerebral metastases from malignant melanoma. *Br. J. Cancer* **2007**.

22. Passagne, I.; Evrard, A.; Depeille, P.; Cuq, P.; Cupissol, D.; Vian, L. O6-methylguanine DNA-methyltransferase (MGMT) overexpression in melanoma cells induces resistance to nitrosoureas and temozolomide but sensitizes to mitomycin C. *Toxicol. Appl. Pharmacol.* **2006**.

23. Tentori, L.; Lacal, P.M.; Graziani, G. Challenging resistance mechanisms to therapies for metastatic melanoma. *Trends Pharmacol. Sci.* 2013.

24. Okada, M.; Sato, A.; Shibuya, K.; Watanabe, E.; Seino, S.; Suzuki, S.; Seino, M.; Narita, Y.; Shibui, S.; Kayama, T.; et al. JNK contributes to temozolomide resistance of stem-like glioblastoma cells via regulation of MGMT expression. *Int. J. Oncol.* **2014**.

25. Stewart, D.J. Mechanisms of resistance to cisplatin and carboplatin. *Crit. Rev. Oncol. Hematol.* 2007.

26. Shi, T.Y.; Yang, G.; Tu, X.Y.; Yang, J.M.; Qian, J.; Wu, X.H.; Zhou, X.Y.; Cheng, X.; Wei, Q. RAD52 Variants Predict Platinum Resistance and Prognosis of Cervical Cancer. *PLoS One* **2012**.

27. Galluzzi, L.; Senovilla, L.; Vitale, I.; Michels, J.; Martins, I.; Kepp, O.; Castedo, M.; Kroemer, G. Molecular mechanisms of cisplatin resistance. *Oncogene* 2012.

28. Gourdier, I.; Del Rio, M.; Crabbé, L.; Candeil, L.; Copois, V.; Ychou, M.; Auffray, C.; Martineau, P.; Mechti, N.; Pommier, Y.; et al. Drug specific resistance to oxaliplatin is associated with apoptosis defect in a cellular model of colon carcinoma. *FEBS Lett.* **2002**.

29. Du, H.; Yang, W.; Chen, L.; Shi, M.; Seewoo, V.; Wang, J.; Lin, A.; Liu, Z.; Qiu, W. Role of autophagy in resistance to oxaliplatin in hepatocellular carcinoma cells. *Oncol. Rep.* **2012**.

30. Moutinho, C.; Martinez-Cardús, A.; Santos, C.; Navarro-Pérez, V.; Martínez-Balibrea, E.; Musulen, E.; Carmona, F.J.; Sartore-Bianchi, A.; Cassingena, A.; Siena, S.; et al. Epigenetic inactivation of the BRCA1 interactor SRBC and resistance to oxaliplatin in colorectal cancer. *J. Natl. Cancer Inst.* **2014**.

31. Hattinger, C.M.; Reverter-Branchat, G.; Remondini, D.; Castellani, G.C.; Benini, S.; Pasello, M.; Manara, M.C.; Scotlandi, K.; Picci, P.; Serra, M. Genomic imbalances associated with methotrexaate resistance in human osteosarcoma cell lines detected by comparative genomic

hybridization-based techniques. *Eur. J. Cell Biol.* **2003**.

32. Stark, M.; Wichman, C.; Avivi, I.; Assaraf, Y.G. Aberrant splicing of folylpolyglutamate synthetase as a novel mechanism of antifolate resistance in leukemia. *Blood* **2009**.

33. Assaraf, Y.G. Molecular basis of antifolate resistance. *Cancer Metastasis Rev.* 2007.

34. Serova, M.; Bieche, I.; Sablin, M.P.; Pronk, G.J.; Vidaud, M.; Cvitkovic, E.; Faivre, S.; Raymond, E. Single agent and combination studies of pralatrexate and molecular correlates of sensitivity. *Br. J. Cancer* **2011**.

35. Cluzeau, T.; Robert, G.; Mounier, N.; Karsenti, J.M.; Dufies, M.; Puissant, A.; Jacquel, A.; Renneville, A.; Preudhomme, C.; Cassuto, J.P.; et al. BCL2L10 is a predictive factor for resistance to Azacitidine in MDS and AML patients. *Oncotarget* **2012**.

36. Wang, W.; McLeod, H.L.; Cassidy, J.; Collie-Duguid, E.S.R. Mechanisms of acquired chemoresistance to 5-fluorouracil and tomudex: Thymidylate synthase dependent and independent networks. *Cancer Chemother. Pharmacol.* **2007**.

37. Jordheim, L.P.; Dumontet, C. Review of recent studies on resistance to cytotoxic deoxynucleoside analogues. *Biochim. Biophys. Acta - Rev. Cancer* 2007.

38. Zhenchuk, A.; Lotfi, K.; Juliusson, G.; Albertioni, F. Mechanisms of anti-cancer action and pharmacology of clofarabine. *Biochem. Pharmacol.* 2009.

39. Zahreddine, H.; Borden, K.L.B. Mechanisms and insights into drug resistance in cancer. *Front. Pharmacol.* 2013.

40. Bejar, R.; Lord, A.; Stevenson, K.; Bar-Natan, M.; Pérez-Ladaga, A.; Zaneveld, J.; Wang, H.; Caughey, B.; Stojanov, P.; Getz, G.; et al. TET2 mutations predict response to hypomethylating agents in myelodysplastic syndrome patients. *Blood* **2014**.

41. Qin, T.; Castoro, R.; El Ahdab, S.; Jelinek, J.; Wang, X.; Si, J.; Shu, J.; He, R.; Zhang, N.; Chung, W.; et al. Mechanisms of resistance to decitabine in the myelodysplastic syndrome. *PLoS One* **2011**.

42. Murakami, Y.; Kazuno, H.; Emura, T.; Tsujimoto, H.; Suzuki, N.; Fukushima, M. Different mechanisms of acquired resistance to fluorinated pyrimidines in human colorectal cancer cells. *Int. J. Oncol.* **2000**.

43. Tsume, Y.; Hilfinger, J.M.; Amidon, G.L. Enhanced Cancer Cell Growth Inhibition by Dipeptide Prodrugs of Floxuridine: Increased Transporter Affinity and Metabolic Stability. *Mol. Pharm.* **2008**, *5*, 717–727.

44. Gunes, A.; Coskun, U.; Boruban, C.; Gunel, N.; Babaoglu, M.O.; Sencan, O.; Bozkurt, A.; Rane, A.; Hassan, M.; Zengil, H.; et al. Inhibitory effect of 5-fluorouracil on cytochrome P450 2C9 activity in cancer patients. *Basic Clin. Pharmacol. Toxicol.* **2006**, *98*, 197–200.

45. Zenz, T.; Häbe, S.; Denzel, T.; Mohr, J.; Winkler, D.; Bühler, A.; Sarno, A.; Groner, S.; Mertens, D.; Busch, R.; et al. Detailed analysis of p53 pathway defects in fludarabine-refractory chronic lymphocytic leukemia (CLL): Dissecting the contribution of 17p deletion, TP53 mutation, p53-p21 dysfunction, and miR34a in a prospective clinical trial. *Blood* **2009**.

46. Moussay, E.; Palissot, V.; Vallar, L.; Poirel, H.A.; Wenner, T.; El Khoury, V.; Aouali, N.; Van Moer, K.; Leners, B.; Bernardin, F.; et al. Determination of genes and microRNAs involved in the resistance to fludarabine in vivo in chronic lymphocytic leukemia. *Mol. Cancer* **2010**.

47. Mini, E.; Nobili, S.; Caciagli, B.; Landini, I.; Mazzei, T. Cellular pharmacology of gemcitabine. *Ann. Oncol.* **2006**.

48. Wu, C.; Li, W. Genomics and pharmacogenomics of pediatric acute lymphoblastic leukemia. *Crit. Rev. Oncol. Hematol.* 2018.

49. Lotfi, K.; MÅnsson, E.; Peterson, C.; Eriksson, S.; Albertioni, F. Low level of mitochondrial deoxyguanosine kinase is the dominant factor in acquired resistance to 9-β-D-arabinofuranosylguanine cytotoxicity. *Biochem. Biophys. Res. Commun.* **2002**.

50. Pan, Y.; Zhang, Y.; Liu, W.; Huang, Y.; Shen, X.; Jing, R.; Pu, J.; Wang, X.; Ju, S.; Cong, H.; et al. LncRNA H19 overexpression induces bortezomib resistance in multiple myeloma by targeting MCL-1 via miR-29b-3p. *Cell Death Dis.* **2019**.

51. Kovacic, P. Hydroxyurea (therapeutics and mechanism): Metabolism, carbamoyl nitroso, nitroxyl, radicals, cell signaling and clinical applications. *Med. Hypotheses* **2011**.

52. Forli, S. Epothilones: From Discovery to Clinical Trials. *Curr. Top. Med. Chem.* **2014**.

53. Oba, T.; Izumi, H.; Ito, K. ABCB1 and ABCC11 confer resistance to eribulin in breast cancer cell lines. *Oncotarget* **2016**.

54. Zhang, Z.Y.; King, B.M.; Pelletier, R.D.; Wong, Y.N. Delineation of the interactions between the chemotherapeutic agent eribulin mesylate (E7389) and human CYP3A4. *Cancer Chemother. Pharmacol.* **2008**.

55. Gillet, J.-P.; Gottesman, M.M. Mechanisms of multidrug resistance in cancer. *Methods Mol. Biol.* **2010**.

56. O’Neill, A.J.; Prencipe, M.; Dowling, C.; Fan, Y.; Mulrane, L.; Gallagher, W.M.; O’Connor, D.; O’Connor, R.; Devery, A.; Corcoran, C.; et al. Characterisation and manipulation of docetaxel resistant prostate cancer cell lines. *Mol. Cancer* **2011**.

57. Ben-Hamo, R.; Zilberberg, A.; Cohen, H.; Bahar-Shany, K.; Wachtel, C.; Korach, J.; Aviel-Ronen, S.; Barshack, I.; Barash, D.; Levanon, K.; et al. Resistance to paclitaxel is associated with a variant of the gene BCL2 in multiple tumor types. *npj Precis. Oncol.* **2019**, *3*, 12.

58. Zhang, Y.; Yang, S.H.; Guo, X.L. New insights into Vinca alkaloids resistance mechanism and circumvention in lung cancer. *Biomed. Pharmacother.* 2017.

59. Kavallaris, M. Microtubules and resistance to tubulin-binding agents. *Nat. Rev. Cancer* 2010.

60. Yoshida, M.; Matsui, Y.; Ikarashi, Y.; Usui, T.; Osada, H.; Wakasugi, H. Antiproliferating activity of the mitotic inhibitor pironetin against vindesine- and paclitaxel-resistant human small cell lung cancer H69 cells. *Anticancer Res.* **2007**.

61. Zhong, L.; Shen, H.; Huang, C.; Jing, H.; Cao, D. AKR1B10 induces cell resistance to daunorubicin and idarubicin by reducing C13 ketonic group. *Toxicol. Appl. Pharmacol.* **2011**.

62. Oakman, C.; Moretti, E.; Galardi, F.; Santarpia, L.; Di Leo, A. The role of topoisomerase IIα and HER-2 in predicting sensitivity to anthracyclines in breast cancer patients. *Cancer Treat. Rev.* 2009.

63. Perabo, F.G.E.; Müller, S.C. New agents in intravesical chemotherapy of superficial bladder cancer. *Scand. J. Urol. Nephrol.* **2005**, *39*, 108–16.

64. Morrow, C.S. Multidrug Resistance Protein 1 (MRP1, ABCC1) Mediates Resistance to Mitoxantrone via Glutathione-Dependent Drug Efflux. *Mol. Pharmacol.* **2006**.

65. Lu, J.; Chew, E.-H.; Holmgren, A. Targeting thioredoxin reductase is a basis for cancer therapy by arsenic trioxide. *Proc. Natl. Acad. Sci.* **2007**, *104*, 12288–12293.

66. Ouyang, W.; Li, J.; Ma, Q.; Huang, C. Essential roles of PI-3K/Akt/IKKβ/NFκB pathway in cyclin D1 induction by arsenite in JB6 Cl41 cells. *Carcinogenesis* **2006**, *27*, 864–873.

67. Huang, H.-S.; Liu, Z.-M.; Hong, D.-Y. Blockage of JNK pathway enhances arsenic trioxide-induced apoptosis in human keratinocytes. *Toxicol. Appl. Pharmacol.* **2010**, *244*, 234–241.

68. Zhang, X.W.; Yan, X.J.; Zhou, Z.R.; Yang, F.F.; Wu, Z.Y.; Sun, H.B.; Liang, W.X.; Song, A.X.; Lallemand-Breitenbach, V.; Jeanne, M.; et al. Arsenic Trioxide Controls the Fate of the PML-RAR Oncoprotein by Directly Binding PML. *Science (80-.).* **2010**, *328*, 240–243.

69. Zhang, X.; Pan, J. Resistance to arsenic trioxide and retinoic acid therapy in acute promyelocytic leukemia. *Ann. Hematol.* **2017**, *96*, 707–708.

70. Xu, Y.; Villalona-Calero, M.A. Irinotecan: Mechanisms of tumor resistance and novel strategies for modulating its activity. *Ann. Oncol.* 2002.

71. Meisenberg, C.; Ashour, M.E.; El-Shafie, L.; Liao, C.; Hodgson, A.; Pilborough, A.; Khurram, S.A.; Downs, J.A.; Ward, S.E.; El-Khamisy, S.F. Epigenetic changes in histone acetylation underpin resistance to the topoisomerase I inhibitor irinotecan. *Nucleic Acids Res.* **2017**.

72. Ricci, J.W.; Lovato, D.M.; Severns, V.; Sklar, L.A.; Larson, R.S. Novel ABCG2 Antagonists Reverse Topotecan-Mediated Chemotherapeutic Resistance in Ovarian Carcinoma Xenografts. *Mol. Cancer Ther.* **2016**.

73. Daniel, R.A.; Rozanska, A.L.; Thomas, H.D.; Mulligan, E.A.; Drew, Y.; Castelbuono, D.J.; Hostomsky, Z.; Plummer, E.R.; Boddy, A. V.; Tweddie, D.A.; et al. Inhibition of poly(ADP-Ribose) polymerase-1 enhances temozolomide and topotecan activity against childhood Neuroblastoma. *Clin. Cancer Res.* **2009**.

74. Klejewski, A.; Świerczewska, M.; Zaorska, K.; Brązert, M.; Nowicki, M.; Zabel, M.; Januchowski, R. New and Old Genes Associated with Topotecan Resistance Development in Ovarian Cancer Cell Lines. *Anticancer Res.* **2017**, *37*, 1625–1636.

75. Pauli-Magnus, C.; Kroetz, D.L. Functional implications of genetic polymorphisms in the multidrug resistance gene MDR1 (ABCB1). *Pharm. Res.* 2004.

76. Lindhe, Ö.; Skogseid, B.; Brandt, I. Cytochrome P450-Catalyzed Binding of 3-Methylsulfonyl-DDE and o,p′-DDD in Human Adrenal Zona Fasciculata/Reticularis. *J. Clin. Endocrinol. Metab.* **2002**, *87*, 1319–1326.

77. Cai, W.; Counsell, R.E.; Schteingart, D.E.; Sinsheimer, J.E.; Vaz, A.D.N.; Wotring, L.L. Adrenal proteins bound by a reactive intermediate of mitotane. *Cancer Chemother. Pharmacol.* **1997**, *39*, 537–540.

78. Do, P.M.; Varanasi, L.; Fan, S.; Li, C.; Kubacka, I.; Newman, V.; Chauhan, K.; Daniels, S.R.; Boccetta, M.; Garrett, M.R.; et al. Mutant p53 cooperates with ETS2 to promote etoposide resistance. *Genes Dev.* **2012**.

79. Pan, S.T.; Li, Z.L.; He, Z.X.; Qiu, J.X.; Zhou, S.F. Molecular mechanisms for tumour resistance to chemotherapy. *Clin. Exp. Pharmacol. Physiol.* 2016.

80. Dortet, L.; Girlich, D.; Virlouvet, A.-L.; Poirel, L.; Nordmann, P.; Iorga, B.I.; Naas, T. Characterization of BRP MBL , the Bleomycin Resistance Protein Associated with the Carbapenemase NDM. *Antimicrob. Agents Chemother.* **2017**, *61*.

81. Goodman, L. S., Gilman, A., Brunton, L. L., Lazo, J. S., & Parker, K.L. *Goodman & Gilman's the pharmacological basis of therapeutics*; 2006; ISBN 9788578110796.

82. Mader, M.M.; Henry, J.R. Antimetabolites. In *Comprehensive Medicinal Chemistry II*; Taylor, J.B., Triggle, D.J., Eds.; Elsevier: London, UK, 2007; pp. 55–79 ISBN 978-0-08-045044-5.

83. Gonen, N.; Assaraf, Y.G. Antifolates in cancer therapy: structure, activity and mechanisms of drug resistance. *Drug Resist. Updat.* **2012**, *15*, 183–210.

84. Pieters, R.; Hunger, S.P.; Boos, J.; Rizzari, C.; Silverman, L.; Baruchel, A.; Goekbuget, N.; Schrappe, M.; Pui, C.-H. L-asparaginase treatment in acute lymphoblastic leukemia. *Cancer* **2011**, *117*, 238–249.

85. Greenberger, L.M.; Sampath, D. Resistance To Taxanes. In *Cancer Drug Resistance*; Teicher, B.A., Ed.; Humana Press: Totowa, NJ, 2006; pp. 329–358.

86. Duran, G.E.; Wang, Y.C.; Francisco, E.B.; Rose, J.C.; Martinez, F.J.; Coller, J.; Brassard, D.; Vrignaud, P.; Sikic, B.I. Mechanisms of Resistance to Cabazitaxel. *Mol. Cancer Ther.* **2015**, *14*, 193–201.

87. Crona, D.; Whang, Y. Androgen Receptor-Dependent and -Independent Mechanisms Involved in Prostate Cancer Therapy Resistance. *Cancers (Basel).* **2017**, *9*, 67.

88. Dhawan, M.; Ryan, C.J. Utility of novel androgen receptor therapies in the real world: A nuanced approach. *Urol. Oncol. Semin. Orig. Investig.* **2016**, *34*, 340–347.

89. Fan, M.; Yan, P.S.; Hartman-Frey, C.; Chen, L.; Paik, H.; Oyer, S.L.; Salisbury, J.D.; Cheng, A.S.L.; Li, L.; Abbosh, P.H.; et al. Diverse Gene Expression and DNA Methylation Profiles Correlate with Differential Adaptation of Breast Cancer Cells to the Antiestrogens Tamoxifen and Fulvestrant. *Cancer Res.* **2006**, *66*, 11954–11966.

90. Frogne, T.; Benjaminsen, R. V.; Sonne-Hansen, K.; Sorensen, B.S.; Nexo, E.; Laenkholm, A.-V.; Rasmussen, L.M.; Riese, D.J.; de Cremoux, P.; Stenvang, J.; et al. Activation of ErbB3, EGFR and Erk is essential for growth of human breast cancer cell lines with acquired resistance to fulvestrant. *Breast Cancer Res. Treat.* **2009**, *114*, 263–275.

91. Clarke, R.; Liu, M.C.; Bouker, K.B.; Gu, Z.; Lee, R.Y.; Zhu, Y.; Skaar, T.C.; Gomez, B.; O'Brien, K.; Wang, Y.; et al. Antiestrogen resistance in breast cancer and the role of estrogen receptor signaling. *Oncogene* **2003**, *22*, 7316–7339.

92. Arpino, G.; Weiss, H.; Lee, A. V.; Schiff, R.; De Placido, S.; Osborne, C.K.; Elledge, R.M. Estrogen Receptor–Positive, Progesterone Receptor–Negative Breast Cancer: Association With Growth Factor Receptor Expression and Tamoxifen Resistance. *JNCI J. Natl. Cancer Inst.* **2005**, *97*, 1254–1261.

93. Sarvilinna, N.; Eronen, H.; Miettinen, S.; Vienonen, A.; Ylikomi, T. Steroid hormone receptors and coregulators in endocrine-resistant and estrogen-independent breast cancer cells. *Int. J. Cancer* **2006**, *118*, 832–840.

94. Mostaghel, E.A.; Marck, B.T.; Plymate, S.R.; Vessella, R.L.; Balk, S.; Matsumoto, A.M.; Nelson, P.S.; Montgomery, R.B. Resistance to CYP17A1 Inhibition with Abiraterone in Castration-

Resistant Prostate Cancer: Induction of Steroidogenesis and Androgen Receptor Splice Variants. *Clin. Cancer Res.* **2011**, *17*, 5913–5925.

95. Buttigliero, C.; Tucci, M.; Bertaglia, V.; Vignani, F.; Bironzo, P.; Di Maio, M.; Scagliotti, G.V. Understanding and overcoming the mechanisms of primary and acquired resistance to abiraterone and enzalutamide in castration resistant prostate cancer. *Cancer Treat. Rev.* **2015**, *41*, 884–892.

96. Ma, C.X.; Reinert, T.; Chmielewska, I.; Ellis, M.J. Mechanisms of aromatase inhibitor resistance. *Nat. Rev. Cancer* **2015**, *15*, 261–275.

97. Vilquin, P.; Villedieu, M.; Grisard, E.; Larbi, S. Ben; Ghayad, S.E.; Heudel, P.-E.; Bachelot, T.; Corbo, L.; Treilleux, I.; Vendrell, J.A.; et al. Molecular characterization of anastrozole resistance in breast cancer: Pivotal role of the Akt/mTOR pathway in the emergence of de novo or acquired resistance and importance of combining the allosteric Akt inhibitor MK-2206 with an aromatase inhibitor. *Int. J. Cancer* **2013**, *133*, 1589–1602.

98. Katoh, M. Genomic testing, tumor microenvironment and targeted therapy of Hedgehog-related human cancers. *Clin. Sci.* **2019**, *133*, 953–970.

99. Dong, X.; Wang, C.; Chen, Z.; Zhao, W. Overcoming the resistance mechanisms of Smoothened inhibitors. *Drug Discov. Today* **2018**, *23*, 704–710.

100. Lee, J.-H.; Choy, M.L.; Marks, P.A. Mechanisms of Resistance to Histone Deacetylase Inhibitors. In *Advances in Cancer Research*; 2012; pp. 39–86.

101. Epping, M.T.; Wang, L.; Plumb, J.A.; Lieb, M.; Gronemeyer, H.; Brown, R.; Bernards, R. A functional genetic screen identifies retinoic acid signaling as a target of histone deacetylase inhibitors. *Proc. Natl. Acad. Sci.* **2007**, *104*, 17777–17782.

102. Shao, W.; Growney, J.D.; Feng, Y.; O'Connor, G.; Pu, M.; Zhu, W.; Yao, Y.-M.; Kwon, P.; Fawell, S.; Atadja, P. Activity of deacetylase inhibitor panobinostat (LBH589) in cutaneous T-cell lymphoma models: Defining molecular mechanisms of resistance. *Int. J. Cancer* **2010**, *127*, 2199–2208.

103. Katayama, R.; Friboulet, L.; Koike, S.; Lockerman, E.L.; Khan, T.M.; Gainor, J.F.; Iafrate, A.J.; Takeuchi, K.; Taiji, M.; Okuno, Y.; et al. Two Novel ALK Mutations Mediate Acquired Resistance to the Next-Generation ALK Inhibitor Alectinib. *Clin. Cancer Res.* **2014**, *20*, 5686–5696.

104. Huang, W.-S.; Liu, S.; Zou, D.; Thomas, M.; Wang, Y.; Zhou, T.; Romero, J.; Kohlmann, A.; Li, F.; Qi, J.; et al. Discovery of Brigatinib (AP26113), a Phosphine Oxide-Containing, Potent, Orally Active Inhibitor of Anaplastic Lymphoma Kinase. *J. Med. Chem.* **2016**, *59*, 4948–4964.

105. Rothenstein, J.M.; Chooback, N. ALK inhibitors, resistance development, clinical trials. *Curr. Oncol.* **2018**, *25*, 59.

106. Gainor, J.F.; Dardaei, L.; Yoda, S.; Friboulet, L.; Leshchiner, I.; Katayama, R.; Dagogo-Jack, I.; Gadgeel, S.; Schultz, K.; Singh, M.; et al. Molecular Mechanisms of Resistance to First- and Second-Generation ALK Inhibitors in ALK-Rearranged Lung Cancer. *Cancer Discov.* **2016**, *6*, 1118–1133.

107. Zhang, S.; Anjum, R.; Squillace, R.; Nadworny, S.; Zhou, T.; Keats, J.; Ning, Y.; Wardwell, S.D.; Miller, D.; Song, Y.; et al. The Potent ALK Inhibitor Brigatinib (AP26113) Overcomes Mechanisms of Resistance to First- and Second-Generation ALK Inhibitors in Preclinical Models. *Clin. Cancer Res.* **2016**, *22*, 5527–5538.

108. Waqar, S.N.; Morgensztern, D. Lorlatinib: a new-generation drug for ALK-positive NSCLC. *Lancet Oncol.* **2018**, *19*, 1555–1557.

109. Patel, A.B.; O'Hare, T.; Deininger, M.W. Mechanisms of Resistance to ABL Kinase Inhibition in Chronic Myeloid Leukemia and the Development of Next Generation ABL Kinase Inhibitors. *Hematol. Oncol. Clin. North Am.* **2017**, *31*, 589–612.

110. Patnaik, A.; Rosen, L.S.; Tolaney, S.M.; Tolcher, A.W.; Goldman, J.W.; Gandhi, L.; Papadopoulos, K.P.; Beeram, M.; Rasco, D.W.; Hilton, J.F.; et al. Efficacy and Safety of Abemaciclib, an Inhibitor of CDK4 and CDK6, for Patients with Breast Cancer, Non-Small Cell Lung Cancer, and Other Solid Tumors. *Cancer Discov.* **2016**, *6*, 740–753.

111. Rocca, A.; Schirone, A.; Maltoni, R.; Bravaccini, S.; Cecconetto, L.; Farolfi, A.; Bronte, G.; Andreis, D. Progress with palbociclib in breast cancer: latest evidence and clinical considerations. *Ther. Adv. Med. Oncol.* **2017**, *9*, 83–105.

112. Rader, J.; Russell, M.R.; Hart, L.S.; Nakazawa, M.S.; Belcastro, L.T.; Martinez, D.; Li, Y.; Carpenter, E.L.; Attiyeh, E.F.; Diskin, S.J.; et al. Dual CDK4/CDK6 Inhibition Induces Cell-Cycle Arrest and Senescence in Neuroblastoma. *Clin. Cancer Res.* **2013**, *19*, 6173–6182.

113. Chae, Y.K.; Ranganath, K.; Hammerman, P.S.; Vaklavas, C.; Mohindra, N.; Kalyan, A.; Matsangou, M.; Costa, R.; Carneiro, B.; Villaflor, V.M.; et al. Inhibition of the fibroblast growth factor receptor (FGFR) pathway: the current landscape and barriers to clinical application. *Oncotarget* **2017**, *8*.

114. Zhou, C.; Chen, T.; Xie, Z.; Qin, Y.; Ou, Y.; Zhang, J.; Li, S.; Chen, R.; Zhong, N. RACK1 forms a complex with FGFR1 and PKM2, and stimulates the growth and migration of squamous lung cancer cells. *Mol. Carcinog.* **2017**, *56*, 2391–2399.

115. Mayer, I.A.; Abramson, V.G.; Formisano, L.; Balko, J.M.; Estrada, M. V.; Sanders, M.E.; Juric, D.; Solit, D.; Berger, M.F.; Won, H.H.; et al. A Phase Ib Study of Alpelisib (BYL719), a PI3Kα-Specific Inhibitor, with Letrozole in ER + /HER2 − Metastatic Breast Cancer. *Clin. Cancer Res.* **2017**, *23*, 26–34.

116. Patnaik, A.; Appleman, L.J.; Tolcher, A.W.; Papadopoulos, K.P.; Beeram, M.; Rasco, D.W.; Weiss, G.J.; Sachdev, J.C.; Chadha, M.; Fulk, M.; et al. First-in-human phase I study of copanlisib (BAY 80-6946), an intravenous pan-class I phosphatidylinositol 3-kinase inhibitor, in patients with advanced solid tumors and non-Hodgkin's lymphomas. *Ann. Oncol.* **2016**, *27*, 1928–1940.

117. Faia, K.; White, K.; Murphy, E.; Proctor, J.; Pink, M.; Kosmider, N.; McGovern, K.; Kutok, J. The phosphoinositide-3 kinase (PI3K)-δ,γ inhibitor, duvelisib shows preclinical synergy with multiple targeted therapies in hematologic malignancies. *PLoS One* **2018**, *13*.

118. Horwitz, S.M.; Koch, R.; Porcu, P.; Oki, Y.; Moskowitz, A.; Perez, M.; Myskowski, P.; Officer, A.; Jaffe, J.D.; Morrow, S.N.; et al. Activity of the PI3K-δ,γ inhibitor duvelisib in a phase 1 trial and preclinical models of T-cell lymphoma. *Blood* **2018**, *131*, 888–898.

119. Cheah, C.Y.; Fowler, N.H. Idelalisib in the management of lymphoma. *Blood* **2016**, *128*, 331–336.

120. Rizos, H.; Menzies, A.M.; Pupo, G.M.; Carlino, M.S.; Fung, C.; Hyman, J.; Haydu, L.E.; Mijatov, B.; Becker, T.M.; Boyd, S.C.; et al. BRAF Inhibitor Resistance Mechanisms in Metastatic Melanoma: Spectrum and Clinical Impact. *Clin. Cancer Res.* **2014**, *20*, 1965–1977.

121. Koelblinger, P.; Thuerigen, O.; Dummer, R. Development of encorafenib for BRAF-mutated advanced melanoma. *Curr. Opin. Oncol.* **2018**, 1.

122. Oddo, D.; Sennott, E.M.; Barault, L.; Valtorta, E.; Arena, S.; Cassingena, A.; Filiciotto, G.; Marzolla, G.; Elez, E.; van Geel, R.M.J.M.; et al. Molecular Landscape of Acquired Resistance to Targeted Therapy Combinations in BRAF-Mutant Colorectal Cancer. *Cancer Res.* **2016**, *76*, 4504–4515.

123. Trunzer, K.; Pavlick, A.C.; Schuchter, L.; Gonzalez, R.; McArthur, G.A.; Hutson, T.E.; Moschos, S.J.; Flaherty, K.T.; Kim, K.B.; Weber, J.S.; et al. Pharmacodynamic Effects and Mechanisms of Resistance to Vemurafenib in Patients With Metastatic Melanoma. *J. Clin. Oncol.* **2013**, *31*, 1767–1774.

124. Li, J.-L.; Sainson, R.C.A.; Oon, C.E.; Turley, H.; Leek, R.; Sheldon, H.; Bridges, E.; Shi, W.; Snell, C.; Bowden, E.T.; et al. DLL4-Notch Signaling Mediates Tumor Resistance to Anti-VEGF Therapy In Vivo. *Cancer Res.* **2011**, *71*, 6073–6083.

125. Piao, Y.; Liang, J.; Holmes, L.; Henry, V.; Sulman, E.; de Groot, J.F. Acquired Resistance to Anti-VEGF Therapy in Glioblastoma Is Associated with a Mesenchymal Transition. *Clin. Cancer Res.* **2013**, *19*, 4392–4403.

126. Lieu, C.H.; Tran, H.; Jiang, Z.-Q.; Mao, M.; Overman, M.J.; Lin, E.; Eng, C.; Morris, J.; Ellis, L.; Heymach, J. V.; et al. The Association of Alternate VEGF Ligands with Resistance to Anti-VEGF Therapy in Metastatic Colorectal Cancer. *PLoS One* **2013**, *8*, e77117.

127. Carmeliet, P.; Li, X.; Treps, L.; Conradi, L.-C.; Loges, S. RAISEing VEGF-D's importance as predictive biomarker for ramucirumab in metastatic colorectal cancer patients. *Ann. Oncol.* **2018**.

128. Tiwari, P. Ramucirumab: Boon or bane. *J. Egypt. Natl. Canc. Inst.* **2016**, *28*, 133–140.

129. Makita, S.; Tobinai, K. Mogamulizumab for the treatment of T-cell lymphoma. *Expert Opin. Biol. Ther.* **2017**, *17*, 1145–1153.

130. van de Donk, N.W.C.J.; Usmani, S.Z. CD38 Antibodies in Multiple Myeloma: Mechanisms of Action and Modes of Resistance. *Front. Immunol.* **2018**, *9*.

131. Huang, L.; Fu, L. Mechanisms of resistance to EGFR tyrosine kinase inhibitors. *Acta Pharm. Sin. B* **2015**, *5*, 390–401.

132. Rexer, B.N.; Arteaga, C.L. Intrinsic and acquired resistance to HER2-targeted therapies in HER2 gene-amplified breast cancer: mechanisms and clinical implications. *Crit. Rev. Oncog.* **2012**, *17*, 1–16.

133. Brand, T.M.; Iida, M.; Stein, A.P.; Corrigan, K.L.; Braverman, C.M.; Luthar, N.; Toulany, M.; Gill, P.S.; Salgia, R.; Kimple, R.J.; et al. AXL Mediates Resistance to Cetuximab Therapy. *Cancer Res.* **2014**, *74*, 5152–5164.

134. Díaz-Serrano, A.; Sánchez-Torre, A.; Paz-Ares, L. Necitumumab for the treatment of advanced non-small-cell lung cancer. *Futur. Oncol.* **2019**, *15*, 705–716.

135. Bardelli, A.; Siena, S. Molecular Mechanisms of Resistance to Cetuximab and Panitumumab in Colorectal Cancer. *J. Clin. Oncol.* **2010**, *28*, 1254–1261.

136. Gagliato, D. de M.; Jardim, D.L.F.; Marchesi, M.S.P.; Hortobagyi, G.N. Mechanisms of resistance and sensitivity to anti-HER2 therapies in HER2+ breast cancer. *Oncotarget* **2016**, *7*.

137. Pohlmann, P.R.; Mayer, I.A.; Mernaugh, R. Resistance to Trastuzumab in Breast Cancer. *Clin. Cancer Res.* **2009**, *15*, 7479–7491.

138. Hosono, M.; Chatal, J. *Resistance to Ibritumomab in Lymphoma*; Hosono, M., Chatal, J.-F., Eds.; Resistance to Targeted Anti-Cancer Therapeutics; 1st ed.; Springer International Publishing, 2018; Vol. 18; ISBN 978-3-319-78237-9.

139. Baig, N.A.; Taylor, R.P.; Lindorfer, M.A.; Church, A.K.; LaPlant, B.R.; Pettinger, A.M.; Shanafelt, T.D.; Nowakowski, G.S.; Zent, C.S. Induced Resistance to Ofatumumab-Mediated Cell Clearance Mechanisms, Including Complement-Dependent Cytotoxicity, in Chronic Lymphocytic Leukemia. *J. Immunol.* **2014**, *192*, 1620–1629.

140. Sebejova, L.; Borsky, M.; Jaskova, Z.; Potesil, D.; Navrkalova, V.; Malcikova, J.; Sramek, M.; Doubek, M.; Loja, T.; Pospisilova, S.; et al. Distinct in vitro sensitivity of p53-mutated and ATM-mutated chronic lymphocytic leukemia cells to ofatumumab and rituximab. *Exp. Hematol.* **2014**, *42*, 867-874.e1.

141. Rezvani, A.R.; Maloney, D.G. Rituximab resistance. *Best Pract. Res. Clin. Haematol.* **2011**, *24*, 203–216.

142. Tamayo-Orbegozo, E.; Amo, L.; Riñón, M.; Nieto, N.; Amutio, E.; Maruri, N.; Solaun, M.; Arrieta, A.; Larrucea, S. Podocalyxin promotes proliferation and survival in mature B-cell non-Hodgkin lymphoma cells. *Oncotarget* **2017**, *8*.

143. Freeman, C.L.; Sehn, L.H. A tale of two antibodies: obinutuzumab versus rituximab. *Br. J. Haematol.* **2018**, *182*, 29–45.

144. Park, Y.-J.; Kuen, D.-S.; Chung, Y. Future prospects of immune checkpoint blockade in cancer: from response prediction to overcoming resistance. *Exp. Mol. Med.* **2018**, *50*, 109.

145. Jacquelot, N.; Roberti, M.P.; Enot, D.P.; Rusakiewicz, S.; Ternès, N.; Jegou, S.; Woods, D.M.; Sodré, A.L.; Hansen, M.; Meirow, Y.; et al. Predictors of responses to immune checkpoint blockade in advanced melanoma. *Nat. Commun.* **2017**, *8*, 592.

146. Keyel, M.E.; Reynolds, C.P. Spotlight on dinutuximab in the treatment of high-risk neuroblastoma: development and place in therapy. *Biol. Targets Ther.* **2018**, *Volume 13*, 1–12.

147. McDermott, D.F.; Huseni, M.A.; Atkins, M.B.; Motzer, R.J.; Rini, B.I.; Escudier, B.; Fong, L.; Joseph, R.W.; Pal, S.K.; Reeves, J.A.; et al. Clinical activity and molecular correlates of response to atezolizumab alone or in combination with bevacizumab versus sunitinib in renal cell carcinoma. *Nat. Med.* **2018**, *24*, 749–757.

148. Shukuya, T.; Carbone, D.P. Predictive Markers for the Efficacy of Anti–PD-1/PD-L1 Antibodies in Lung Cancer. *J. Thorac. Oncol.* **2016**, *11*, 976–988.

149. O'Donnell, J.S.; Long, G. V.; Scolyer, R.A.; Teng, M.W.L.; Smyth, M.J. Resistance to PD1/PDL1 checkpoint inhibition. *Cancer Treat. Rev.* **2017**, *52*, 71–81.

150. Sharma, P.; Hu-Lieskovan, S.; Wargo, J.A.; Ribas, A. Primary, Adaptive, and Acquired Resistance to Cancer Immunotherapy. *Cell* **2017**, *168*, 707–723.

151. Wallington-Beddoe, C.T.; Sobieraj-Teague, M.; Kuss, B.J.; Pitson, S.M. Resistance to proteasome inhibitors and other targeted therapies in myeloma. *Br. J. Haematol.* **2018**, *182*, 11–28.

152. Shah, N.N.; Fry, T.J. Mechanisms of resistance to CAR T cell therapy. *Nat. Rev. Clin. Oncol.*

2019, *16*, 372–385.

153. Gardner, R.; Wu, D.; Cherian, S.; Fang, M.; Hanafi, L.-A.; Finney, O.; Smithers, H.; Jensen, M.C.; Riddell, S.R.; Maloney, D.G.; et al. Acquisition of a CD19-negative myeloid phenotype allows immune escape of MLL-rearranged B-ALL from CD19 CAR-T-cell therapy. *Blood* **2016**, *127*, 2406–2410.

154. Köhnke, T.; Krupka, C.; Tischer, J.; Knösel, T.; Subklewe, M. Increase of PD-L1 expressing B-precursor ALL cells in a patient resistant to the CD19/CD3-bispecific T cell engager antibody blinatumomab. *J. Hematol. Oncol.* **2015**, *8*, 111.

155. Salzer, W.; Bostrom, B.; Messinger, Y.; Perissinotti, A.J.; Marini, B. Asparaginase activity levels and monitoring in patients with acute lymphoblastic leukemia. *Leuk. Lymphoma* **2018**, *59*, 1797–1806.

156. Turturro, F. Denileukin diftitox: a biotherapeutic paradigm shift in the treatment of lymphoid-derived disorders. *Expert Rev. Anticancer Ther.* **2007**, *7*, 11–17.

157. Linenberger, M.L. CD33-directed therapy with gemtuzumab ozogamicin in acute myeloid leukemia: progress in understanding cytotoxicity and potential mechanisms of drug resistance. *Leukemia* **2005**, *19*, 176–182.

158. García-Alonso, S.; Ocaña, A.; Pandiella, A. Resistance to Antibody–Drug Conjugates. *Cancer Res.* **2018**, *78*, 2159–2165.

159. Hergott, C.B.; Kim, A.S.; Wadleigh, M.; Lindsley, R.C. Resistance to Inotuzumab Ozogamicin in a B-ALL Patient with TET2 and DNMT3A Mutations and Myeloid Lineage Switch. *Blood* **2018**, *132*.

160. Takeshita, A.; Shinjo, K.; Yamakage, N.; Ono, T.; Hirano, I.; Matsui, H.; Shigeno, K.; Nakamura, S.; Tobita, T.; Maekawa, M.; et al. CMC-544 (inotuzumab ozogamicin) shows less effect on multidrug resistant cells: analyses in cell lines and cells from patients with B-cell chronic lymphocytic leukaemia and lymphoma. *Br. J. Haematol.* **2009**, *146*, 34–43.

161. Kinjyo, I.; Matlawska-Wasowska, K.; Chen, X.; Monks, N.R.; Burke, P.; Winter, S.S.; Wilson, B.S. Characterization of the anti-CD22 targeted therapy, moxetumomab pasudotox, for B-cell precursor acute lymphoblastic leukemia. *Pediatr. Blood Cancer* **2017**, *64*, e26604.

162. Hu, X.; Wei, H.; Xiang, L.; Chertov, O.; Wayne, A.S.; Bera, T.K.; Pastan, I. Methylation of the DPH1 promoter causes immunotoxin resistance in acute lymphoblastic leukemia cell line KOPN-8. *Leuk. Res.* **2013**, *37*, 1551–1556.

163. Wei, H.; Xiang, L.; Wayne, A.S.; Chertov, O.; FitzGerald, D.J.; Bera, T.K.; Pastan, I. Immunotoxin resistance via reversible methylation of the DPH4 promoter is a unique survival strategy. *Proc. Natl. Acad. Sci.* **2012**, *109*, 6898–6903.

164. Stephansky, J.; Togami, K.; Ghandi, M.; Montero, J.; VonEgypt, N.; Lindsay, R.; Brooks, C.; Aster, J.C.; Johannessen, C.; Lane, A.A. Resistance to SL-401 in AML and BPDCN Is Associated with Loss of the Diphthamide Synthesis Pathway Enzyme DPH1 and Is Reversible By Azacitidine. *Blood* **2017**, *130*.

165. Loganzo, F.; Sung, M.; Gerber, H.-P. Mechanisms of Resistance to Antibody–Drug Conjugates. *Mol. Cancer Ther.* **2016**.

166. Chen, R.; Hou, J.; Newman, E.; Kim, Y.; Donohue, C.; Liu, X.; Thomas, S.H.; Forman, S.J.; Kane,

S.E. CD30 Downregulation, MMAE Resistance, and MDR1 Upregulation Are All Associated with Resistance to Brentuximab Vedotin. *Mol. Cancer Ther.* **2015**.

167. Abdullah, S.E.; Perez-Soler, R. Mechanisms of resistance to vascular endothelial growth factor blockade. *Cancer* 2012.

168. Vandamme, T.; Beyens, M.; De Beeck, K.O.; Dogan, F.; Van Koetsveld, P.M.; Pauwels, P.; Mortier, G.; Vangestel, C.; DE Herder, W.; Van Camp, G.; et al. Long-term acquired everolimus resistance in pancreatic neuroendocrine tumours can be overcome with novel PI3K-AKT-mTOR inhibitors. *Br. J. Cancer* **2016**.

169. Harada, K.; Miyake, H.; Kumano, M.; Fujisawa, M. Acquired resistance to temsirolimus in human renal cell carcinoma cells is mediated by the constitutive activation of signal transduction pathways through mTORC2. *Br. J. Cancer* **2013**.

170. Engl, T.; Rutz, J.; Maxeiner, S.; Fanguen, S.; Juengel, E.; Koschade, S.; Roos, F.; Khoder, W.; Tsaur, I.; Blaheta, R.A. Acquired resistance to temsirolimus is associated with integrin α7 driven chemotactic activity of renal cell carcinoma *in vitro*. *Oncotarget* **2018**.

171. Juengel, E.; Makarević, J.; Reiter, M.; Mani, J.; Tsaur, I.; Bartsch, G.; Haferkamp, A.; Blaheta, R.A. Resistance to the mTOR inhibitor temsirolimus alters adhesion and migration behavior of renal cell carcinoma cells through an integrin α5- and integrin β3-dependent mechanism. *Neoplasia (United States)* **2014**.

172. Wafelman, A.; Hoefnagel, C.; Maes, R.A.; Beijnen, J. Radioiodinated metaiodobenzylguanidine: a review of its biodistribution and pharmacokinetics, drug interactions, cytotoxicity and dosimetry. *Eur. J. Nucl. Med.* **1994**, *21*.

173. Jiang, X.; Li, X.; Li, W.; Bai, H.; Zhang, Z. PARP inhibitors in ovarian cancer: Sensitivity prediction and resistance mechanisms. *J. Cell. Mol. Med.* 2019.

174. Gilabert, M.; Launay, S.; Ginestier, C.; Bertucci, F.; Audebert, S.; Pophillat, M.; Toiron, Y.; Baudelet, E.; Finetti, P.; Noguchi, T.; et al. Poly(ADP-ribose) polymerase 1 (PARP1) overexpression in human breast cancer stem cells and resistance to olaparib. *PLoS One* **2014**.

175. Maleki Vareki, S.; Rytelewski, M.; Figueredo, R.; Chen, D.; Ferguson, P.J.; Vincent, M.; Min, W.; Zheng, X.; Koropatnick, J. Indoleamine 2,3-dioxygenase mediates immune-independent human tumor cell resistance to olaparib, gamma radiation, and cisplatin. *Oncotarget* **2014**.

176. Jaspers, J.E.; Kersbergen, A.; Boon, U.; Sol, W.; Van Deemter, L.; Zander, S.A.; Drost, R.; Wientjens, E.; Ji, J.; Aly, A.; et al. Loss of 53BP1 causes PARP inhibitor resistance in BRCA1-mutated mouse mammary tumors. *Cancer Discov.* **2013**.

177. Kondrashova, O.; Nguyen, M.; Shield-Artin, K.; Tinker, A. V.; Teng, N.N.H.; Harrell, M.I.; Kuiper, M.J.; Ho, G.Y.; Barker, H.; Jasin, M.; et al. Secondary somatic mutations restoring RAD51C and RAD51D associated with acquired resistance to the PARP inhibitor rucaparib in high-grade ovarian carcinoma. *Cancer Discov.* **2017**.

178. Dockery, L.E.; Gunderson, C.C.; Moore, K.N. Rucaparib: The past, present, and future of a newly approved PARP inhibitor for ovarian cancer. *Onco. Targets. Ther.* 2017.

179. Tahir, S.K.; Smith, M.L.; Hessler, P.; Rapp, L.R.; Idler, K.B.; Park, C.H.; Leverson, J.D.; Lam, L.T. Potential mechanisms of resistance to venetoclax and strategies to circumvent it. *BMC Cancer* **2017**.

180. Woyach, J.A.; Johnson, A.J. Targeted therapies in CLL: Mechanisms of resistance and strategies for management. *Blood* **2015**.

181. Chyla, B.; Daver, N.; Doyle, K.; McKeegan, E.; Huang, X.; Ruvolo, V.; Wang, Z.; Chen, K.; Souers, A.; Leverson, J.; et al. Genetic biomarkers of sensitivity and resistance to venetoclax monotherapy in patients with relapsed acute myeloid leukemia. *Am. J. Hematol.* **2018**.

182. Lü, S.; Wang, J. The resistance mechanisms of proteasome inhibitor bortezomib. *Biomark. Res.* 2013.

183. Ao, L.; Wu, Y.; Kim, D.; Jang, E.R.; Kim, K.; Lee, D.M.; Kim, K.B.; Lee, W. Development of peptide-based reversing agents for P-glycoprotein-mediated resistance to carfilzomib. *Mol. Pharm.* **2012**.

184. Riz, I.; Hawley, T.S.; Hawley, R.G. KLF4-SQSTM1/p62-associated prosurvival autophagy contributes to carfilzomib resistance in multiple myeloma models. *Oncotarget* **2015**.

185. Zang, Y.; Kirk, C.J.; Johnson, D.E. Carfilzomib and oprozomib synergize with histone deacetylase inhibitors in head and neck squamous cell carcinoma models of acquired resistance to proteasome inhibitors. *Cancer Biol. Ther.* **2014**.

186. Chattopadhyay, N.; Berger, A.J.; Koenig, E.; Bannerman, B.; Garnsey, J.; Bernard, H.; Hales, P.; Lopez, A.M.; Yang, Y.; Donelan, J.; et al. KRAS genotype correlates with proteasome inhibitor ixazomib activity in preclinical in vivo models of colon and non-small cell lung cancer: Potential role of tumor metabolism. *PLoS One* **2015**.

187. Khan, I.; Halasi, M.; Patel, A.; Schultz, R.; Kalakota, N.; Chen, Y.-H.; Aardsma, N.; Liu, L.; Crispino, J.D.; Mahmud, N.; et al. FOXM1 contributes to treatment failure in acute myeloid leukemia. *JCI Insight* **2018**.

188. Tomita, A.; Kiyoi, H.; Naoe, T. Mechanisms of action and resistance to all-trans retinoic acid (ATRA) and arsenic trioxide (As2O3) in acute promyelocytic leukemia. *Int. J. Hematol.* **2013**.

189. Lin, J.H.; Kim, E.J.; Bansal, A.; Seykora, J.; Richardson, S.K.; Cha, X.Y.; Zafar, S.; Nasta, S.; Wysocka, M.; Benoit, B.; et al. Clinical and in vitro resistance to bexarotene in adult T-cell leukemia: Loss of RXR-{alpha} receptor. *Blood* **2008**.

190. Villasante, A.; Sakaguchi, K.; Kim, J.; Cheung, N.K.; Nakayama, M.; Parsa1, H.; Okano, T.; Shimizu, T.; Vunjak-Novakovic, G. Vascularized tissue-engineered model for studying drug resistance in neuroblastoma. *Theranostics* **2017**.

191. Schultze, E.; Ourique, A.; Yurgel, V.C.; Begnini, K.R.; Thurow, H.; De Leon, P.M.M.; Campos, V.F.; Dellagostin, O.A.; Guterres, S.R.; Pohlmann, A.R.; et al. Encapsulation in lipid-core nanocapsules overcomes lung cancer cell resistance to tretinoin. *Eur. J. Pharm. Biopharm.* **2014**.

192. Regazzi, M.B.; Iacona, I.; Gervasutti, C.; Lazzarino, M.; Toma, S. Clinical pharmacokinetics of tretinoin. *Clin. Pharmacokinet.* 1997.

193. Colao, A.; Auriemma, R.S.; Lombardi, G.; Pivonello, R. Resistance to somatostatin analogs in acromegaly. *Endocr. Rev.* 2011.

194. Kanagal-Shamanna, R.; Jain, P.; Patel, K.P.; Routbort, M.; Bueso-Ramos, C.; Alhalouli, T.; Khoury, J.D.; Luthra, R.; Ferrajoli, A.; Keating, M.; et al. Targeted multigene deep sequencing of Bruton tyrosine kinase inhibitor–resistant chronic lymphocytic leukemia with disease

progression and Richter transformation. *Cancer* **2019**.

195. Mertens, D.; Stilgenbauer, S. Ibrutinib-resistant CLL: Unwanted and unwonted! *Blood* 2017.

196. Galicia-Vázquez, G.; Aloyz, R. Ibrutinib Resistance Is Reduced by an Inhibitor of Fatty Acid Oxidation in Primary CLL Lymphocytes. *Front. Oncol.* **2018**.

197. Woyach, J.A.; Furman, R.R.; Liu, T.-M.; Ozer, H.G.; Zapatka, M.; Ruppert, A.S.; Xue, L.; Li, D.H.-H.; Steggerda, S.M.; Versele, M.; et al. Resistance Mechanisms for the Bruton's Tyrosine Kinase Inhibitor Ibrutinib. *N. Engl. J. Med.* **2014**, *370*, 2286–2294.

198. Hrustanovic, G.; Lee, B.J.; Bivona, T.G. Mechanisms of resistance to EGFR targeted therapies. *Cancer Biol. Ther.* 2013.

199. Wu, S.-G.; Liu, Y.-N.; Tsai, M.-F.; Chang, Y.-L.; Yu, C.-J.; Yang, P.-C.; Yang, J.C.-H.; Wen, Y.-F.; Shih, J.-Y. The mechanism of acquired resistance to irreversible EGFR tyrosine kinase inhibitor-afatinib in lung adenocarcinoma patients. *Oncotarget* **2016**.

200. Hashida, S.; Yamamoto, H.; Shien, K.; Miyoshi, Y.; Ohtsuka, T.; Suzawa, K.; Watanabe, M.; Maki, Y.; Soh, J.; Asano, H.; et al. Acquisition of cancer stem cell-like properties in non-small cell lung cancer with acquired resistance to afatinib. *Cancer Sci.* **2015**.

201. Kobayashi, Y.; Fujino, T.; Nishino, M.; Koga, T.; Chiba, M.; Sesumi, Y.; Ohara, S.; Shimoji, M.; Tomizawa, K.; Takemoto, T.; et al. EGFR T790M and C797S Mutations as Mechanisms of Acquired Resistance to Dacomitinib. *J. Thorac. Oncol.* **2018**.

202. Yao, Z.; Fenoglio, S.; Gao, D.C.; Camiolo, M.; Stiles, B.; Lindsted, T.; Schlederer, M.; Johns, C.; Altorki, N.; Mittal, V.; et al. TGF- IL-6 axis mediates selective and adaptive mechanisms of resistance to molecular targeted therapy in lung cancer. *Proc. Natl. Acad. Sci.* **2010**, *107*, 15535–15540.

203. Eichhorn, P.J.A.; Gili, M.; Scaltriti, M.; Serra, V.; Guzman, M.; Nijkamp, W.; Beijersbergen, R.L.; Valero, V.; Seoane, J.; Bernards, R.; et al. Phosphatidylinositol 3-kinase hyperactivation results in lapatinib resistance that is reversed by the mTOR/phosphatidylinositol 3-kinase inhibitor NVP-BEZ235. *Cancer Res.* **2008**.

204. Segovia-Mendoza, M.; González-González, M.E.; Barrera, D.; Díaz, L.; García-Becerra, R. Efficacy and mechanism of action of the tyrosine kinase inhibitors gefitinib, lapatinib and neratinib in the treatment of HER2-positive breast cancer: preclinical and clinical evidence. *Am. J. Cancer Res.* **2015**.

205. Hanker, A.B.; Brewer, M.R.; Sheehan, J.H.; Koch, J.P.; Sliwoski, G.R.; Nagy, R.; Lanman, R.; Berger, M.F.; Hyman, D.M.; Solit, D.B.; et al. An acquired HER2T798I gatekeeper mutation induces resistance to neratinib in a patient with HER2 mutant-driven breast cancer. *Cancer Discov.* **2017**.

206. Minari, R.; Bordi, P.; Tiseo, M. Third-generation epidermal growth factor receptor-tyrosine kinase inhibitors in T790M-positive non-small cell lung cancer: review on emerged mechanisms of resistance. *Transl. Lung Cancer Res.* **2017**.

207. Intlekofer, A.M.; Shih, A.H.; Wang, B.; Nazir, A.; Rustenburg, A.S.; Albanese, S.K.; Patel, M.; Famulare, C.; Correa, F.M.; Takemoto, N.; et al. Acquired resistance to IDH inhibition through trans or cis dimer-interface mutations. *Nature* **2018**.

208. Quek, L.; David, M.; Kennedy, A.; Metzner, M.; Amatangelo, M.; Shih, A.H.; Stoilova, B.;

Karamitros, D.; Quivoron, C.; Heiblig, M.; et al. Clonal Heterogeneity in Differentiation Response and Resistance to the IDH2 Inhibitor Enasidenib in Acute Myeloid Leukemia. *Blood* **2017**, *130*.

209. Abou Dalle, I.; DiNardo, C.D. The role of enasidenib in the treatment of mutant IDH2 acute myeloid leukemia. *Ther. Adv. Hematol.* **2018**.

210. Sahin, I. Ivosidenib in IDH1 -Mutated Acute Myeloid Leukemia. *N. Engl. J. Med.* **2018**, *379*, 1186–1186.

211. Deshpande, A.; Reddy, M.M.; Schade, G.O.M.; Ray, A.; Chowdary, T.K.; Griffin, J.D.; Sattler, M. Kinase domain mutations confer resistance to novel inhibitors targeting JAK2V617F in myeloproliferative neoplasms. *Leukemia* **2012**.

212. Yacoub, A.; Odenike, O.; Verstovsek, S. Ruxolitinib: Long-Term Management of Patients with Myelofibrosis and Future Directions in the Treatment of Myeloproliferative Neoplasms. *Curr. Hematol. Malig. Rep.* 2014.

213. Andreoli, A.; Verger, E.; Robin, M.; Raffoux, E.; Zini, J.-M.; Rousselot, P.; Socié, G.; Rea, D.; Parquet, N.; Giraudier, S.; et al. Clinical Resistance To Ruxolitinib Is More Frequent In Patients Without MPN-Associated Mutations and Is Rarely Due To Mutations In The JAK2 Kinase Drug-Binding Domain. *Blood* **2013**.

214. Kesarwani, M.; Huber, E.; Kincaid, Z.; Evelyn, C.R.; Biesiada, J.; Rance, M.; Thapa, M.B.; Shah, N.P.; Meller, J.; Zheng, Y.; et al. Targeting substrate-site in Jak2 kinase prevents emergence of genetic resistance. *Sci. Rep.* **2015**.

215. Hart, L.S.; Rader, J.A.; Raman, P.; Batra, V.; Russell, M.R.; Tsang, M.; Gagliardi, M.; Chen, L.; Martinez, D.; Li, Y.; et al. Preclinical therapeutic synergy of MEK1/2 and CDK4/6 inhibition in neuroblastoma. *Clin. Cancer Res.* **2017**, *23*, 1785–1796.

216. Woodfield, S.E.; Zhang, L.; Scorsone, K.A.; Liu, Y.; Zage, P.E. Binimetinib inhibits MEK and is effective against neuroblastoma tumor cells with low NF1 expression. *BMC Cancer* **2016**.

217. Kawakami, H.; Huang, S.; Pal, K.; Mukhopadhyay, D.; Sinicrope, F.A. Mutant BRAF upregulates MCL-1 to confer apoptosis resistance that is reversed by MCL-1 antagonism and cobimetinib in colorectal cancer. . *J. Clin. Oncol.* **2017**.

218. Zawistowski, J.S.; Bevill, S.M.; Goulet, D.R.; Stuhlmiller, T.J.; Beltran, A.S.; Olivares-Quintero, J.F.; Singh, D.; Sciaky, N.; Parker, J.S.; Rashid, N.U.; et al. Enhancer remodeling during adaptive bypass to MEK inhibition is attenuated by pharmacologic targeting of the P-TEFb complex. *Cancer Discov.* **2017**.

219. Wagner, S.; Vlachogiannis, G.; De Haven Brandon, A.; Valenti, M.; Box, G.; Jenkins, L.; Mancusi, C.; Self, A.; Manodoro, F.; Assiotis, I.; et al. Suppression of interferon gene expression overcomes resistance to MEK inhibition in KRAS-mutant colorectal cancer. *Oncogene* **2019**.

220. Py, C.; Christinat, Y.; Kreutzfeldt, M.; McKee, T.A.; Dietrich, P.-Y.; Tsantoulis, P. Response of NF1- Mutated Melanoma to an MEK Inhibitor. *JCO Precis. Oncol.* **2018**.

221. Gentry, L.; Samatar, A.A.; Der, C.J. Inhibitors of the ERK Mitogen-Activated Protein Kinase Cascade for Targeting RAS Mutant Cancers. In *Enzymes*; 2013.

222. Verykiou, S.; Alexander, M.; Edwards, N.; Plummer, R.; Chaudhry, B.; Lovat, P.E.; Hill, D.S. Harnessing autophagy to overcome mitogen-activated protein kinase kinase inhibitor-induced

resistance in metastatic melanoma. *Br. J. Dermatol.* **2019**.

223. McMahon, C.M.; Canaani, J.; Rea, B.; Sargent, R.L.; Morrissette, J.J.D.; Lieberman, D.B.; Watt, C.; Schwartz, G.W.; Faryabi, R.B.; Ferng, T.T.; et al. Mechanisms of Acquired Resistance to Gilteritinib Therapy in Relapsed and Refractory FLT3 -Mutated Acute Myeloid Leukemia. *Blood* **2017**.

224. Lee, L.Y.; Hernandez, D.; Rajkhowa, T.; Smith, S.C.; Raman, J.R.; Nguyen, B.; Small, D.; Levis, M. Preclinical studies of gilteritinib, a next-generation FLT3 inhibitor. *Blood* **2017**, *129*, 257–260.

225. Ueno, Y.; Mori, M.; Kamiyama, Y.; Saito, R.; Kaneko, N.; Isshiki, E.; Kuromitsu, S.; Takeuchi, M. Evaluation of gilteritinib in combination with chemotherapy in preclinical models of FLT3-ITD+ acute myeloid leukemia. *Oncotarget* **2019**, *10*.

226. Watanabe-Smith, K.; Rosenberg, M.; Bucy, T.; Tyner, J.W.; Borate, U. Factors Predicting Response and Resistance to Midostaurin in FLT3 Positive and FLT3 Negative AML in 483 Primary AML Patient Samples. *Blood* **2017**, *130*.

227. Williams, A.B.; Nguyen, B.; Li, L.; Brown, P.; Levis, M.; Leahy, D.; Small, D. Mutations of FLT3/ITD confer resistance to multiple tyrosine kinase inhibitors. *Leukemia* **2013**.

228. Gallogly, M.M.; Lazarus, H.M.; Cooper, B.W. Midostaurin: a novel therapeutic agent for patients with FLT3-mutated acute myeloid leukemia and systemic mastocytosis. *Ther. Adv. Hematol.* **2017**.

229. Yokoyama, N.; Matsunobu, T.; Matsumoto, Y.; Fukushi, J.I.; Endo, M.; Hatano, M.; Nabeshima, A.; Fukushima, S.; Okada, S.; Iwamoto, Y. Activation of ERK1/2 Causes Pazopanib Resistance via Downregulation of DUSP6 in Synovial Sarcoma Cells. *Sci. Rep.* **2017**.

230. Lanzi, C.; Bo, L.D.; Favini, E.; Tortoreto, M.; Beretta, G.L.; Arrighetti, N.; Zaffaroni, N.; Cassinelli, G. Overactive IGF1/insulin receptors and NRASQ61R mutation drive mechanisms of resistance to pazopanib and define rational combination strategies to treat synovial sarcoma. *Cancers (Basel).* **2019**.

231. Gotink, K.J.; Rovithi, M.; de Haas, R.R.; Honeywell, R.J.; Dekker, H.; Poel, D.; Azijli, K.; Peters, G.J.; Broxterman, H.J.; Verheul, H.M.W. Cross-resistance to clinically used tyrosine kinase inhibitors sunitinib, sorafenib and pazopanib. *Cell. Oncol.* **2015**.

232. Strumberg, D.; Schultheis, B. Regorafenib for cancer. *Expert Opin. Investig. Drugs* **2012**, *21*, 879–889.

233. Mirone, G.; Perna, S.; Shukla, A.; Marfe, G. Involvement of Notch-1 in Resistance to Regorafenib in Colon Cancer Cells. *J. Cell. Physiol.* **2016**.

234. Hedayat, S.; Khan, K.; Cunningham, D.; Vlachogiannis, G.; Marchetti, S.; Fassan, M.; Begum, R.; Schirripa, M.; Loupakis, F.; Valeri, N. PO-472 Microrna as biomarkers of resistance to regorafenib in metastatic colorectal cancer patient.; 2018.

235. Lin, X.L.; Xu, Q.; Tang, L.; Sun, L.; Han, T.; Wang, L.W.; Xiao, X.Y. Regorafenib inhibited gastric cancer cells growth and invasion via CXCR4 activated Wnt pathway. *PLoS One* **2017**.

236. Rajandram, R.; Ong, T.A.; Saad, M. *Resistance of Renal Cell Carcinoma to Targeted Therapy*; 2014; ISBN 978-1-63117-131-4.

237. Kerkela, R.; Woulfe, K.C.; Durand, J.-B.; Vagnozzi, R.; Kramer, D.; Chu, T.F.; Beahm, C.; Chen, M.H.; Force, T. Sunitinib-Induced Cardiotoxicity Is Mediated by Off-Target Inhibition of AMP-

Activated Protein Kinase. *Clin. Transl. Sci.* **2009**, *2*, 15–25.

238. Nakaoku, T.; Kohno, T.; Araki, M.; Niho, S.; Chauhan, R.; Knowles, P.P.; Tsuchihara, K.; Matsumoto, S.; Shimada, Y.; Mimaki, S.; et al. A secondary RET mutation in the activation loop conferring resistance to vandetanib. *Nat. Commun.* **2018**.

239. Terzyan, S.S.; Shen, T.; Liu, X.; Huang, Q.; Teng, P.; Zhou, M.; Hilberg, F.; Cai, J.; Mooers, B.H.M.; Wu, J. Structural basis of resistance of mutant RET protein tyrosine kinase to its inhibitors nintedanib and vandetanib. *J. Biol. Chem.* **2019**.

240. Drilon, A.; Laetsch, T.W.; Kummar, S.; DuBois, S.G.; Lassen, U.N.; Demetri, G.D.; Nathenson, M.; Doebele, R.C.; Farago, A.F.; Pappo, A.S.; et al. Efficacy of Larotrectinib in TRK Fusion–Positive Cancers in Adults and Children . *N. Engl. J. Med.* **2018**.

241. Nakagawa, T.; Matsushima, T.; Kawano, S.; Nakazawa, Y.; Kato, Y.; Adachi, Y.; Abe, T.; Semba, T.; Yokoi, A.; Matsui, J.; et al. Lenvatinib in combination with golvatinib overcomes hepatocyte growth factor pathway-induced resistance to vascular endothelial growth factor receptor inhibitor. *Cancer Sci.* **2014**.

242. Capozzi, M.; De Divitiis, C.; Ottaiano, A.; von Arx, C.; Scala, S.; Tatangelo, F.; Delrio, P.; Tafuto, S. <p>Lenvatinib, a molecule with versatile application: from preclinical evidence to future development in anti-cancer treatment</p>. *Cancer Manag. Res.* **2019**.

243. Nishio, M.; Horai, T.; Horiike, A.; Nokihara, H.; Yamamoto, N.; Takahashi, T.; Murakami, H.; Yamamoto, N.; Koizumi, F.; Nishio, K.; et al. Phase 1 study of lenvatinib combined with carboplatin and paclitaxel in patients with non-small-cell lung cancer. *Br. J. Cancer* **2013**.

244. Hudson, C.D.; Hagemann, T.; Mather, S.J.; Avril, N. Resistance to the tyrosine kinase inhibitor axitinib is associated with increased glucose metabolism in pancreatic adenocarcinoma. *Cell Death Dis.* **2014**.

245. Zabriskie, M.S.; Eide, C.A.; Yan, D.; Vellore, N.A.; Pomicter, A.D.; Savage, S.L.; Druker, B.J.; Deininger, M.W.; O'Hare, T. Extreme mutational selectivity of axitinib limits its potential use as a targeted therapeutic for BCR-ABL1-positive leukemia. *Leukemia* **2016**.

246. Bronte, G.; Ulivi, P.; Verlicchi, A.; Cravero, P.; Delmonte, A.; Crinò, L. <p>Targeting RET-rearranged non-small-cell lung cancer: future prospects</p>. *Lung Cancer Targets Ther.* **2019**.
